# Comparative genomics in cyprinids: common carp ESTs help the annotation of the zebrafish genome

**DOI:** 10.1186/1471-2105-7-S5-S2

**Published:** 2006-12-18

**Authors:** Alan Christoffels, Richard Bartfai, Hamsa Srinivasan, Hans Komen, Laszlo Orban

**Affiliations:** 1 Computational Biology Group, Temasek Life Sciences Laboratory, Singapore; 2 School of Biological Sciences, Nanyang Technological University, Singapore; 3 Reproductive Genomics Group, Temasek Life Sciences Laboratory, Singapore; 4 Animal Breeding and Genetics Group, Wageningen University, Wageningen, The Netherlands; 5 Department of Biological Sciences, The National University of Singapore, Singapore

## Abstract

**Background:**

Automatic annotation of sequenced eukaryotic genomes integrates a combination of methodologies such as *ab-initio *methods and alignment of homologous genes and/or proteins. For example, annotation of the zebrafish genome within Ensembl relies heavily on available cDNA and protein sequences from two distantly related fish species and other vertebrates that have diverged several hundred million years ago. The scarcity of genomic information from other cyprinids provides the impetus to leverage EST collections to understand gene structures in this diverse teleost group.

**Results:**

We have generated 6,050 ESTs from the differentiating testis of common carp *(Cyprinus carpio) *and clustered them with 9,303 non-gonadal ESTs from CarpBase as well as 1,317 ESTs and 652 common carp mRNAs from GenBank. Over 28% of the resulting 8,663 unique transcripts are exclusively testis-derived ESTs. Moreover, 974 of these transcripts did not match any sequence in the zebrafish or fathead minnow EST collection.

A total of 1,843 unique common carp sequences could be stringently mapped to the zebrafish genome (version 5), of which 1,752 matched coding sequences of zebrafish genes with or without potential splice variants. We show that 91 common carp transcripts map to intergenic and intronic regions on the zebrafish genome assembly and regions annotated with non-teleost sequences. Interestingly, an additional 42 common carp transcripts indicate the potential presence of new splicing variants not found in zebrafish databases so far. The fact that common carp transcripts help the identification or confirmation of these coding regions in zebrafish exemplifies the usefulness of sequences from closely related species for the annotation of model genomes.

We also demonstrate that 5' UTR sequences of common carp and zebrafish orthologs share a significant level of similarity based on preservation of motif arrangements for as many as 10 *ab-initio *motifs.

**Conclusion:**

Our data show that there is sufficient homology between the transcribed sequences of common carp and zebrafish to warrant an even deeper cyprinid transcriptome comparison. On the other hand, the comparative analysis illustrates the value in utilizing partially sequenced transcriptomes to understand gene structure in this diverse teleost group. We highlight the need for integrated resources to leverage the wealth of fragmented genomic data.

## Background

Eukaryotic gene prediction has been a challenging problem, explored over the last two decades and driven by the availability of large volumes of genomic data. The development of gene prediction methods have traditionally included (1) *ab-initio *approaches such as GENSCAN [1,2] that do not use any experimental evidence, (2) alignment-based methods such as GENEWISE [3] that attempts to align an homologous protein sequence to a genomic sequence and more recently, (3) hybrid approaches that incorporate cDNA-defined splice junctions into *ab-initio *and protein alignment information [3-5]. Such hybrid approaches for automatic annotation of genome sequences have been implemented within the Ensembl annotation project [6,7]. Ensembl represents a bioinformatics project aimed at annotating sequenced genomes and integrating biological data that can be mapped or assigned to features described in the genomic data.

At present, twenty fully or near-fully sequenced vertebrate genomes have been included in Ensembl (version 39). Teleosts, comprising about half the number of all extant vertebrate species, are represented by only five species, namely Japanese fugu (*Takifugu rubripes*), green spotted pufferfish (*Tetraodon nigroviridis*), zebrafish (*Danio rerio*), Japanese medaka (*Oryzias latipes*) and three-spined stickleback (*Gastroceus aculeatus*), within the Ensembl data.

The zebrafish is a representative of the most abundant and widespread primary freshwater fish family, *Cyprinidae *[8,9] with ample genomic resources including a nearly fully sequenced genome and over a million expressed sequence tags (ESTs). However, genomic data for the rest of the cyprinids are quite scarce (for review see [10]), partly due to polyploidy that represents a characteristic feature of several members of the *Cyprinidae *family [11,12].

In the absence of genome projects from closely related species, the automatic annotation of genomes relies heavily on available cDNA and protein sequences of other vertebrates for sequence comparisons. For example, mammalian and teleost genome comparisons have been used successfully to identify conserved protein-coding genes and regulatory elements despite the 450 million years that elapsed since their last common ancestor [13,14]. In contrast, a recent study by Thomas and colleagues [15] concluded that fish-mammal comparisons were unable to detect most non-coding regions that were conserved between amniotes. Theoretically, the annotation of the zebrafish genome could benefit from sequence data for a closely related species excluding the annotated genomes of Japanese fugu and the green spotted pufferfish that share a common ancestor with zebrafish more than 200 million years ago [16].

The UniGene collection [17] represents a database of species-specific mRNA and ESTs that are grouped into clusters or genes based on stringent sequence identity. Currently two cyprinid species are present in the UniGene collection (build 90 [17]), namely the zebrafish and fathead minnow *(Pimephales promelas)*. Zebrafish belongs to the subfamily *Rasborinae*, whereas fathead minnow is a member of *Leuciscinae *[18]. Nearly 11,000 ESTs are present in dbEST [19,20] for a third cyprinid species, common carp *(Cyprinus carpio, Cyprininae) *[18], however they were not sampled in the recent UniGene collection (build 90). (These common carp ESTs have been produced earlier by other teams from a range of tissues other than gonad [21]). Common carp is the most important fish species of freshwater aquaculture, probably with the earliest domestication records among fishes [22,23]. It has been used in fish biology and aquaculture research quite extensively (for reviews see [24,25]).

Common carp is a close relative of the zebrafish, they both belong to the same family. The ancestors of common carp and zebrafish have split about 50 million years ago (Mya) [16], whereas the corresponding divergence data for fathead minnow is not available. The wealth of EST data for these three cyprinid species and the recent speciation event provides a valuable resource to aid the ongoing zebrafish genome annotation project.

In order to facilitate the comparative genomic analysis of gonad development in cyprinid teleosts, primarily the zebrafish [26] and common carp, we set out to complement the non-gonadal common carp transcriptome data by sequencing clones from testis-derived cDNA libraries. We then performed a cross-species analysis of cyprinids by comparing common carp ESTs sequences to those originating from zebrafish and fathead minnow, as well as to the partially sequenced zebrafish genome. We mapped common carp ESTs to un-annotated regions of the zebrafish genome. Our results identified novel testis-expressed transcripts in cyprinids and new splice variants in the common carp transcriptome. We were able to show that the two species share a significant level of similarity in the 5'UTR regions. Collectively, these results indicate that such a comparative approach, based on the usage of closely related species, could add value to the current ongoing improvements to the zebrafish genome assembly and annotation by the genomic community.

## Results and Discussion

### Testis-derived common carp cDNAs add nearly 2,500 unique sequences to the public EST collection

At the start of our work GenBank [27] and CarpBASE [21] together contained 10,615 common carp ESTs, all of which originated from non-gonadal cDNA libraries. We enriched the existing transcriptome dataset for common carp, by generating an additional 6,050 ESTs by random sequencing of clones from five different cDNA libraries derived from differentiating common carp testis (60–100 days post fertilization or dpf; see Additional File 1: Table S1 for details on the libraries). We also added an additional 652 common carp mRNAs extracted from GenBank in order to assist the assembly of ESTs.

**Figure 1:**
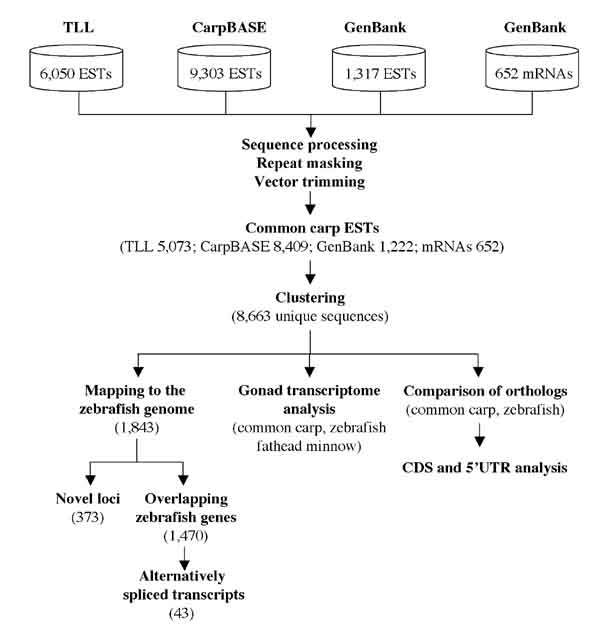
Flow chart depicting the protocols used for comparing the sequences from common carp and zebrafish.

**Figure 2:**
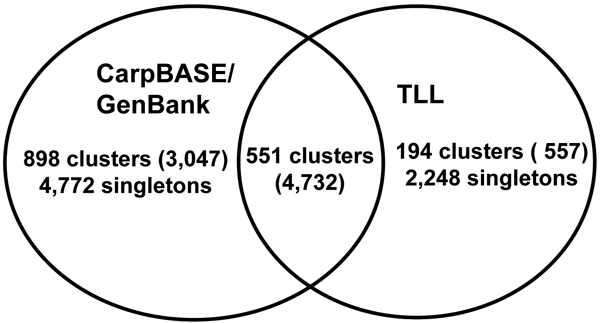
Distribution of clusters and singletons according to the origin of the sequences. The combined common carp collection was thoroughly cleaned and clustered using the STACKPACK clustering tool.

Following cleaning and quality control, over 15,000 ESTs {10,283 from GenBank plus CarpBASE and 5,073 from our own data (GenBank: DW719352–DW724424)} were retained and clustered (Fig. 1). The clustered dataset of 8,663 unique sequences (1,643 clusters and 7,020 singletons) contained 2,442 (28.1%) "testis-only" sequences, including clusters with exclusively testis-derived ESTs and singletons isolated from one of the testis cDNA libraries (Fig. 2).

In order to initiate functional annotation of the partial transcriptome of common carp, we identified open reading frames (ORFs) in our clustered EST set using ESTScan [28]. An ORF prediction was obtained for 81% of the clusters and 47.5% of the singletons, yielding a total of 4,663 sequences (data not shown). The ORF-containing common carp transcripts were classified into functional categories using protein domain databases (Additional File 2: Table S2; see Materials and Methods for databases used).

### Mapping of common carp ESTs to the zebrafish genome

In the zebrafish Ensembl annotation (Ensembl_37) genes were annotated using mRNA and proteins from the target species as well as a range of other vertebrates, the closest to zebrafish being Japanese fugu and green spotted pufferfish. We mapped our common carp EST data to the zebrafish genome assembly (v5; [29]) according to a multi-step protocol (see Materials and Methods for details and Additional File 8: Figure S1 for flow chart). A total of 1,182 common carp clusters (72% of all clusters) and 3,827 singletons (55% of all singletons) showed sequence similarity to the zebrafish genome with a BLAST E-value cutoff of 1e-04. After stringent filtering – selecting a unique zebrafish genomic location for each mapped common carp cluster (see Materials and Methods for detailed description) and sequence identity of 80% over 70% of the EST length – we assigned 484 clusters (29%) and 1,359 singletons (19%) to the zebrafish genome assembly (from here onwards these sequences will be referred to as "mapped common carp transcripts"). The common carp transcript map coordinates are available from Ensembl version 38 as a DAS track [30].

The 90 percentile of all intron lengths within the zebrafish Ensembl database is 4,657 nucleotides. There were 122 cases, where two common carp clusters/singletons mapped to the zebrafish genome within 4,657 nucleotides. These represent cases where the clusters and/or singletons potentially correspond to the same gene but were partitioned into separate clusters because of the absence of sequence data in the EST database.

Interestingly, there were 84 cases, where at least two clusters and/or singletons overlapped the same zebrafish locus. These represent potential gene family expansions in the common carp relative to zebrafish, but would require experimental validation in the future. These cases provide support for the incorporation of EST sequences from closely related "sequence-poor" species into the analysis pipeline of (nearly) completely sequenced genomes.

### Common carp ESTs map to regions lacking expressed sequence information in the zebrafish genome

Nearly 40% of ESTs obtained from GenBank and those sequenced in our lab are bi-directional due to the EST sequencing protocol used. As a result, the strandedness of the genome-aligned common carp ESTs were obtained using the splice-site orientation as defined in the EST2GENOME algorithm [31]. To identify un-annotated regions in the zebrafish genome, we required both plus and minus strands of the zebrafish genome be free of any sequence similarity features to non-common carp cDNA and proteins.

Of the 1,843 common carp transcripts mapped to the zebrafish genome assembly (Ensembl_37), 1,752 overlapped zebrafish cDNAs supported by genes and/or ESTs. The remaining 91 "mapped common carp transcripts" showed sequence identity to regions overlapping zebrafish introns (23), *ab-initio *predictions (22), non-zebrafish exons (22), intergenic regions (13) and non-zebrafish introns (11) (Additional File 3: Table S3; see Materials and Methods for classification criteria).

Five of the 13 common carp transcripts that map to intergenic regions are located less than 1 kb from the 5' end of the nearest neighbouring gene. Considering their close proximity to an annotated gene, these common carp transcripts represent potential untranslated regions (UTRs). In fact, the five neighbouring genes are annotated as developmental genes (data not shown). Developmental genes are highly conserved among species and very often the sequence conservation extends to their regulatory regions [13,14]. Furthermore, each of the common carp transcripts mapped to the zebrafish genome have sequence identity in excess of 80%, suggesting that the use of a lower threshold for common carp EST mapping might retrieve many more UTR sequences that could be subjected to similar UTR analyses as described in the Materials and Methods. The remaining eight common carp transcripts that map to intergenic regions are located between 5 and 150 kb away from the nearest zebrafish locus, suggesting the presence of novel gene loci that require experimental verification in the future.

Forty-two of the 91 mapped common carp transcripts have not been identified in the zebrafish and fathead minnow EST collections so far, therefore they represented novel cyprinid sequences. Another 16 of the 91 mapped common carp transcripts showed significant sequence similarity to the zebrafish and fathead minnow UniGene collection (build 91). (This indicated that the overlapping zebrafish transcripts might not have been available at the time of annotating the zebrafish genome version 5.). The remaining 33 common carp transcripts shared very weak sequence similarity (<40% identity) with either zebrafish or fathead minnow, thus might point to genes that diverged from their orthologs. Alternatively, these transcripts could represent sequences orthologous to zebrafish UTRs that are yet to be assigned to the annotated zebrafish genome.

The above cases illustrate the potential advantages of utilizing partial transcriptomes from related species in order to provide information on the functional properties of (a) un-annotated parts of genomes to be assembled as well as (b) regions annotated with distantly related species.

### Alternative exon usage identified by comparing cyprinid transcripts

EST-based analysis of alternative splicing has been performed earlier in mammals; the results suggest that 40–60% of the genes produce alternatively spliced transcripts [32]. Only a few studies have been performed on fish sequences (see e.g. [33,34]) resulting in a limited amount of data on splicing from teleosts.

**Figure 3:**
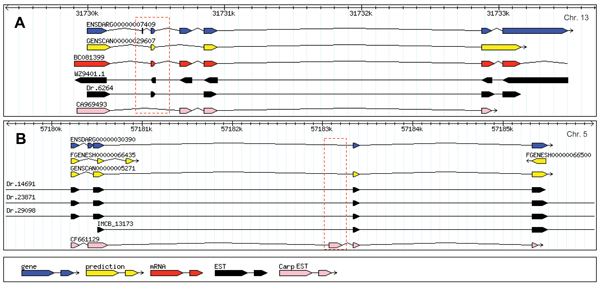
Examples of potential splice variants identified by mapping common carp transcripts to the zebrafish genome. (a) Common carp transcript lacking an exon present in the zebrafish cDNA. (b) A common carp transcript with exons not present in the overlapping zebrafish gene.

Interestingly, out of 1,752 common carp transcripts that map to coding regions, there were 26 cases where the exon structure showed evidence for a missing exon compared to the overlapping zebrafish Ensembl gene (example: Fig. 3A; full list: Additional File 4: Table S4). Similar comparisons yielded 16 cases where an exon that was present in the overlapping common carp ESTs was missing from the zebrafish transcript (example: Fig. 3B; full list: Additional File 4: Table S4). There are four possibilities to explain such differences: i) the exon in question is missing from one of the two genomes; ii) exclusive usage of different splice products in the two related species; iii) different preferences of alternative splice products; and iv) virtual difference due to partial transcriptomes.

At the time of submission GenBank contained over 1.3 million ESTs for zebrafish, fathead minnow and common carp. We propose that the broad mRNA diversity contained in teleost EST resources could be leveraged to understand the extent of alternative splicing within this diverse group of teleosts using analyses similar to those reported for human ESTs [35].

### Comparison of the partial cyprinid gonad transcriptomes identifies 974 novel testis-derived transcripts

The UniGene collection (build 91) contains datasets for two cyprinid species namely the zebrafish and fathead minnow. The common carp EST data reported in this study, sampled by nearly 9,000 unique transcripts, represent an additional cyprinid species that will be included in subsequent UniGene releases. The new EST data for common carp has also provided an opportunity to examine the value of tissue-specific sequencing on the existing gene collections. The common carp EST data were compared to the zebrafish UniGene collection (build 91) and subsequently to the fathead minnow data set using a BLAST E-value <1e-04 and sequence identity over 40% of the sequence length.

A total of 932 testis-derived common carp singletons and 42 clusters containing exclusively testis-derived common carp ESTs (Additional File 5: Table S5) did not overlap any of the zebrafish and fathead minnow UniGene transcripts. This dataset added 974 potentially novel sequences to the combined testis transcriptome of cyprinid teleosts (a fraction of these might represent UTR or coding sequences that are derived from fast-evolving genes).

A total of 214 out of 974 testis-only transcripts contained an ORF. Among these 214 transcripts, three testis-derived clusters contained an interleukin-8-like domain (IPR001811). The absence of significant sequence identity to zebrafish and fathead minnow at the nucleotide level is partly due to cytokines representing rapidly diverging genes involved in regulation of the immune system. Another domain, tissue inhibitor of metalloproteinase (IPR001820), which was identified in a common carp testis-derived transcript was present in two zebrafish UniGene clusters (Dr.240 and Dr.31907), but not sampled by any gonad-derived zebrafish sequences. The remaining 210 common carp transcripts do not show the presence of any characterized domains. These unique testis-derived transcripts could provide starting material for the isolation of their zebrafish orthologs, if any, and their potential application as markers for functional studies on gonad differentiation.

The potential homologs of 474 common carp clusters with at least one testis-derived EST were identified in the zebrafish UniGene data collection (Additional File 6: Table S6). When compared to the fathead minnow EST collection, six of these 474 common carp clusters showed sequence identity to adult testis-derived ESTs only (Additional File 6: Table S6). The common carp data correspond to differentiating testis (60–100 dpf), whereas the testis-derived zebrafish and fathead minnow clones presently found in the public databases are all from an adult organ. Therefore our results have complemented the previously available knowledge about the expression of these genes with experimental data on their activity during testis differentiation, providing indications on potentially conserved aspects of cyprinid gonad development. Moreover, the fact that common carp transcripts help the identification or confirmation of these coding regions in zebrafish exemplifies the usefulness of sequences from closely related species for the annotation of model genomes.

### Comparing the overall architecture of UTR regions for a set of orthologous genes from common carp and zebrafish

There is an average 82% sequence identity between the coding region of homologous gene pairs in zebrafish and common carp, whereas the same value for their 5' and 3' UTRs is only 61% and 58%, respectively (see Materials and Methods for details). We set out to explore the extent to which common carp and zebrafish retained similarity in the 5'UTR regions of their orthologous genes as this can reveal aspects of regulatory roles of these regions in both species. This task was difficult for two reasons: i) the fact that only limited sequence information is available from common carp dramatically decreased our ability to identify large number of orthologs between these species; and ii) the usual approaches to evaluate similarity based on local alignments are not really suitable for the similarity assessment of regulatory regions as demonstrated by Blanco and colleagues [36].

By analyzing 48 pairs of orthologous sequences and an additional six paralogs, which contained at least 50 bp at their 5' UTR (see Additional File 7: Table S7 for the complete list) we identified motif families shared in the 5' UTR of common carp and zebrafish mRNAs. Analyzing each of the orthologous pairs individually (plus the paralogs, whenever applicable), we determined the order of a maximum of 10 shared motifs between common carp and zebrafish.

**Figure 4:**
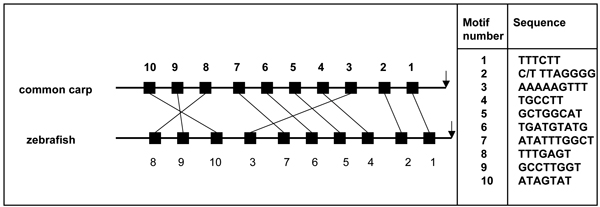
Collection of motifs identified in the *cdx1-cdx4 *ortholog gene pair in the common carp and zebrafish. The arrangement of motifs (black boxes) identified in the 5' UTR regions of common carp *cdx1 *and zebrafish *cdx4 *genes (caudal type homeobox transcription factor 4 orthologs) are shown on the left. A black arrow indicates the start of the first coding exon. Motif sequences are shown on the right.

The distribution of coverage for all orthologous pairs relative to the number of motifs in these arrangements is represented in Additional File 9: Figure S2. About two-third of the orthologous 5'UTR pairs tested shared 4–6 motifs in the conserved positional arrangement, whereas most of the rest shared 7–10. The distribution of identified motifs together with the conserved arrangement in the zebrafish caudal type homeobox transcription factor 4 (*cdx4*) (RefSeq:NM_131109) and its common carp ortholog, *cdx1 *(Genbank:X80668) are shown in Figure 4 as an example.

A detailed UTR analysis is not within the scope of the present manuscript, therefore we propose a large-scale analysis to find out whether 5'UTR regions from different orthologous pairs share motifs from the same family. The presence of such shared motif families would suggest the existence of regulatory components common to both species suitable for further evaluation.

## Conclusion

In this study, we have demonstrated the value of using ESTs for comparative analysis of transcriptomes from species with vastly different amount of sequence information. For example, common carp ESTs were successfully mapped to un-annotated regions of the zebrafish genome demonstrating the value of using closely related species for sequence comparison. The existing cyprinid ESTs represent a useful resource for comparative genomics to understand the evolution of this family.

Sequenced genomes are being integrated with functional information (e.g. expression data from microarray hybridisations, gene ontologies, etc.) to improve the efficiency of data mining. However, integrating fragmented genomic data for non-sequenced genomes remain a challenge for scientists who want to leverage inter-species comparisons. We suggest that there is a need to co-ordinate the isolated "in-house" integration attempts across laboratories in order to maximize and improve the quality of the information content that is currently under-utilized.

## Materials and methods

### Isolation of differentiating testis from common carp individuals

Androgenetic common carp "supermales" (YY; [37]) have been crossed with wild type females (XX) to give rise to an all-male offspring population. (This approach allows for testis isolation without the need for sexing the fish.) The gonad has been isolated from a minimum of 6 individuals at 59/60 (a mixture of 59 and 60 days-old individuals), 70, 80 and 100 dpf, respectively. One of the two gonads from each individual has been processed for histological analyses (data not shown), while the other one has been stored in RNAlater (Ambion) for the use of RNA isolation.

### Construction of cDNA libraries from the differentiating carp testis

Total RNA was isolated from the testis of 59/60, 80 and 100 days-old individuals, respectively. Full-length cDNA was synthesized using Creator Smart Library Construction Kit (Clontech) according to the manufacturer's instruction. After *SfiI *restriction enzyme digestion the adaptors and short cDNAs were removed by ChromaSpin 400 column (Clontech). The size fractionated cDNA pool was then cloned into a pBluescript based vector (detailed map is available on request) and transformed into *E. coli *XL10-Gold cells. Clones were picked into thirty, twenty and ten 96-well plates from the libraries generated from testes collected at 60, 80 and 100 dpf, respectively, and their insert was sequenced using M13 forward primer as described in [26].

Total RNA was isolated from the testis of 70 and 100 day old individuals, respectively. Two sets of subtractive hybridizations were performed: 70 dpf male gonad (driver) from 100 dpf testis (tester), and 100 dpf testis (d) from 70 dpf male gonad (t). The PCR-Select™ cDNA subtraction kit (Clontech) was used to enrich for developmental stage-specific fragments from the SMART cDNA template according to the recommendations of the manufacturer. The selectively amplified cDNA fragments (in average 400–800 bp in length) were ligated into pGEM-T (Promega) cloning vector. In total 2,500 clones have been picked from the two libraries and their insert was sequenced using M13 forward primer.

### Sequence acquisition and EST clustering

A total of 10,620 common carp ESTs, sequenced from a range of tissues other than gonad, were downloaded from GenBank (26 April 2005); 9,303 of those sequences are also available from CarpBASE [21] (see Additional File 1: Table S1 for details of clone origins). They were combined with 652 mRNAs from GenBank and with 6,050 gonad-derived common carp ESTs generated in our labs within the framework of this project (Fig. 1). Low quality regions were trimmed at the 3' end of ESTs prior to masking against libraries of repeats, mitochondrial and ribosomal sequences using RepeatMasker [38]. Sequences that comprised at least 70% unmasked nucleotides (10,283 GenBank and CarpBASE ESTs and mRNAs, 5,073 TLL ESTs) were retained for further analysis. (The processed TLL ESTs were submitted to GenBank and can be found under the following IDs: DW719352-DW724424.) The combined EST data set was clustered using the STACKPACK clustering tools [39,40] on HPCompaq Alpha ES40 architecture.

### Functional characterization of common carp transcripts

Common carp transcripts (clusters and singletons) were partitioned into ORF- and nonORF-containing sequences using ESTScan [28]. The ORF-containing transcripts were annotated for protein domains and functional sites by matching them against the PFAM, PROSITE and PRINTS databases [41-43] using hmmpfam, a program within the HMMER package that uses hidden Markov models to do sensitive searching of a protein database [44]. The protein domains were mapped to gene ontology categories using GO tables [45].

### Mapping of common carp transcripts to the zebrafish genome

In order to further categorize the common carp transcripts they were searched against the zebrafish genome assembly (version 5). The possibility of multigene families within EST clusters allow for common carp clusters to map to multiple zebrafish genomic locations. A single high quality zebrafish genomic location was identified for each mapped common cluster in order to screen for novel genes and potential alternative splice variants.

Transcripts that map to the zebrafish genome with BLAST E-value of at least 1e-04 where passed through a set of stringent filters as defined in Additional File 8: Figure S1 in order to identify a single zebrafish genomic location for each of the mapped common carp clusters. The best zebrafish chromosome location for each EST in a common carp cluster was considered: the zebrafish chromosome locus shared by all ESTs within a cluster was chosen as the mapped genomic locus for the corresponding common carp cluster. EST clusters that represented best hits to different chromosome locations for constituent ESTs were screened for a common zebrafish chromosome hit by considering the top five best hits for each EST in a cluster. A common zebrafish chromosome hit identified in the top five best hits was assigned as the unique map location for the common carp cluster. Mapped common carp clusters were not considered if there was not at least one zebrafish chromosome hit shared among all the ESTs in a cluster. All common carp transcripts that passed these filtering criteria were aligned to the specific segment of the overlapping zebrafish genome using EST2GENOME [31].

Exon-intron boundaries were extracted from the EST2GENOME results and served as a DAS track on the ensembl browser [30].

Genome-aligned common carp ESTs were classified according to one of five criteria that were satisfied on the plus or minus strand. Common carp ESTs overlapped (1) zebrafish coding regions: exons corresponding to an Ensembl gene or zebrafish EST; or (2) zebrafish introns: the entire genome-aligned common carp EST was contained within the intronic region(s) of a zebrafish gene; or (3) non-zebrafish exons: common carp ESTs mapped to regions of the zebrafish genome that overlapped non-zebrafish cDNA; (4) non-zebrafish introns: the non-coding portions of cDNA or proteins aligned to the zebrafish genome; (5) intergenic: regions of the zebrafish genome void of any annotations; (6) *ab initio *predictions: common carp ESTs mapped to regions of the zebrafish genome with an *in silico *gene prediction only.

### Comparing testis derived common carp sequences with zebrafish and fathead minnow EST data

Testis-only transcripts for common carp were defined as clusters or singletons represented by ESTs obtained exclusively from common carp testis cDNA libraries. Gonad derived genes for zebrafish were sampled from the UniGene zebrafish collection (build 91) where UniGene clusters contained ESTs that were sampled from zebrafish testis or ovary cDNA libraries. Common carp testis-derived transcripts were searched against the zebrafish gonad-derived UniGene dataset using BLASTN with (i) an E-value < 1e-04; and (ii) sequence overlap where 40% of the query sequence overlapped the matching database sequence. The common carp transcripts without identity to zebrafish gonad derived sequences were searched against the remainder of the zebrafish UniGene build 91 using an E-value < 1e-04 but without the requirement for 50% of the query sequence overlapping the database sequence. This relaxed criteria resulted in the identification of fewer common carp ESTs without homologous zebrafish ESTs in UniGene (build 91). However, these common carp ESTs provide a minimum dataset of testis-derived sequences not sampled by the zebrafish EST collection. The resulting "unique" common carp transcripts were searched against the fathead minnow UniGene (build 91) EST data using the same criteria as used for zebrafish.

### Acquisition of sequence data for common carp and zebrafish orthologs and paralogs

A total 652 common carp mRNA sequences were downloaded from GenBank. About 292 mRNAs represented partial mRNA sequences and were removed. The sequences corresponding to the remaining 360 mRNA records in GenBank were searched against NCBI's non-redundant database using protein-protein BLAST (blastp; [46]). The BLAST results were filtered for a significant sequence match to zebrafish (E-value < 1e-05) and matching zebrafish mRNAs that were partial sequences was filtered. The remaining 183 common carp and zebrafish homologous pairs were screened manually for orthologous relationships using cross-linked information including publications, curated annotations and filtering for redundant GenBank records. Eventually 120 pairs of orthologous genes were selected for sequence comparison between coding and non-coding regions (Additional File 7: Table S7) and a subset containing 48 pairs, plus six additional paralogs (all with at least 50 nucleotides upstream of the first protein coding exon) was used for motif searches (highlighted sequences in Additional File 7: Table S7).

First we analyzed the sequence similarity among the coding regions and the UTRs for the orthologous gene set. At the nucleotide level, sequence conservation was observed more often in the CDS regions, followed by the 5' UTR and 3' UTR regions, respectively (Additional File 10: Figure S3). Specifically, 75% of the orthologous pairs are captured when we set a sequence identity threshold of 80% at the CDS and protein levels. In comparison, only 25% of the 5' UTR sequences are captured under the same conditions (Additional File 10: Fig. S3). The threshold of 80% sequence identity was implemented for subsequent BLAST searches of common carp ESTs against the zebrafish genome assembly.

### *Ab-initio *motif identification, motif arrangement and 5'UTR sequence similarity

For the identification of motifs in 5'UTR regions, we compared the efficiency of Dragon Motif Builder system [47] with a local alignment method, ClustalW [48]. With the Dragon Motif Builder we searched for any motif with the length between 6 bp and 10 bp, used the matrix score threshold of 0.9, and searched for up to 10 motifs in the two sequences of a given orthologous pair (in some cases the 5'UTR regions were very short providing not enough sequence length to harbour all 10 motifs). For ClustalW the common motifs were manually identified and restricted to the same criteria as those used by Dragon Motif Builder (motifs of length 6 to 10 bp). A significant difference was observed showing that ClustalW was not able to identify sufficient similarity between the ortholog sequences in the 5'UTR regions (Additional File 9: Figure S2) as the segments that contain similar arrangement of common motifs between the two species were not residing at similar genomic locations.

Once the motifs were identified, we analyzed the motif arrangements. We selected the group of motifs that contained the largest number of common motifs, but retained the same positional arrangement in the two species (see Fig. 4 for a specific example). Thus each of the ortholog pairs was screened for such a representative motif arrangement. We used the number of motifs in the representative arrangements as a possible measure of similarity between the 5'UTR regions. In most cases, the regions where this arrangement had been spotted, was found at significantly different distances from the starting codon.

## Authors' contributions

AC had designed the bioinformatics pathway, performed most computational analyses, generated most tables and figures and took part in the writing of the manuscript. RB has contributed to the experimental design, constructed the full-length libraries and took part in the writing of the manuscript. HS has participated in the bioinformatics analysis of the data and in the maintenance of the cyprinid EST database of TLL. HK has supervised the generation of YY androgenic common carp line, generated the monosex populations, isolated the testis samples from them and contributed to the experimental design. LO initiated the project on comparative analysis of cyprinid ESTs, contributed to the experimental design and took part in the writing of the manuscript.

## Supplementary Material

Additional File 1 The description of common carp cDNA libraries analyzed in this study. Details include tissue, developmental stage and source of cDNA libraries. Click here for file

Additional File 2 Distribution of functional categories identified in the partial transcriptome of common carp. Click here for file

Additional File 8 Protocol to map common carp transcripts to the zebrafish genome assembly (v5). The flow chart depicts the pipeline implemented for mapping common carp transcripts to the zebrafish genome. Filter criteria are denoted in the decision tree. Total number of clusters and singletons are indicated in square brackets. Click here for file

Additional File 3 Classification of 91 common carp ESTs that map to intergenic, intronic, *ab initio *predictions and non-zebrafish supported annotations. Click here for file

Additional File 4 List of hyperlinks to potential common carp splice variants. Overlapping common carp and zebrafish transcripts are presented on a gbrowse viewer to highlight the missing exons in one of the two species. Click here for file

Additional File 5 List of 974 testis-only transcripts that do not overlap any of the zebrafish and fathead minnow ESTs. Click here for file

Additional File 6 List of 474 testis-derived clusters that show sequence identity to 474 zebrafish and 75 Fathead minnow UniGene clusters. Testis-expression information was added to the adult-stage zebrafish expression data. Click here for file

Additional File 7 Homologous gene pairs identified through manual curation of common carp and zebrafish genes. The table includes DNA and protein accession numbers and corresponding gene descriptions. Genes in rows highlighted with yellow contain 5' UTR sequence (>= 50 bp) and were used in UTR analysis. Click here for file

Additional File 9 Distribution of orthologous clusters with given number of common motifs using Dragon motif builder (blue bars) and CLUSTALW (red bars). Click here for file

Additional File 10 Percent sequence identity between common carp and zebrafish orthologous proteins, CDS, 5' UTR and 3' UTR regions. Click here for file
