# Supplementary material for: Comparative genomics in cyprinids: common carp ESTs help the annotation of the zebrafish genome
Source: BMC Bioinformatics. 2006 Dec 18;7(Suppl 5):S2. doi: 10.1186/1471-2105-7-S5-S2 (PMC1764476; doi:10.1186/1471-2105-7-S5-S2)
Supplement: Additional File 1 — The description of common carp cDNA libraries analyzed in this study. Details include tissue, developmental stage and source of cDNA libraries. [file 1471-2105-7-S5-S2-S1.doc]

Table S1: Description of the common carp cDNA clone sets analyzed

| Tissue | Library Type | Final set | Source |
| --- | --- | --- | --- |
| Testis – 60 dpf | Full-length | 1,644 | TLL |
| Testis – 80 dpf | Full-length | 1,255 | TLL |
| Testis – 100 dpf | Full-length | 730 | TLL |
| Testis – 70 dpf | Subtracted | 677 | TLL |
| Testis – 100 dpf | Subtracted | 767 | TLL |
| Liver, skeletal white muscle, cardiac muscle, kidney, brain, gill, intestinal mucosa | Subtracted | 527 | CarpBASE ESTs |
| skeletal white muscle, cardiac muscle, kidney, brain, gill, intestinal mucosa | Subtracted | 4165 | CarpBASE ESTs |
| Liver | Subtracted | 3,210 | CarpBASE ESTs |
| Muscle | Subtracted | 502 | CarpBASE ESTs |
|  | Unknown | 5 | CarpBASE mRNA |
| Barbell | Subtracted | 227 | GenBank ESTs |
| Head kidney (untreated and treated) | Full-length | 679 | GenBank ESTs |
| Leukocytes | Subtracted | 118 | GenBank ESTs |
| Peritoneal cells | Subtracted | 198 | GenBank ESTs |
| Mixed | Unknown | 652 | GenBank mRNA |
