# Supplementary material for: Comparative genomics in cyprinids: common carp ESTs help the annotation of the zebrafish genome
Source: BMC Bioinformatics. 2006 Dec 18;7(Suppl 5):S2. doi: 10.1186/1471-2105-7-S5-S2 (PMC1764476; doi:10.1186/1471-2105-7-S5-S2)
Supplement: Additional File 2 — Distribution of functional categories identified in the partial transcriptome of common carp. [file 1471-2105-7-S5-S2-S2.doc]

Table S2. Distribution of functional categories identified in the partial transcriptome of common carp.

| **Frequency** | **Gene Ontology ID** | **Gene ontology description** |
| --- | --- | --- |
| 147 | GO:0005634 | nucleus |
| 145 | GO:0016020 | membrane |
| 143 | GO:0005524 | ATP binding |
| 127 | GO:0005622 | intracellular |
| 124 | GO:0006118 | electron transport |
| 114 | GO:0003677 | DNA binding |
| 104 | GO:0016021 | integral to membrane |
| 97 | GO:0003735 | structural constituent of ribosome |
| 97 | GO:0006412 | protein biosynthesis |
| 97 | GO:0005840 | ribosome |
| 96 | GO:0006508 | proteolysis and peptidolysis |
| 93 | GO:0006355 | regulation of transcription, DNA-dependent |
| 91 | GO:0005509 | calcium ion binding |
| 88 | GO:0006468 | protein amino acid phosphorylation |
| 76 | GO:0006810 | transport |
| 64 | GO:0005576 | extracellular |
| 63 | GO:0005489 | electron transporter activity |
| 62 | GO:0003676 | nucleic acid binding |
| 58 | GO:0008152 | metabolism |
| 52 | GO:0007186 | G-protein coupled receptor protein signaling pathway |
| 51 | GO:0008270 | zinc ion binding |
| 47 | GO:0004672 | protein kinase activity |
| 46 | GO:0005525 | GTP binding |
| 45 | GO:0001584 | rhodopsin-like receptor activity |
| 44 | GO:0016491 | oxidoreductase activity |
| 44 | GO:0003700 | transcription factor activity |
| 40 | GO:0003824 | catalytic activity |
| 37 | GO:0005215 | transporter activity |
| 36 | GO:0020037 | heme binding |
| 35 | GO:0005198 | structural molecule activity |
| 34 | GO:0016469 | proton-transporting two-sector ATPase complex |
| 33 | GO:0015986 | ATP synthesis coupled proton transport |
| 33 | GO:0046933 | hydrogen-transporting ATP synthase activity, rotational mechanism |
| 33 | GO:0046961 | hydrogen-transporting ATPase activity, rotational mechanism |
| 28 | GO:0006464 | protein modification |
| 26 | GO:0005488 | binding |
| 26 | GO:0006512 | ubiquitin cycle |
| 26 | GO:0006511 | ubiquitin-dependent protein catabolism |
| 26 | GO:0006096 | glycolysis |
| 24 | GO:0004295 | trypsin activity |
| 24 | GO:0006457 | protein folding |
| 24 | GO:0007242 | intracellular signaling cascade |
| 23 | GO:0004674 | protein serine/threonine kinase activity |
| 23 | GO:0006955 | immune response |
| 22 | GO:0004129 | cytochrome-c oxidase activity |
| 22 | GO:0007264 | small GTPase mediated signal transduction |
| 21 | GO:0004840 | ubiquitin conjugating enzyme activity |
| 21 | GO:0005179 | hormone activity |
| 20 | GO:0004175 | endopeptidase activity |
| 20 | GO:0005839 | proteasome core complex (sensu Eukarya) |
| 19 | GO:0005554 | molecular_function unknown |
| 19 | GO:0003723 | RNA binding |
| 19 | GO:0005975 | carbohydrate metabolism |
| 19 | GO:0007165 | signal transduction |
| 18 | GO:0003779 | actin binding |
| 18 | GO:0005529 | sugar binding |
| 17 | GO:0005515 | protein binding |
| 17 | GO:0004871 | signal transducer activity |
| 17 | GO:0006334 | nucleosome assembly |
| 17 | GO:0007018 | microtubule-based movement |
| 17 | GO:0005874 | microtubule |
| 16 | GO:0004197 | cysteine-type endopeptidase activity |
| 16 | GO:0006470 | protein amino acid dephosphorylation |
| 16 | GO:0004867 | serine-type endopeptidase inhibitor activity |
| 16 | GO:0006886 | intracellular protein transport |
| 15 | GO:0000786 | nucleosome |
| 14 | GO:0005856 | cytoskeleton |
| 14 | GO:0003743 | translation initiation factor activity |
| 14 | GO:0008009 | chemokine activity |
| 13 | GO:0005737 | cytoplasm |
| 13 | GO:0008289 | lipid binding |
| 11 | GO:0007155 | cell adhesion |
| 11 | GO:0006413 | translational initiation |
| 11 | GO:0042981 | regulation of apoptosis |
| 11 | GO:0008248 | pre-mRNA splicing factor activity |
| 11 | GO:0005732 | small nucleolar ribonucleoprotein complex |
| 11 | GO:0003995 | acyl-CoA dehydrogenase activity |
| 10 | GO:0016787 | hydrolase activity |
| 10 | GO:0003754 | chaperone activity |
| 10 | GO:0004866 | endopeptidase inhibitor activity |
| 10 | GO:0008083 | growth factor activity |
| 10 | GO:0004222 | metalloendopeptidase activity |
| 10 | GO:0004182 | carboxypeptidase A activity |
| 10 | GO:0005200 | structural constituent of cytoskeleton |
| 9 | GO:0016301 | kinase activity |
| 9 | GO:0046872 | metal ion binding |
| 9 | GO:0005783 | endoplasmic reticulum |
| 9 | GO:0015671 | oxygen transport |
| 9 | GO:0005578 | extracellular matrix |
| 9 | GO:0003774 | motor activity |
| 9 | GO:0005507 | copper ion binding |
| 9 | GO:0007001 | chromosome organization and biogenesis (sensu Eukarya) |
| 8 | GO:0006414 | translational elongation |
| 8 | GO:0004872 | receptor activity |
| 8 | GO:0004869 | cysteine protease inhibitor activity |
| 8 | GO:0005544 | calcium-dependent phospholipid binding |
| 8 | GO:0004721 | phosphoprotein phosphatase activity |
| 8 | GO:0004713 | protein-tyrosine kinase activity |
| 8 | GO:0004009 | ATP-binding cassette (ABC) transporter activity |
| 7 | GO:0016772 | transferase activity, transferring phosphorus-containing groups |
| 7 | GO:0006812 | cation transport |
| 7 | GO:0005740 | mitochondrial membrane |
| 7 | GO:0007010 | cytoskeleton organization and biogenesis |
| 7 | GO:0007160 | cell-matrix adhesion |
| 7 | GO:0006350 | transcription |
| 7 | GO:0008121 | ubiquinol-cytochrome-c reductase activity |
| 7 | GO:0006629 | lipid metabolism |
| 7 | GO:0006281 | DNA repair |
| 7 | GO:0008237 | metallopeptidase activity |
| 7 | GO:0006811 | ion transport |
| 7 | GO:0004714 | transmembrane receptor protein tyrosine kinase activity |
| 6 | GO:0004725 | protein tyrosine phosphatase activity |
| 6 | GO:0006869 | lipid transport |
| 6 | GO:0006979 | response to oxidative stress |
| 6 | GO:0003899 | DNA-directed RNA polymerase activity |
| 6 | GO:0006605 | protein targeting |
| 6 | GO:0008170 | N-methyltransferase activity |
| 6 | GO:0006306 | DNA methylation |
| 6 | GO:0006260 | DNA replication |
| 6 | GO:0007601 | visual perception |
| 6 | GO:0009058 | biosynthesis |
| 5 | GO:0008146 | sulfotransferase activity |
| 5 | GO:0004221 | ubiquitin thiolesterase activity |
| 5 | GO:0004386 | helicase activity |
| 5 | GO:0008565 | protein transporter activity |
| 5 | GO:0003746 | translation elongation factor activity |
| 5 | GO:0019904 | protein domain specific binding |
| 5 | GO:0042157 | lipoprotein metabolism |
| 5 | GO:0008199 | ferric iron binding |
| 5 | GO:0006879 | iron ion homeostasis |
| 5 | GO:0006099 | tricarboxylic acid cycle |
| 5 | GO:0005743 | mitochondrial inner membrane |
| 5 | GO:0004743 | pyruvate kinase activity |
| 5 | GO:0000074 | regulation of cell cycle |
| 5 | GO:0008234 | cysteine-type peptidase activity |
| 5 | GO:0007275 | development |
| 5 | GO:0008305 | integrin complex |
| 5 | GO:0005739 | mitochondrion |
| 5 | GO:0015629 | actin cytoskeleton |
| 5 | GO:0004190 | aspartic-type endopeptidase activity |
| 5 | GO:0006310 | DNA recombination |
| 5 | GO:0004623 | phospholipase A2 activity |
| 5 | GO:0016042 | lipid catabolism |
| 5 | GO:0000160 | two-component signal transduction system (phosphorelay) |
| 5 | GO:0008233 | peptidase activity |
| 5 | GO:0004707 | MAP kinase activity |
| 5 | GO:0005884 | actin filament |
| 5 | GO:0030001 | metal ion transport |
| 5 | GO:0003707 | steroid hormone receptor activity |
| 5 | GO:0006813 | potassium ion transport |
| 5 | GO:0004812 | tRNA ligase activity |
| 5 | GO:0006418 | tRNA aminoacylation for protein translation |
| 5 | GO:0007169 | transmembrane receptor protein tyrosine kinase signaling pathway |
| 4 | GO:0004930 | G-protein coupled receptor activity |
| 4 | GO:0004857 | enzyme inhibitor activity |
| 4 | GO:0008137 | NADH dehydrogenase (ubiquinone) activity |
| 4 | GO:0006120 | mitochondrial electron transport, NADH to ubiquinone |
| 4 | GO:0015031 | protein transport |
| 4 | GO:0030484 | muscle fiber |
| 4 | GO:0015662 | ATPase activity, coupled to transmembrane movement of ions, phosphorylative mechanism |
| 4 | GO:0005834 | heterotrimeric G-protein complex |
| 4 | GO:0004602 | glutathione peroxidase activity |
| 4 | GO:0000785 | chromatin |
| 4 | GO:0005498 | sterol carrier activity |
| 4 | GO:0007154 | cell communication |
| 4 | GO:0007582 | physiological process |
| 4 | GO:0008094 | DNA-dependent ATPase activity |
| 4 | GO:0008308 | voltage-dependent ion-selective channel activity |
| 4 | GO:0006820 | anion transport |
| 4 | GO:0005741 | mitochondrial outer membrane |
| 4 | GO:0006122 | mitochondrial electron transport, ubiquinol to cytochrome c |
| 4 | GO:0008191 | metalloendopeptidase inhibitor activity |
| 4 | GO:0005875 | microtubule associated complex |
| 4 | GO:0005786 | signal recognition particle |
| 4 | GO:0003872 | 6-phosphofructokinase activity |
| 4 | GO:0005945 | 6-phosphofructokinase complex |
| 4 | GO:0005328 | neurotransmitter:sodium symporter activity |
| 4 | GO:0006836 | neurotransmitter transport |
| 4 | GO:0005887 | integral to plasma membrane |
| 4 | GO:0007156 | homophilic cell adhesion |
| 4 | GO:0005085 | guanyl-nucleotide exchange factor activity |
| 4 | GO:0003684 | damaged DNA binding |
| 4 | GO:0005923 | tight junction |
| 4 | GO:0008026 | ATP-dependent helicase activity |
| 4 | GO:0005216 | ion channel activity |
| 4 | GO:0008415 | acyltransferase activity |
| 3 | GO:0006352 | transcription initiation |
| 3 | GO:0004194 | pepsin A activity |
| 3 | GO:0004332 | fructose-bisphosphate aldolase activity |
| 3 | GO:0008190 | eukaryotic initiation factor 4E binding |
| 3 | GO:0045947 | negative regulation of translational initiation |
| 3 | GO:0005506 | iron ion binding |
| 3 | GO:0006520 | amino acid metabolism |
| 3 | GO:0006754 | ATP biosynthesis |
| 3 | GO:0003713 | transcription coactivator activity |
| 3 | GO:0016758 | transferase activity, transferring hexosyl groups |
| 3 | GO:0004634 | phosphopyruvate hydratase activity |
| 3 | GO:0000015 | phosphopyruvate hydratase complex |
| 3 | GO:0005794 | Golgi apparatus |
| 3 | GO:0016651 | oxidoreductase activity, acting on NADH or NADPH |
| 3 | GO:0016868 | intramolecular transferase activity, phosphotransferases |
| 3 | GO:0016616 | oxidoreductase activity, acting on the CH-OH group of donors, NAD or NADP as acceptor |
| 3 | GO:0006564 | L-serine biosynthesis |
| 3 | GO:0004550 | nucleoside-diphosphate kinase activity |
| 3 | GO:0006183 | GTP biosynthesis |
| 3 | GO:0006228 | UTP biosynthesis |
| 3 | GO:0006241 | CTP biosynthesis |
| 3 | GO:0004365 | glyceraldehyde-3-phosphate dehydrogenase (phosphorylating) activity |
| 3 | GO:0005746 | mitochondrial electron transport chain |
| 3 | GO:0004024 | alcohol dehydrogenase activity, zinc-dependent |
| 3 | GO:0005882 | intermediate filament |
| 3 | GO:0006606 | protein-nucleus import |
| 3 | GO:0006730 | one-carbon compound metabolism |
| 3 | GO:0005149 | interleukin-1 receptor binding |
| 3 | GO:0016459 | myosin |
| 3 | GO:0004396 | hexokinase activity |
| 3 | GO:0040007 | growth |
| 3 | GO:0005669 | transcription factor TFIID complex |
| 3 | GO:0006631 | fatty acid metabolism |
| 3 | GO:0015450 | protein translocase activity |
| 3 | GO:0005201 | extracellular matrix structural constituent |
| 3 | GO:0005581 | collagen |
| 3 | GO:0004645 | phosphorylase activity |
| 3 | GO:0005386 | carrier activity |
| 3 | GO:0003777 | microtubule motor activity |
| 3 | GO:0007017 | microtubule-based process |
| 3 | GO:0016615 | malate dehydrogenase activity |
| 3 | GO:0004459 | L-lactate dehydrogenase activity |
| 3 | GO:0004842 | ubiquitin-protein ligase activity |
| 3 | GO:0016462 | pyrophosphatase activity |
| 3 | GO:0007049 | cell cycle |
| 3 | GO:0005351 | sugar porter activity |
| 3 | GO:0009401 | phosphoenolpyruvate-dependent sugar phosphotransferase system |
| 3 | GO:0006817 | phosphate transport |
| 3 | GO:0008483 | transaminase activity |
| 3 | GO:0005249 | voltage-gated potassium channel activity |
| 3 | GO:0008076 | voltage-gated potassium channel complex |
| 3 | GO:0016740 | transferase activity |
| 3 | GO:0045941 | positive regulation of transcription |
| 3 | GO:0030125 | clathrin vesicle coat |
| 3 | GO:0030154 | cell differentiation |
| 3 | GO:0008538 | proteasome activator activity |
| 3 | GO:0008537 | proteasome activator complex |
| 3 | GO:0003702 | RNA polymerase II transcription factor activity |
| 3 | GO:0006367 | transcription initiation from Pol II promoter |
| 3 | GO:0005667 | transcription factor complex |
| 3 | GO:0004556 | alpha-amylase activity |
| 3 | GO:0005230 | extracellular ligand-gated ion channel activity |
| 3 | GO:0006446 | regulation of translational initiation |
| 3 | GO:0008324 | cation transporter activity |
| 3 | GO:0007517 | muscle development |
| 3 | GO:0004601 | peroxidase activity |
| 2 | GO:0016012 | sarcoglycan complex |
| 2 | GO:0030041 | actin filament polymerization |
| 2 | GO:0004470 | malic enzyme activity |
| 2 | GO:0006108 | malate metabolism |
| 2 | GO:0006003 | fructose 2,6-bisphosphate metabolism |
| 2 | GO:0005853 | eukaryotic translation elongation factor 1 complex |
| 2 | GO:0004497 | monooxygenase activity |
| 2 | GO:0004800 | thyroxine 5'-deiodinase activity |
| 2 | GO:0008073 | ornithine decarboxylase inhibitor activity |
| 2 | GO:0004833 | tryptophan 2,3-dioxygenase activity |
| 2 | GO:0006568 | tryptophan metabolism |
| 2 | GO:0016829 | lyase activity |
| 2 | GO:0006801 | superoxide metabolism |
| 2 | GO:0030036 | actin cytoskeleton organization and biogenesis |
| 2 | GO:0008290 | F-actin capping protein complex |
| 2 | GO:0005777 | peroxisome |
| 2 | GO:0006826 | iron ion transport |
| 2 | GO:0004807 | triose-phosphate isomerase activity |
| 2 | GO:0016820 | hydrolase activity, acting on acid anhydrides, catalyzing transmembrane movement of substances |
| 2 | GO:0005478 | intracellular transporter activity |
| 2 | GO:0000062 | acyl-CoA binding |
| 2 | GO:0003682 | chromatin binding |
| 2 | GO:0006333 | chromatin assembly/disassembly |
| 2 | GO:0008430 | selenium binding |
| 2 | GO:0018149 | peptide cross-linking |
| 2 | GO:0003924 | GTPase activity |
| 2 | GO:0046785 | microtubule polymerization |
| 2 | GO:0045298 | tubulin |
| 2 | GO:0006094 | gluconeogenesis |
| 2 | GO:0015285 | connexon channel activity |
| 2 | GO:0005922 | connexon complex |
| 2 | GO:0045978 | negative regulation of nucleoside metabolism |
| 2 | GO:0005778 | peroxisomal membrane |
| 2 | GO:0006825 | copper ion transport |
| 2 | GO:0043087 | regulation of GTPase activity |
| 2 | GO:0016853 | isomerase activity |
| 2 | GO:0005496 | steroid binding |
| 2 | GO:0004345 | glucose-6-phosphate 1-dehydrogenase activity |
| 2 | GO:0006006 | glucose metabolism |
| 2 | GO:0030833 | regulation of actin filament polymerization |
| 2 | GO:0009755 | hormone mediated signaling |
| 2 | GO:0005516 | calmodulin binding |
| 2 | GO:0004462 | lactoylglutathione lyase activity |
| 2 | GO:0004289 | subtilase activity |
| 2 | GO:0004896 | hematopoietin/interferon-class (D200-domain) cytokine receptor activity |
| 2 | GO:0004367 | glycerol-3-phosphate dehydrogenase (NAD+) activity |
| 2 | GO:0006072 | glycerol-3-phosphate metabolism |
| 2 | GO:0009331 | glycerol-3-phosphate dehydrogenase complex |
| 2 | GO:0045285 | ubiquinol-cytochrome-c reductase complex |
| 2 | GO:0006888 | ER to Golgi transport |
| 2 | GO:0016773 | phosphotransferase activity, alcohol group as acceptor |
| 2 | GO:0005635 | nuclear membrane |
| 2 | GO:0008601 | protein phosphatase type 2A regulator activity |
| 2 | GO:0000159 | protein phosphatase type 2A complex |
| 2 | GO:0004372 | glycine hydroxymethyltransferase activity |
| 2 | GO:0006544 | glycine metabolism |
| 2 | GO:0006563 | L-serine metabolism |
| 2 | GO:0016814 | hydrolase activity, acting on carbon-nitrogen (but not peptide) bonds, in cyclic amidines |
| 2 | GO:0004512 | inositol-3-phosphate synthase activity |
| 2 | GO:0006021 | myo-inositol biosynthesis |
| 2 | GO:0008654 | phospholipid biosynthesis |
| 2 | GO:0008373 | sialyltransferase activity |
| 2 | GO:0006486 | protein amino acid glycosylation |
| 2 | GO:0003993 | acid phosphatase activity |
| 2 | GO:0016810 | hydrolase activity, acting on carbon-nitrogen (but not peptide) bonds |
| 2 | GO:0006807 | nitrogen metabolism |
| 2 | GO:0004180 | carboxypeptidase activity |
| 2 | GO:0046873 | metal ion transporter activity |
| 2 | GO:0004435 | phosphoinositide phospholipase C activity |
| 2 | GO:0006816 | calcium ion transport |
| 2 | GO:0006626 | protein-mitochondrial targeting |
| 2 | GO:0004392 | heme oxygenase (decyclizing) activity |
| 2 | GO:0006788 | heme oxidation |
| 2 | GO:0045211 | postsynaptic membrane |
| 2 | GO:0004879 | ligand-dependent nuclear receptor activity |
| 2 | GO:0005006 | epidermal growth factor receptor activity |
| 2 | GO:0008605 | protein kinase CK2 regulator activity |
| 2 | GO:0005956 | protein kinase CK2 complex |
| 2 | GO:0050825 | ice binding |
| 2 | GO:0042309 | homoiothermy |
| 2 | GO:0050826 | response to freezing |
| 2 | GO:0008235 | metalloexopeptidase activity |
| 2 | GO:0003725 | double-stranded RNA binding |
| 2 | GO:0030693 | caspase activity |
| 2 | GO:0004553 | hydrolase activity, hydrolyzing O-glycosyl compounds |
| 1 | GO:0016308 | 1-phosphatidylinositol-4-phosphate 5-kinase activity |
| 1 | GO:0016310 | phosphorylation |
| 1 | GO:0000059 | protein-nucleus import, docking |
| 1 | GO:0005643 | nuclear pore |
| 1 | GO:0005759 | mitochondrial matrix |
| 1 | GO:0005523 | tropomyosin binding |
| 1 | GO:0008746 | NAD(P) transhydrogenase activity |
| 1 | GO:0009072 | aromatic amino acid family metabolism |
| 1 | GO:0017072 | tubulin-specific chaperone activity |
| 1 | GO:0007022 | chaperonin-mediated tubulin folding |
| 1 | GO:0008061 | chitin binding |
| 1 | GO:0006030 | chitin metabolism |
| 1 | GO:0016051 | carbohydrate biosynthesis |
| 1 | GO:0004785 | copper, zinc superoxide dismutase activity |
| 1 | GO:0003997 | acyl-CoA oxidase activity |
| 1 | GO:0006635 | fatty acid beta-oxidation |
| 1 | GO:0016228 | aldolase activity |
| 1 | GO:0004798 | thymidylate kinase activity |
| 1 | GO:0006233 | dTDP biosynthesis |
| 1 | GO:0006235 | dTTP biosynthesis |
| 1 | GO:0004177 | aminopeptidase activity |
| 1 | GO:0016599 | caveolar membrane |
| 1 | GO:0004522 | pancreatic ribonuclease activity |
| 1 | GO:0004846 | urate oxidase activity |
| 1 | GO:0006144 | purine base metabolism |
| 1 | GO:0008717 | D-alanyl-D-alanine endopeptidase activity |
| 1 | GO:0005838 | proteasome regulatory particle (sensu Eukarya) |
| 1 | GO:0004411 | homogentisate 1,2-dioxygenase activity |
| 1 | GO:0006559 | phenylalanine catabolism |
| 1 | GO:0006570 | tyrosine metabolism |
| 1 | GO:0009607 | response to biotic stimulus |
| 1 | GO:0004089 | carbonate dehydratase activity |
| 1 | GO:0030163 | protein catabolism |
| 1 | GO:0005164 | tumor necrosis factor receptor binding |
| 1 | GO:0004611 | phosphoenolpyruvate carboxykinase activity |
| 1 | GO:0005319 | lipid transporter activity |
| 1 | GO:0004516 | nicotinate phosphoribosyltransferase activity |
| 1 | GO:0019363 | pyridine nucleotide biosynthesis |
| 1 | GO:0008320 | protein carrier activity |
| 1 | GO:0006954 | inflammatory response |
| 1 | GO:0008533 | astacin activity |
| 1 | GO:0046080 | dUTP metabolism |
| 1 | GO:0006270 | DNA replication initiation |
| 1 | GO:0009003 | signal peptidase activity |
| 1 | GO:0015035 | protein disulfide oxidoreductase activity |
| 1 | GO:0030288 | periplasmic space (sensu Gram-negative Bacteria) |
| 1 | GO:0006364 | rRNA processing |
| 1 | GO:0004146 | dihydrofolate reductase activity |
| 1 | GO:0006545 | glycine biosynthesis |
| 1 | GO:0009165 | nucleotide biosynthesis |
| 1 | GO:0005744 | mitochondrial inner membrane presequence translocase complex |
| 1 | GO:0016531 | copper chaperone activity |
| 1 | GO:0005758 | mitochondrial intermembrane space |
| 1 | GO:0030528 | transcription regulator activity |
| 1 | GO:0004013 | adenosylhomocysteinase activity |
| 1 | GO:0004359 | glutaminase activity |
| 1 | GO:0006541 | glutamine metabolism |
| 1 | GO:0007530 | sex determination |
| 1 | GO:0004784 | superoxide dismutase activity |
| 1 | GO:0007602 | phototransduction |
| 1 | GO:0009405 | pathogenesis |
| 1 | GO:0004019 | adenylosuccinate synthase activity |
| 1 | GO:0006164 | purine nucleotide biosynthesis |
| 1 | GO:0015008 | ubiquinol-cytochrome-c reductase complex (sensu Eukarya) |
| 1 | GO:0004722 | protein serine/threonine phosphatase activity |
| 1 | GO:0008287 | protein serine/threonine phosphatase complex |
| 1 | GO:0003910 | DNA ligase (ATP) activity |
| 1 | GO:0003884 | D-amino-acid oxidase activity |
| 1 | GO:0005044 | scavenger receptor activity |
| 1 | GO:0008318 | protein prenyltransferase activity |
| 1 | GO:0018346 | protein amino acid prenylation |
| 1 | GO:0006633 | fatty acid biosynthesis |
| 1 | GO:0004693 | cyclin-dependent protein kinase activity |
| 1 | GO:0042578 | phosphoric ester hydrolase activity |
| 1 | GO:0004096 | catalase activity |
| 1 | GO:0003918 | DNA topoisomerase (ATP-hydrolyzing) activity |
| 1 | GO:0006259 | DNA metabolism |
| 1 | GO:0006265 | DNA topological change |
| 1 | GO:0009765 | photosynthesis light harvesting |
| 1 | GO:0000175 | 3'-5'-exoribonuclease activity |
| 1 | GO:0006396 | RNA processing |
| 1 | GO:0005801 | Golgi cis-face |
| 1 | GO:0046983 | protein dimerization activity |
| 1 | GO:0006915 | apoptosis |
| 1 | GO:0006139 | nucleobase, nucleoside, nucleotide and nucleic acid metabolism |
| 1 | GO:0016055 | Wnt receptor signaling pathway |
| 1 | GO:0005158 | insulin receptor binding |
| 1 | GO:0004415 | hyalurononglucosaminidase activity |
| 1 | GO:0005315 | inorganic phosphate transporter activity |
| 1 | GO:0005099 | Ras GTPase activator activity |
| 1 | GO:0004428 | inositol/phosphatidylinositol kinase activity |
| 1 | GO:0004034 | aldose 1-epimerase activity |
| 1 | GO:0006012 | galactose metabolism |
| 1 | GO:0006397 | mRNA processing |
| 1 | GO:0008113 | protein-methionine-S-oxide reductase activity |
| 1 | GO:0048037 | cofactor binding |
| 1 | GO:0003726 | double-stranded RNA adenosine deaminase activity |
| 1 | GO:0004478 | methionine adenosyltransferase activity |
| 1 | GO:0016211 | ammonia ligase activity |
| 1 | GO:0016563 | transcriptional activator activity |
| 1 | GO:0019211 | phosphatase activator activity |
| 1 | GO:0008474 | palmitoyl-(protein) hydrolase activity |
| 1 | GO:0005764 | lysosome |
| 1 | GO:0005375 | copper ion transporter activity |
| 1 | GO:0005094 | Rho GDP-dissociation inhibitor activity |
| 1 | GO:0005674 | transcription factor TFIIF complex |
| 1 | GO:0005615 | extracellular space |
| 1 | GO:0004198 | calpain activity |
| 1 | GO:0016742 | hydroxymethyl-, formyl- and related transferase activity |
| 1 | GO:0004655 | porphobilinogen synthase activity |
| 1 | GO:0006783 | heme biosynthesis |
| 1 | GO:0004629 | phospholipase C activity |
| 1 | GO:0004347 | glucose-6-phosphate isomerase activity |
| 1 | GO:0006546 | glycine catabolism |
| 1 | GO:0005960 | glycine cleavage complex |
| 1 | GO:0005742 | mitochondrial outer membrane translocase complex |
| 1 | GO:0005391 | sodium/potassium-exchanging ATPase activity |
| 1 | GO:0006814 | sodium ion transport |
| 1 | GO:0006897 | endocytosis |
| 1 | GO:0008092 | cytoskeletal protein binding |
| 1 | GO:0030866 | cortical actin cytoskeleton organization and biogenesis |
| 1 | GO:0005245 | voltage-gated calcium channel activity |
| 1 | GO:0004618 | phosphoglycerate kinase activity |
| 1 | GO:0008151 | cell growth and/or maintenance |
| 1 | GO:0016600 | flotillin complex |
| 1 | GO:0004356 | glutamate-ammonia ligase activity |
| 1 | GO:0009399 | nitrogen fixation |
| 1 | GO:0004420 | hydroxymethylglutaryl-CoA reductase (NADPH) activity |
| 1 | GO:0016846 | carbon-sulfur lyase activity |
| 1 | GO:0030594 | neurotransmitter receptor activity |
| 1 | GO:0008067 | metabotropic glutamate, GABA-B-like receptor activity |
| 1 | GO:0003955 | NAD(P)H dehydrogenase (quinone) activity |
| 1 | GO:0016272 | prefoldin complex |
| 1 | GO:0006354 | RNA elongation |
| 1 | GO:0005514 | calcium ion storage activity |
| 1 | GO:0006342 | chromatin silencing |
| 1 | GO:0005677 | chromatin silencing complex |
| 1 | GO:0004329 | formate-tetrahydrofolate ligase activity |
| 1 | GO:0009396 | folic acid and derivative biosynthesis |
| 1 | GO:0000104 | succinate dehydrogenase activity |
| 1 | GO:0004185 | serine carboxypeptidase activity |
| 1 | GO:0004638 | phosphoribosylaminoimidazole carboxylase activity |
| 1 | GO:0006189 | 'de novo' IMP biosynthesis |
| 1 | GO:0009320 | phosphoribosylaminoimidazole carboxylase complex |
| 1 | GO:0045735 | nutrient reservoir activity |
| 1 | GO:0008745 | N-acetylmuramoyl-L-alanine amidase activity |
| 1 | GO:0009253 | peptidoglycan catabolism |
| 1 | GO:0004616 | phosphogluconate dehydrogenase (decarboxylating) activity |
| 1 | GO:0006098 | pentose-phosphate shunt |
| 1 | GO:0016290 | palmitoyl-CoA hydrolase activity |
| 1 | GO:0006298 | mismatch repair |
| 1 | GO:0004014 | adenosylmethionine decarboxylase activity |
| 1 | GO:0006597 | spermine biosynthesis |
| 1 | GO:0008295 | spermidine biosynthesis |
| 1 | GO:0006366 | transcription from Pol II promoter |
| 1 | GO:0005665 | DNA-directed RNA polymerase II, core complex |
| 1 | GO:0015036 | disulfide oxidoreductase activity |
| 1 | GO:0004748 | ribonucleoside-diphosphate reductase activity |
| 1 | GO:0005971 | ribonucleoside-diphosphate reductase complex |
| 1 | GO:0005247 | voltage-gated chloride channel activity |
| 1 | GO:0006821 | chloride transport |
| 1 | GO:0045449 | regulation of transcription |
| 1 | GO:0004517 | nitric-oxide synthase activity |
| 1 | GO:0006809 | nitric oxide biosynthesis |
| 1 | GO:0005184 | neuropeptide hormone activity |
| 1 | GO:0016757 | transferase activity, transferring glycosyl groups |
| 1 | GO:0007267 | cell-cell signaling |
| 1 | GO:0016539 | intein-mediated protein splicing |
| 1 | GO:0015321 | sodium-dependent phosphate transporter activity |
| 1 | GO:0004536 | deoxyribonuclease activity |
| 1 | GO:0006308 | DNA catabolism |
| 1 | GO:0005125 | cytokine activity |
| 1 | GO:0005185 | neurohypophyseal hormone activity |
| 1 | GO:0006953 | acute-phase response |
| 1 | GO:0004799 | thymidylate synthase activity |
| 1 | GO:0006231 | dTMP biosynthesis |
| 1 | GO:0008450 | O-sialoglycoprotein endopeptidase activity |
| 1 | GO:0030337 | DNA polymerase processivity factor activity |
| 1 | GO:0006275 | regulation of DNA replication |
| 1 | GO:0005660 | delta-DNA polymerase cofactor complex |
| 1 | GO:0004652 | polynucleotide adenylyltransferase activity |
| 1 | GO:0008138 | protein tyrosine/serine/threonine phosphatase activity |
| 1 | GO:0000049 | tRNA binding |
| 1 | GO:0005246 | calcium channel regulator activity |
| 1 | GO:0042030 | ATPase inhibitor activity |
| 1 | GO:0005851 | eukaryotic translation initiation factor 2B complex |
| 1 | GO:0003913 | DNA photolyase activity |
| 1 | GO:0004844 | uracil DNA N-glycosylase activity |
| 1 | GO:0004527 | exonuclease activity |
| 1 | GO:0008171 | O-methyltransferase activity |
| 1 | GO:0008159 | positive transcription elongation factor activity |
| 1 | GO:0008080 | N-acetyltransferase activity |
| 1 | GO:0005664 | nuclear origin of replication recognition complex |
| 1 | GO:0009374 | biotin binding |
| 1 | GO:0007046 | ribosome biogenesis |
| 1 | GO:0005242 | inward rectifier potassium channel activity |
| 1 | GO:0004419 | hydroxymethylglutaryl-CoA lyase activity |
| 1 | GO:0009116 | nucleoside metabolism |
| 1 | GO:0007283 | spermatogenesis |
| 1 | GO:0008442 | 3-hydroxyisobutyrate dehydrogenase activity |
| 1 | GO:0006573 | valine metabolism |
| 1 | GO:0015992 | proton transport |
| 1 | GO:0004839 | ubiquitin activating enzyme activity |
| 1 | GO:0006614 | SRP-dependent cotranslational membrane targeting |
| 1 | GO:0019239 | deaminase activity |
| 1 | GO:0009168 | purine ribonucleoside monophosphate biosynthesis |
| 1 | GO:0005344 | oxygen transporter activity |
| 1 | GO:0006952 | defense response |
| 1 | GO:0004519 | endonuclease activity |
| 1 | GO:0008202 | steroid metabolism |
