# Supplementary figures and images for: Comparative genomics in cyprinids: common carp ESTs help the annotation of the zebrafish genome
Source: BMC Bioinformatics. 2006 Dec 18;7(Suppl 5):S2. doi: 10.1186/1471-2105-7-S5-S2 (PMC1764476; doi:10.1186/1471-2105-7-S5-S2)

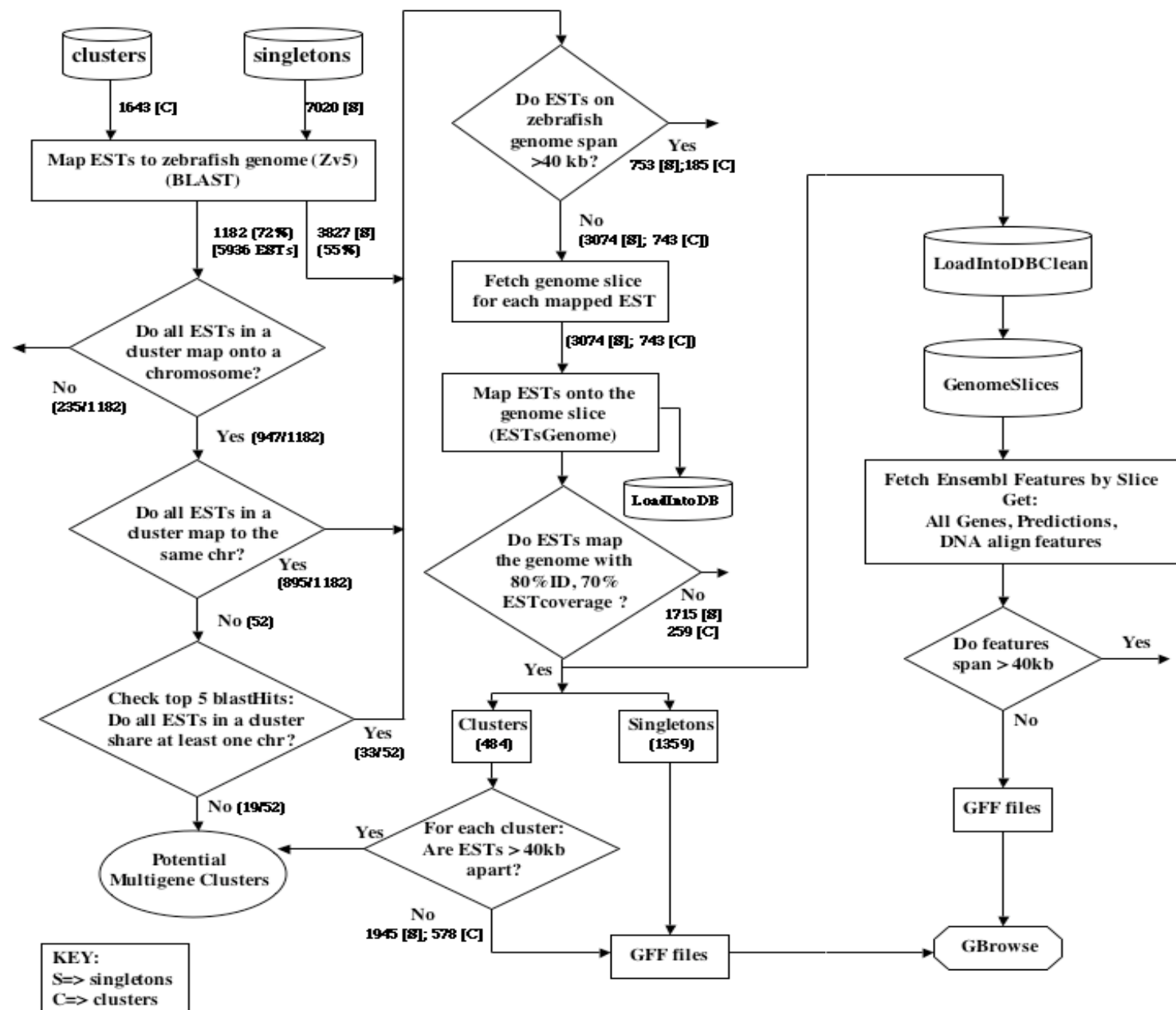

Supplement: Additional File 8 — Protocol to map common carp transcripts to the zebrafish genome assembly (v5). The flow chart depicts the pipeline implemented for mapping common carp transcripts to the zebrafish genome. Filter criteria are denoted in the decision tree. Total number of clusters and singletons are indicated in square brackets. [file 1471-2105-7-S5-S2-S8.pdf]

frequency of clusters (%)

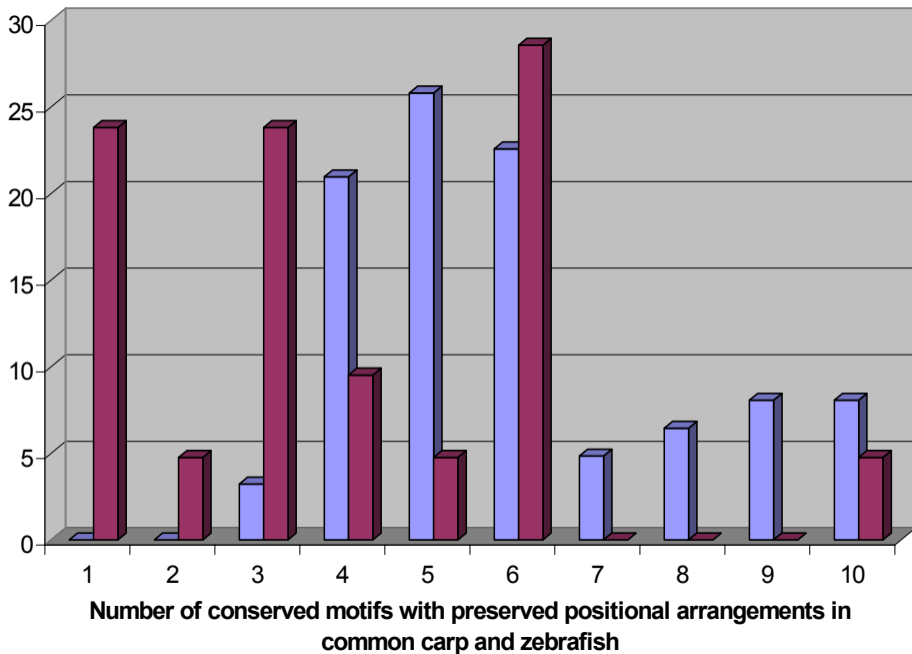

Supplement: Additional File 9 — Distribution of orthologous clusters with given number of common motifs using Dragon motif builder (blue bars) and CLUSTALW (red bars). [file 1471-2105-7-S5-S2-S9.pdf]

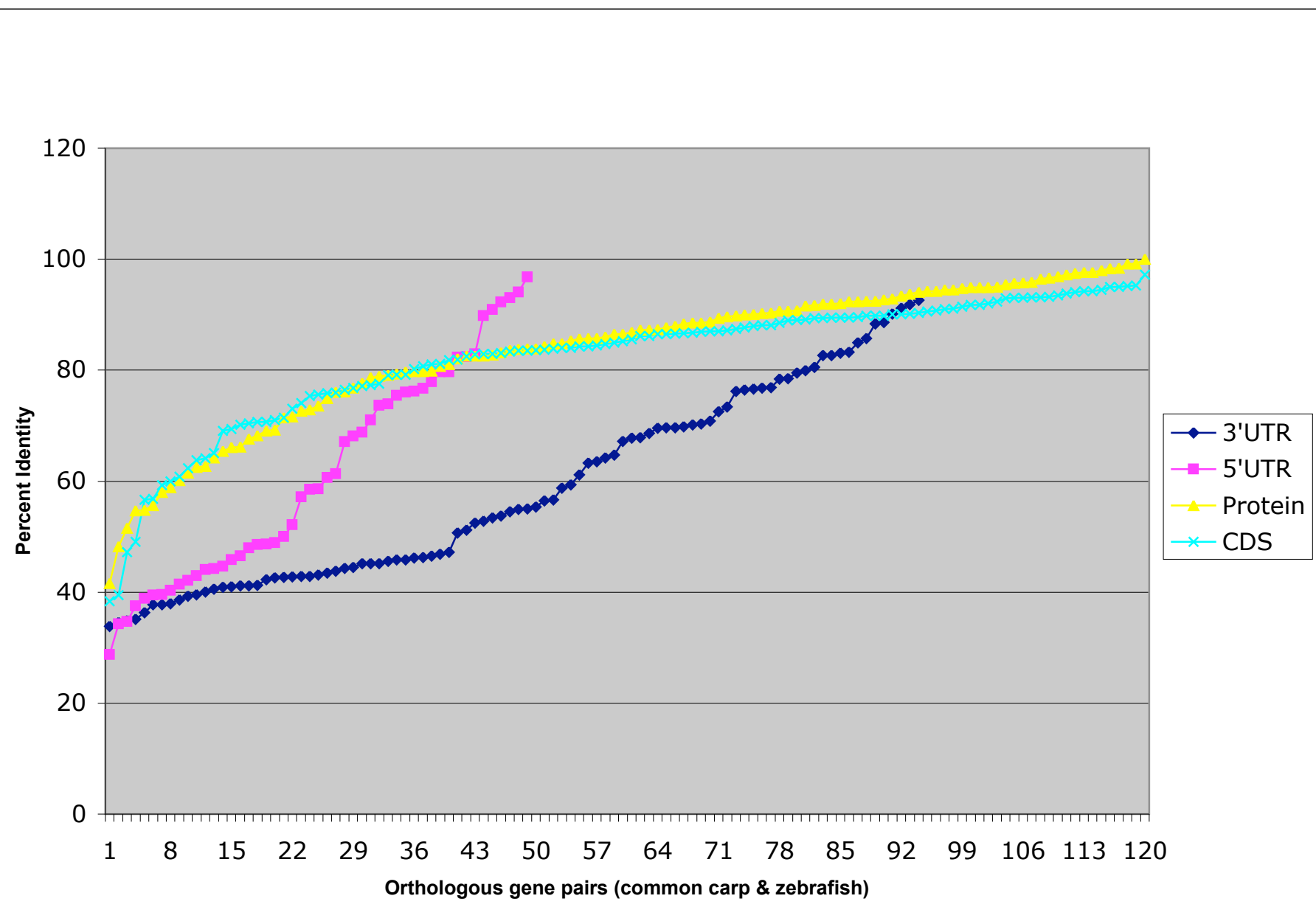

Supplement: Additional File 10 — Percent sequence identity between common carp and zebrafish orthologous proteins, CDS, 5' UTR and 3' UTR regions. [file 1471-2105-7-S5-S2-S10.pdf]
