# Supplementary material for: Comparative genomics in cyprinids: common carp ESTs help the annotation of the zebrafish genome
Source: BMC Bioinformatics. 2006 Dec 18;7(Suppl 5):S2. doi: 10.1186/1471-2105-7-S5-S2 (PMC1764476; doi:10.1186/1471-2105-7-S5-S2)
Supplement: Additional File 3 — Classification of 91 common carp ESTs that map to intergenic, intronic, ab initio predictions and non-zebrafish supported annotations. [file 1471-2105-7-S5-S2-S3.html]

## Table S3. List of mapped common carp transcripts overlapping intronic, intergenic and non-zebrafish ESTs/cDNA regions

|  |  |  |  |
| --- | --- | --- | --- |
| zebrafish  chromosome | chromosome position | zebrafish annotation | common carp clusterID  or GenBank ID |
| 2 | 36192769:36192991 | intergenic | DW722755 |
| 3 | 29265136:29265597 | intergenic | DW723876 |
| 20 | 30066449:30066578 | intergenic | DW724082 |
| 13 | 31275715:31276095 | intergenic | DW724248 |
| 22 | 33277352:33277792 | intergenic | DW721291 |
| 19 | 53710552:53710738 | intergenic | AU240324 |
| 23 | 55343449:55344008 | intergenic | DW723242 |
| 9 | 15621335:15621611 | intergenic | CA968995 |
| 19 | 31778487:31779030 | intergenic | DW721686 |
| 2 | 38731507:38731992 | intergenic | DW721395 |
| 7 | 25403156:25403676 | intergenic | CA969817 |
| 21 | 32618084:32623723 | intergenic | CA969518 |
| 1 | 37227841:37227994 | intergenic | CA967977 |
| 12 | 24542528:24542959 | Prediction | CA969665 |
| 10 | 21114165:21114473 | Prediction | CA968054 |
| 12 | 29941450:29941945 | Prediction | DW724156 |
| 9 | 17989309:17989842 | Prediction | CA965048 |
| 6 | 24239704:24239829 | Prediction | DW722097 |
| 7 | 55560525:55561302 | Prediction | CF661460 |
| 13 | 13669323:13669523 | Prediction | CA965770 |
| 16 | 29315215:29315857 | Prediction | 448 |
| 10 | 29354711:29355210 | Prediction | 34 |
| 23 | 42394671:42394824 | Prediction | CA969538 |
| 13 | 5197985:5214289 | Prediction | DW721345 |
| 16 | 35772064:35772586 | Prediction | CF662901 |
| 11 | 32902954:32903414 | Prediction | CA968791 |
| 12 | 28042268:28042592 | Prediction | CA964575 |
| 24 | 16631125:16631347 | Prediction | DW720892 |
| 18 | 13002698:13002860 | Prediction | DW722920 |
| 6 | 19417741:19418114 | Prediction | DW724184 |
| 11 | 32201832:32202183 | Prediction | CA969769 |
| 10 | 29336396:29337021 | Prediction | DW721433 |
| 6 | 1992175:1992418 | Prediction | DW724287 |
| 22 | 35266569:35266906 | Prediction | CA968932 |
| 10 | 6293156:6293304 | Prediction | DW722763 |
| 19 | 32344740:32345200 | zebrafish gene: intron | 1195 |
| 6 | 31010225:31010593 | zebrafish gene: intron | CF661933 |
| 6 | 13791526:13792001 | zebrafish gene: intron | CA969336 |
| 17 | 13420948:13421073 | zebrafish gene: intron | AU183368 |
| 24 | 7925119:7925523 | zebrafish gene: intron | CA969876 |
| 14 | 25694308:25695110 | zebrafish gene: intron | CF661031 |
| 15 | 31640728:31641094 | zebrafish gene: intron | CF661577 |
| 20 | 22840761:22841412 | zebrafish gene: intron | CA966816 |
| 22 | 22358872:22359160 | zebrafish gene: intron | DW719961 |
| 3 | 7550027:7550488 | zebrafish gene: intron | DW722928 |
| 2 | 39860289:39860895 | zebrafish gene: intron | DW722848 |
| 1 | 27503602:27506336 | zebrafish gene: intron | DW721285 |
| 18 | 13386453:13387080 | zebrafish gene: intron | CA969955 |
| 5 | 49943918:49944122 | zebrafish gene: intron | CA968239 |
| 10 | 11444113:11444354 | zebrafish gene: intron | DW721565 |
| 6 | 26339782:26342584 | zebrafish gene: intron | CF661379 |
| 23 | 45882331:45905810 | zebrafish gene: intron | CF662690 |
| 17 | 39292068:39292176 | zebrafish gene: intron | DW721679 |
| 7 | 34463759:34463866 | zebrafish gene: intron | CA970303 |
| 19 | 51447464:51447803 | zebrafish gene: intron | DW722551 |
| 13 | 10360841:10361419 | zebrafish gene: intron | DW720871 |
| 15 | 7327499:7327693 | zebrafish gene: intron | CA965670 |
| 8 | 36962988:36963064 | zebrafish gene: intron | CA969194 |
| 3 | 29602523:29603191 | non-zebrafish: intron | 409 |
| 14 | 64794372:64797309 | non-zebrafish: intron | 112 |
| 5 | 42105454:42106003 | non-zebrafish: intron | 788 |
| 12 | 22982576:22983128 | non-zebrafish: intron | CA967413 |
| 18 | 28048179:28048888 | non-zebrafish: intron | DW723349 |
| 24 | 6197416:6197891 | non-zebrafish: intron | 297 |
| 12 | 26466017:26466106 | non-zebrafish: intron | CA966466 |
| 13 | 5431809:5432219 | non-zebrafish: intron | DW724065 |
| 1 | 50713559:50713935 | non-zebrafish: intron | CA969200 |
| 12 | 18980749:18981292 | non-zebrafish: intron | CA969572 |
| 14 | 19057890:19058036 | non-zebrafish: intron | CF661917 |
| 1 | 11787546:11788033 | non-zebrafish: exon | 908 |
| 5 | 56497549:56498229 | non-zebrafish: exon | 1077 |
| 15 | 40385331:40385910 | non-zebrafish: exon | DW722641 |
| 21 | 29856966:29857823 | non-zebrafish: exon | CF660862 |
| 21 | 16142328:16147144 | non-zebrafish: exon | CA970399 |
| 19 | 26365278:26365433 | non-zebrafish: exon | CA964595 |
| 21 | 37053439:37056429 | non-zebrafish: exon | CF660617 |
| 17 | 25571503:25571599 | non-zebrafish: exon | DW719915 |
| 24 | 30295615:30309748 | non-zebrafish: exon | CF662750 |
| 2 | 7554455:7565675 | non-zebrafish: exon | CA964681 |
| 1 | 41248343:41248651 | non-zebrafish: exon | CA966405 |
| 20 | 12702354:12706940 | non-zebrafish: exon | CF662562 |
| 15 | 8213686:8217842 | non-zebrafish: exon | DW723943 |
| 16 | 51276175:51277771 | non-zebrafish: exon | CA969090 |
| 21 | 22827572:22828629 | non-zebrafish: exon | CA966451 |
| 22 | 35925053:35929545 | non-zebrafish: exon | CA966071 |
| 21 | 27009198:27021859 | non-zebrafish: exon | C88398 |
| 2 | 40395902:40406055 | non-zebrafish: exon | DW721296 |
| 16 | 20578438:20580986 | non-zebrafish: exon | CA964537 |
| 12 | 4483688:4489749 | non-zebrafish: exon | CA969772 |
| 22 | 28696378:28697081 | non-zebrafish: exon | CF661329 |
| 18 | 46228840:46239594 | non-zebrafish: exon | DW723921 |
