# Supplementary material for: Comparative genomics in cyprinids: common carp ESTs help the annotation of the zebrafish genome
Source: BMC Bioinformatics. 2006 Dec 18;7(Suppl 5):S2. doi: 10.1186/1471-2105-7-S5-S2 (PMC1764476; doi:10.1186/1471-2105-7-S5-S2)
Supplement: Additional File 4 — List of hyperlinks to potential common carp splice variants. Overlapping common carp and zebrafish transcripts are presented on a gbrowse viewer to highlight the missing exons in one of the two species. [file 1471-2105-7-S5-S2-S4.html]

## Table S4. List of potentially new splice variants in common carp ESTs

  


---

## "Missing" exons in common carp ESTs compared to zebrafish

chr1:17460744..17469414  
chr2:15421494..15427200  
chr4:22953511..22966506  
chr5:17969049..17973641  
chr5:67362723..67375378  
chr6:31160444..31168148  
chr7:28966037..28969886  
chr8:31443554..31463553  
chr11:26817445..26825919  
chr12:22512349..22552584  
chr12:32826400..32834566  
chr13:31728924..31733950  
chr15:42252795..42263537  
chr16:20284637..20293668  
chr16:21574848..21604737  
chr16:22957187..22965491  
chr16:27796577..27832834  
chr16:29578391..29590138  
chr19:59990651..59997741  
chr20:27874858..27891228  
chr21:16043398..16054443  
chr21:27008198..27022859  
chr22:25728116..25730329  
chr23:20328781..20331811  
chr23:51695991..51721876  
chr25:5007138..5008166

## "Extra" exons in common carp ESTs compared to zebrafish

chr1:42877555..42880444  
chr2:14526356..14537453  
chr4:21673927..21693926  
chr5:57175455..57186386  
chr6:19739096..19759095  
chr7:22039942..22049620  
chr7:54760020..54780019  
chr13:30829798..30837968  
chr13:32588773..32591819  
chr13:34487723..34491797  
chr13:39518533..39525475  
chr13:40545587..40556183  
chr16:42423685..42444119  
chr16:51276793..51283530  
chr17:30473851..30478669  
chr20:35531596..35541595
