# Supplementary material for: Comparative genomics in cyprinids: common carp ESTs help the annotation of the zebrafish genome
Source: BMC Bioinformatics. 2006 Dec 18;7(Suppl 5):S2. doi: 10.1186/1471-2105-7-S5-S2 (PMC1764476; doi:10.1186/1471-2105-7-S5-S2)
Supplement: Additional File 5 — List of 974 testis-only transcripts that do not overlap any of the zebrafish and fathead minnow ESTs. [file 1471-2105-7-S5-S2-S5.doc]

Table S5. List of 974 testis-only common carp transcripts that do not overlap any of the zebrafish and fathead minnow ESTs.

| **ClusterID** | GenBank IDs for common carp transcripts |
| --- | --- |
| ClusterID_1069 | DW719861,DW723222 |
| ClusterID_1201 | DW721040,DW721131,DW721275,DW721930 |
| ClusterID_1570 | DW723425,DW724149 |
| ClusterID_1335 | DW722316,DW722907 |
| ClusterID_1620 | DW724176,DW724323 |
| ClusterID_1525 | DW723856,DW723926 |
| ClusterID_1105 | DW719754,DW720117 |
| ClusterID_1612 | DW723576,DW724283 |
| ClusterID_1586 | DW724145,DW724195 |
| ClusterID_1348 | DW722546,DW722957 |
| ClusterID_1342 | DW720932,DW722925 |
| ClusterID_1017 | DW719706,DW719814 |
| ClusterID_1117 | DW720896,DW720903,DW720918 |
| ClusterID_1417 | DW720923,DW722005,DW722125,DW723380 |
| ClusterID_1070 | DW719694,DW719863 |
| ClusterID_1199 | DW721811,DW721855 |
| ClusterID_1613 | DW724270,DW724286 |
| ClusterID_1253 | DW721256,DW722313 |
| ClusterID_1077 | DW719704,DW719930 |
| ClusterID_1072 | DW719373,DW719470,DW719899 |
| ClusterID_1157 | DW720347,DW721413 |
| ClusterID_1183 | DW721049,DW721733 |
| ClusterID_1387 | DW722533,DW723211 |
| ClusterID_1177 | DW720881,DW721614 |
| ClusterID_1448 | DW722080,DW723564 |
| ClusterID_1041 | DW720327,DW721576 |
| ClusterID_1089 | DW719916,DW720045 |
| ClusterID_1633 | DW719599,DW722348,DW722508,DW722792,DW723174,DW723269, DW723714, DW723716, DW723992,DW724091,DW724205,DW724385 |
| ClusterID_1547 | DW720974,DW724049 |
| ClusterID_1282 | DW722349,DW722592 |
| ClusterID_1562 | DW723804,DW724097 |
| ClusterID_1372 | DW720511,DW723111 |
| ClusterID_1013 | DW719665,DW719673 |
| ClusterID_1165 | DW721468,DW721494 |
| ClusterID_1480 | DW719568,DW723722 |
| ClusterID_1141 | DW720655,DW721199 |
| ClusterID_1027 | DW719513,DW719827 |
| ClusterID_1539 | DW723975,DW723988 |
| ClusterID_1038 | DW720291,DW721625,DW723484 |
| ClusterID_1060 | DW720438,DW720540,DW720578,DW721610 |
| ClusterID_1125 | DW720898,DW721010 |
| ClusterID_1305 | DW721924,DW722739 |
| singleton | DW722774 |
| singleton | DW722785 |
| singleton | DW722789 |
| singleton | DW722791 |
| singleton | DW722808 |
| singleton | DW722816 |
| singleton | DW722818 |
| singleton | DW722826 |
| singleton | DW722832 |
| singleton | DW722848 |
| singleton | DW722851 |
| singleton | DW722857 |
| singleton | DW722859 |
| singleton | DW722871 |
| singleton | DW722881 |
| singleton | DW722891 |
| singleton | DW722922 |
| singleton | DW722940 |
| singleton | DW722981 |
| singleton | DW722987 |
| singleton | DW722999 |
| singleton | DW723019 |
| singleton | DW723029 |
| singleton | DW723030 |
| singleton | DW723053 |
| singleton | DW723055 |
| singleton | DW723066 |
| singleton | DW723067 |
| singleton | DW723074 |
| singleton | DW723080 |
| singleton | DW723081 |
| singleton | DW723084 |
| singleton | DW723088 |
| singleton | DW723112 |
| singleton | DW723121 |
| singleton | DW723130 |
| singleton | DW723148 |
| singleton | DW723158 |
| singleton | DW723165 |
| singleton | DW723173 |
| singleton | DW723176 |
| singleton | DW723182 |
| singleton | DW723186 |
| singleton | DW723213 |
| singleton | DW723228 |
| singleton | DW723230 |
| singleton | DW723239 |
| singleton | DW723244 |
| singleton | DW723275 |
| singleton | DW723282 |
| singleton | DW723296 |
| singleton | DW723302 |
| singleton | DW723309 |
| singleton | DW723310 |
| singleton | DW723365 |
| singleton | DW723435 |
| singleton | DW723442 |
| singleton | DW723447 |
| singleton | DW723452 |
| singleton | DW723463 |
| singleton | DW723479 |
| singleton | DW723482 |
| singleton | DW723495 |
| singleton | DW723501 |
| singleton | DW723521 |
| singleton | DW723524 |
| singleton | DW723532 |
| singleton | DW723534 |
| singleton | DW723536 |
| singleton | DW723581 |
| singleton | DW723585 |
| singleton | DW723592 |
| singleton | DW723605 |
| singleton | DW723614 |
| singleton | DW723615 |
| singleton | DW723625 |
| singleton | DW723639 |
| singleton | DW723662 |
| singleton | DW723672 |
| singleton | DW723700 |
| singleton | DW723709 |
| singleton | DW723711 |
| singleton | DW723713 |
| singleton | DW723720 |
| singleton | DW723731 |
| singleton | DW723753 |
| singleton | DW723759 |
| singleton | DW723760 |
| singleton | DW723761 |
| singleton | DW723769 |
| singleton | DW723770 |
| singleton | DW723773 |
| singleton | DW723797 |
| singleton | DW723801 |
| singleton | DW723805 |
| singleton | DW723813 |
| singleton | DW723826 |
| singleton | DW723832 |
| singleton | DW723835 |
| singleton | DW723836 |
| singleton | DW723841 |
| singleton | DW723849 |
| singleton | DW723855 |
| singleton | DW723859 |
| singleton | DW723860 |
| singleton | DW723870 |
| singleton | DW723872 |
| singleton | DW723876 |
| singleton | DW723881 |
| singleton | DW723882 |
| singleton | DW723891 |
| singleton | DW723894 |
| singleton | DW723924 |
| singleton | DW723963 |
| singleton | DW723976 |
| singleton | DW723987 |
| singleton | DW723989 |
| singleton | DW723990 |
| singleton | DW723998 |
| singleton | DW724003 |
| singleton | DW724012 |
| singleton | DW724022 |
| singleton | DW724031 |
| singleton | DW724038 |
| singleton | DW724051 |
| singleton | DW724058 |
| singleton | DW724067 |
| singleton | DW724068 |
| singleton | DW724077 |
| singleton | DW724085 |
| singleton | DW724086 |
| singleton | DW724107 |
| singleton | DW724116 |
| singleton | DW724120 |
| singleton | DW724129 |
| singleton | DW724140 |
| singleton | DW724156 |
| singleton | DW724183 |
| singleton | DW724184 |
| singleton | DW724193 |
| singleton | DW724194 |
| singleton | DW724203 |
| singleton | DW724234 |
| singleton | DW724244 |
| singleton | DW724248 |
| singleton | DW724263 |
| singleton | DW724268 |
| singleton | DW724273 |
| singleton | DW724289 |
| singleton | DW724290 |
| singleton | DW724295 |
| singleton | DW724296 |
| singleton | DW724306 |
| singleton | DW724338 |
| singleton | DW724345 |
| singleton | DW724349 |
| singleton | DW724353 |
| singleton | DW724355 |
| singleton | DW724375 |
| singleton | DW724384 |
| singleton | DW724390 |
| singleton | DW724398 |
| singleton | DW724399 |
| singleton | DW724407 |
| singleton | DW724411 |
| singleton | DW724414 |
| singleton | DW724422 |
| singleton | DW719357 |
| singleton | DW719394 |
| singleton | DW719407 |
| singleton | DW719415 |
| singleton | DW719426 |
| singleton | DW719448 |
| singleton | DW719492 |
| singleton | DW719502 |
| singleton | DW719510 |
| singleton | DW719519 |
| singleton | DW719520 |
| singleton | DW719538 |
| singleton | DW719593 |
| singleton | DW719608 |
| singleton | DW719624 |
| singleton | DW719656 |
| singleton | DW719658 |
| singleton | DW719661 |
| singleton | DW719664 |
| singleton | DW719677 |
| singleton | DW719691 |
| singleton | DW719701 |
| singleton | DW719719 |
| singleton | DW719722 |
| singleton | DW719743 |
| singleton | DW719747 |
| singleton | DW719750 |
| singleton | DW719757 |
| singleton | DW719765 |
| singleton | DW719774 |
| singleton | DW719775 |
| singleton | DW719778 |
| singleton | DW719787 |
| singleton | DW719797 |
| singleton | DW719799 |
| singleton | DW719820 |
| singleton | DW719831 |
| singleton | DW720222 |
| singleton | DW720393 |
| singleton | DW720524 |
| singleton | DW719852 |
| singleton | DW719854 |
| singleton | DW719856 |
| singleton | DW719857 |
| singleton | DW719870 |
| singleton | DW719871 |
| singleton | DW719873 |
| singleton | DW719877 |
| singleton | DW719883 |
| singleton | DW719896 |
| singleton | DW719906 |
| singleton | DW719908 |
| singleton | DW719918 |
| singleton | DW719926 |
| singleton | DW719928 |
| singleton | DW719929 |
| singleton | DW719934 |
| singleton | DW719938 |
| singleton | DW719941 |
| singleton | DW719949 |
| singleton | DW719951 |
| singleton | DW719956 |
| singleton | DW719961 |
| singleton | DW719962 |
| singleton | DW719964 |
| singleton | DW719965 |
| singleton | DW719969 |
| singleton | DW719971 |
| singleton | DW719975 |
| singleton | DW719990 |
| singleton | DW719991 |
| singleton | DW719997 |
| singleton | DW720000 |
| singleton | DW720006 |
| singleton | DW720007 |
| singleton | DW720010 |
| singleton | DW720021 |
| singleton | DW720038 |
| singleton | DW720044 |
| singleton | DW720075 |
| singleton | DW720078 |
| singleton | DW720092 |
| singleton | DW720618 |
| singleton | DW720627 |
| singleton | DW720640 |
| singleton | DW720672 |
| singleton | DW720676 |
| singleton | DW720694 |
| singleton | DW720701 |
| singleton | DW720704 |
| singleton | DW720709 |
| singleton | DW720720 |
| singleton | DW720734 |
| singleton | DW720735 |
| singleton | DW720751 |
| singleton | DW720838 |
| singleton | DW720871 |
| singleton | DW720892 |
| singleton | DW720897 |
| singleton | DW720960 |
| singleton | DW720962 |
| singleton | DW720980 |
| singleton | DW720984 |
| singleton | DW720991 |
| singleton | DW721001 |
| singleton | DW721004 |
| singleton | DW721019 |
| singleton | DW721026 |
| singleton | DW721039 |
| singleton | DW721050 |
| singleton | DW721066 |
| singleton | DW721067 |
| singleton | DW721100 |
| singleton | DW721102 |
| singleton | DW721114 |
| singleton | DW721118 |
| singleton | DW721129 |
| singleton | DW721132 |
| singleton | DW721138 |
| singleton | DW721160 |
| singleton | DW721168 |
| singleton | DW721189 |
| singleton | DW721216 |
| singleton | DW721219 |
| singleton | DW721225 |
| singleton | DW721231 |
| singleton | DW721234 |
| singleton | DW721248 |
| singleton | DW721249 |
| singleton | DW721254 |
| singleton | DW721281 |
| singleton | DW721366 |
| singleton | DW721388 |
| singleton | DW721393 |
| singleton | DW721395 |
| singleton | DW721407 |
| singleton | DW721447 |
| singleton | DW721452 |
| singleton | DW721466 |
| singleton | DW721470 |
| singleton | DW721480 |
| singleton | DW721484 |
| singleton | DW721504 |
| singleton | DW721508 |
| singleton | DW721510 |
| singleton | DW721520 |
| singleton | DW721527 |
| singleton | DW721529 |
| singleton | DW721559 |
| singleton | DW721562 |
| singleton | DW721565 |
| singleton | DW721571 |
| singleton | DW721586 |
| singleton | DW721588 |
| singleton | DW721628 |
| singleton | DW721633 |
| singleton | DW721641 |
| singleton | DW721651 |
| singleton | DW721679 |
| singleton | DW721682 |
| singleton | DW721686 |
| singleton | DW721735 |
| singleton | DW721764 |
| singleton | DW721786 |
| singleton | DW721798 |
| singleton | DW721806 |
| singleton | DW721836 |
| singleton | DW721839 |
| singleton | DW721856 |
| singleton | DW721866 |
| singleton | DW721878 |
| singleton | DW721881 |
| singleton | DW721883 |
| singleton | DW721885 |
| singleton | DW721893 |
| singleton | DW721911 |
| singleton | DW721922 |
| singleton | DW721931 |
| singleton | DW721934 |
| singleton | DW721956 |
| singleton | DW721972 |
| singleton | DW721974 |
| singleton | DW721996 |
| singleton | DW722007 |
| singleton | DW722023 |
| singleton | DW722032 |
| singleton | DW722033 |
| singleton | DW722094 |
| singleton | DW722096 |
| singleton | DW722097 |
| singleton | DW722103 |
| singleton | DW722105 |
| singleton | DW722110 |
| singleton | DW722121 |
| singleton | DW722135 |
| singleton | DW722136 |
| singleton | DW722138 |
| singleton | DW722139 |
| singleton | DW722147 |
| singleton | DW722149 |
| singleton | DW722173 |
| singleton | DW722200 |
| singleton | DW722202 |
| singleton | DW722205 |
| singleton | DW722207 |
| singleton | DW722224 |
| singleton | DW722225 |
| singleton | DW722231 |
| singleton | DW722244 |
| singleton | DW722245 |
| singleton | DW722252 |
| singleton | DW722261 |
| singleton | DW722282 |
| singleton | DW722300 |
| singleton | DW722309 |
| singleton | DW722324 |
| singleton | DW722326 |
| singleton | DW722340 |
| singleton | DW722346 |
| singleton | DW722350 |
| singleton | DW722363 |
| singleton | DW722384 |
| singleton | DW722394 |
| singleton | DW722404 |
| singleton | DW722410 |
| singleton | DW722417 |
| singleton | DW722421 |
| singleton | DW722433 |
| singleton | DW722441 |
| singleton | DW722444 |
| singleton | DW722453 |
| singleton | DW722457 |
| singleton | DW722466 |
| singleton | DW722484 |
| singleton | DW722496 |
| singleton | DW722525 |
| singleton | DW722535 |
| singleton | DW722549 |
| singleton | DW722550 |
| singleton | DW722551 |
| singleton | DW722563 |
| singleton | DW722567 |
| singleton | DW722573 |
| singleton | DW722613 |
| singleton | DW722619 |
| singleton | DW722631 |
| singleton | DW722647 |
| singleton | DW722653 |
| singleton | DW722671 |
| singleton | DW722674 |
| singleton | DW722689 |
| singleton | DW722690 |
| singleton | DW722697 |
| singleton | DW722724 |
| singleton | DW722741 |
| singleton | DW722745 |
| singleton | DW722755 |
| singleton | DW722758 |
| singleton | DW722759 |
| singleton | DW722767 |
| singleton | DW722819 |
| singleton | DW722889 |
| singleton | DW722919 |
| singleton | DW722938 |
| singleton | DW722960 |
| singleton | DW723011 |
| singleton | DW723036 |
| singleton | DW723060 |
| singleton | DW723070 |
| singleton | DW723089 |
| singleton | DW723114 |
| singleton | DW723127 |
| singleton | DW723138 |
| singleton | DW723218 |
| singleton | DW723289 |
| singleton | DW723298 |
| singleton | DW723303 |
| singleton | DW723368 |
| singleton | DW723396 |
| singleton | DW723455 |
| singleton | DW723502 |
| singleton | DW723589 |
| singleton | DW723633 |
| singleton | DW723663 |
| singleton | DW723698 |
| singleton | DW723734 |
| singleton | DW723775 |
| singleton | DW723778 |
| singleton | DW723784 |
| singleton | DW723787 |
| singleton | DW723837 |
| singleton | DW723839 |
| singleton | DW723885 |
| singleton | DW723901 |
| singleton | DW723908 |
| singleton | DW723909 |
| singleton | DW723919 |
| singleton | DW723945 |
| singleton | DW723968 |
| singleton | DW724002 |
| singleton | DW724011 |
| singleton | DW724027 |
| singleton | DW724047 |
| singleton | DW724056 |
| singleton | DW724061 |
| singleton | DW724108 |
| singleton | DW724138 |
| singleton | DW724154 |
| singleton | DW724170 |
| singleton | DW724179 |
| singleton | DW724185 |
| singleton | DW724206 |
| singleton | DW724237 |
| singleton | DW724259 |
| singleton | DW724262 |
| singleton | DW724317 |
| singleton | DW724361 |
| singleton | DW724380 |
| singleton | DW724388 |
| singleton | DW724391 |
| singleton | DW719354 |
| singleton | DW719364 |
| singleton | DW719380 |
| singleton | DW719395 |
| singleton | DW719397 |
| singleton | DW719420 |
| singleton | DW719432 |
| singleton | DW719473 |
| singleton | DW719556 |
| singleton | DW719596 |
| singleton | DW719597 |
| singleton | DW719626 |
| singleton | DW719635 |
| singleton | DW719702 |
| singleton | DW719720 |
| singleton | DW719810 |
| singleton | DW720240 |
| singleton | DW720437 |
| singleton | DW719865 |
| singleton | DW719888 |
| singleton | DW719892 |
| singleton | DW719909 |
| singleton | DW719913 |
| singleton | DW719948 |
| singleton | DW719974 |
| singleton | DW720061 |
| singleton | DW720073 |
| singleton | DW720639 |
| singleton | DW720662 |
| singleton | DW720680 |
| singleton | DW720685 |
| singleton | DW720697 |
| singleton | DW720807 |
| singleton | DW720824 |
| singleton | DW720826 |
| singleton | DW720858 |
| singleton | DW720929 |
| singleton | DW720938 |
| singleton | DW720955 |
| singleton | DW720963 |
| singleton | DW720989 |
| singleton | DW721084 |
| singleton | DW721086 |
| singleton | DW721094 |
| singleton | DW721115 |
| singleton | DW721137 |
| singleton | DW721178 |
| singleton | DW721211 |
| singleton | DW721423 |
| singleton | DW721424 |
| singleton | DW721433 |
| singleton | DW721454 |
| singleton | DW721463 |
| singleton | DW721499 |
| singleton | DW721502 |
| singleton | DW721506 |
| singleton | DW721567 |
| singleton | DW721653 |
| singleton | DW721670 |
| singleton | DW721705 |
| singleton | DW721748 |
| singleton | DW721749 |
| singleton | DW721753 |
| singleton | DW721758 |
| singleton | DW721784 |
| singleton | DW721790 |
| singleton | DW721837 |
| singleton | DW721874 |
| singleton | DW721882 |
| singleton | DW721908 |
| singleton | DW721981 |
| singleton | DW722076 |
| singleton | DW722093 |
| singleton | DW722101 |
| singleton | DW722118 |
| singleton | DW722124 |
| singleton | DW722134 |
| singleton | DW722148 |
| singleton | DW722165 |
| singleton | DW722194 |
| singleton | DW722212 |
| singleton | DW722219 |
| singleton | DW722221 |
| singleton | DW722278 |
| singleton | DW722297 |
| singleton | DW722330 |
| singleton | DW722347 |
| singleton | DW722375 |
| singleton | DW722414 |
| singleton | DW722480 |
| singleton | DW722481 |
| singleton | DW722505 |
| singleton | DW722536 |
| singleton | DW722548 |
| singleton | DW722574 |
| singleton | DW722605 |
| singleton | DW722629 |
| singleton | DW722641 |
| singleton | DW722648 |
| singleton | DW722660 |
| singleton | DW722665 |
| singleton | DW722700 |
| singleton | DW722706 |
| singleton | DW722709 |
| singleton | DW722713 |
| singleton | DW722736 |
| singleton | DW719368 |
| singleton | DW719404 |
| singleton | DW719428 |
| singleton | DW719438 |
| singleton | DW719486 |
| singleton | DW719497 |
| singleton | DW719506 |
| singleton | DW719527 |
| singleton | DW719594 |
| singleton | DW719613 |
| singleton | DW719616 |
| singleton | DW719642 |
| singleton | DW719712 |
| singleton | DW719721 |
| singleton | DW719733 |
| singleton | DW720175 |
| singleton | DW720287 |
| singleton | DW720363 |
| singleton | DW720538 |
| singleton | DW720546 |
| singleton | DW719996 |
| singleton | DW720009 |
| singleton | DW720034 |
| singleton | DW720042 |
| singleton | DW720069 |
| singleton | DW720074 |
| singleton | DW720714 |
| singleton | DW720811 |
| singleton | DW720817 |
| singleton | DW720818 |
| singleton | DW720827 |
| singleton | DW720861 |
| singleton | DW720926 |
| singleton | DW720944 |
| singleton | DW720949 |
| singleton | DW720971 |
| singleton | DW720994 |
| singleton | DW721011 |
| singleton | DW721020 |
| singleton | DW721021 |
| singleton | DW721027 |
| singleton | DW721075 |
| singleton | DW721088 |
| singleton | DW721119 |
| singleton | DW721120 |
| singleton | DW721159 |
| singleton | DW721188 |
| singleton | DW721269 |
| singleton | DW721536 |
| singleton | DW721557 |
| singleton | DW721630 |
| singleton | DW721654 |
| singleton | DW721656 |
| singleton | DW721662 |
| singleton | DW721694 |
| singleton | DW721760 |
| singleton | DW721791 |
| singleton | DW721832 |
| singleton | DW721864 |
| singleton | DW721905 |
| singleton | DW721907 |
| singleton | DW721917 |
| singleton | DW721943 |
| singleton | DW721968 |
| singleton | DW722031 |
| singleton | DW722036 |
| singleton | DW722041 |
| singleton | DW722203 |
| singleton | DW722333 |
| singleton | DW722337 |
| singleton | DW722387 |
| singleton | DW722419 |
| singleton | DW722506 |
| singleton | DW722538 |
| singleton | DW722571 |
| singleton | DW722612 |
| singleton | DW722698 |
| singleton | DW722734 |
| singleton | DW722766 |
| singleton | DW722793 |
| singleton | DW722831 |
| singleton | DW722852 |
| singleton | DW722860 |
| singleton | DW722923 |
| singleton | DW722935 |
| singleton | DW722990 |
| singleton | DW723046 |
| singleton | DW723069 |
| singleton | DW723169 |
| singleton | DW723172 |
| singleton | DW723181 |
| singleton | DW723207 |
| singleton | DW723237 |
| singleton | DW723251 |
| singleton | DW723262 |
| singleton | DW723285 |
| singleton | DW723323 |
| singleton | DW723374 |
| singleton | DW723475 |
| singleton | DW723485 |
| singleton | DW723514 |
| singleton | DW723535 |
| singleton | DW723571 |
| singleton | DW723622 |
| singleton | DW723691 |
| singleton | DW723696 |
| singleton | DW723754 |
| singleton | DW723796 |
| singleton | DW723800 |
| singleton | DW723808 |
| singleton | DW723991 |
| singleton | DW723999 |
| singleton | DW724041 |
| singleton | DW724053 |
| singleton | DW724132 |
| singleton | DW724218 |
| singleton | DW724220 |
| singleton | DW724236 |
| singleton | DW724271 |
| singleton | DW724278 |
| singleton | DW724279 |
| singleton | DW724332 |
| singleton | DW719352 |
| singleton | DW719370 |
| singleton | DW719401 |
| singleton | DW719409 |
| singleton | DW719437 |
| singleton | DW719471 |
| singleton | DW719481 |
| singleton | DW719509 |
| singleton | DW719511 |
| singleton | DW719516 |
| singleton | DW719526 |
| singleton | DW719539 |
| singleton | DW719541 |
| singleton | DW719550 |
| singleton | DW719551 |
| singleton | DW719552 |
| singleton | DW719553 |
| singleton | DW719583 |
| singleton | DW719600 |
| singleton | DW719612 |
| singleton | DW719639 |
| singleton | DW719647 |
| singleton | DW719657 |
| singleton | DW719685 |
| singleton | DW719688 |
| singleton | DW719692 |
| singleton | DW719713 |
| singleton | DW719723 |
| singleton | DW719728 |
| singleton | DW719756 |
| singleton | DW719759 |
| singleton | DW719766 |
| singleton | DW719789 |
| singleton | DW719843 |
| singleton | DW719846 |
| singleton | DW719848 |
| singleton | DW720171 |
| singleton | DW720179 |
| singleton | DW720227 |
| singleton | DW720379 |
| singleton | DW720394 |
| singleton | DW720441 |
| singleton | DW719860 |
| singleton | DW719878 |
| singleton | DW719957 |
| singleton | DW719958 |
| singleton | DW719963 |
| singleton | DW719981 |
| singleton | DW719985 |
| singleton | DW719986 |
| singleton | DW719989 |
| singleton | DW720053 |
| singleton | DW720062 |
| singleton | DW720076 |
| singleton | DW720079 |
| singleton | DW720088 |
| singleton | DW720623 |
| singleton | DW720637 |
| singleton | DW720659 |
| singleton | DW720101 |
| singleton | DW720103 |
| singleton | DW720819 |
| singleton | DW720830 |
| singleton | DW720840 |
| singleton | DW720859 |
| singleton | DW720879 |
| singleton | DW720882 |
| singleton | DW720884 |
| singleton | DW720920 |
| singleton | DW721003 |
| singleton | DW721009 |
| singleton | DW721014 |
| singleton | DW721023 |
| singleton | DW721037 |
| singleton | DW721046 |
| singleton | DW721078 |
| singleton | DW721122 |
| singleton | DW721127 |
| singleton | DW721156 |
| singleton | DW721222 |
| singleton | DW721237 |
| singleton | DW721243 |
| singleton | DW721253 |
| singleton | DW721284 |
| singleton | DW721354 |
| singleton | DW721382 |
| singleton | DW721396 |
| singleton | DW721472 |
| singleton | DW721505 |
| singleton | DW721519 |
| singleton | DW721523 |
| singleton | DW721541 |
| singleton | DW721611 |
| singleton | DW721615 |
| singleton | DW721624 |
| singleton | DW721634 |
| singleton | DW721667 |
| singleton | DW721673 |
| singleton | DW721676 |
| singleton | DW721704 |
| singleton | DW721724 |
| singleton | DW721731 |
| singleton | DW721737 |
| singleton | DW721739 |
| singleton | DW721773 |
| singleton | DW721777 |
| singleton | DW721782 |
| singleton | DW721797 |
| singleton | DW721813 |
| singleton | DW721846 |
| singleton | DW721859 |
| singleton | DW721861 |
| singleton | DW721876 |
| singleton | DW721879 |
| singleton | DW721886 |
| singleton | DW721891 |
| singleton | DW721895 |
| singleton | DW721898 |
| singleton | DW721901 |
| singleton | DW721904 |
| singleton | DW721941 |
| singleton | DW721945 |
| singleton | DW722027 |
| singleton | DW722035 |
| singleton | DW722045 |
| singleton | DW722057 |
| singleton | DW722060 |
| singleton | DW722068 |
| singleton | DW722082 |
| singleton | DW722137 |
| singleton | DW722184 |
| singleton | DW722195 |
| singleton | DW722204 |
| singleton | DW722226 |
| singleton | DW722254 |
| singleton | DW722292 |
| singleton | DW722354 |
| singleton | DW722382 |
| singleton | DW722436 |
| singleton | DW722463 |
| singleton | DW722476 |
| singleton | DW722482 |
| singleton | DW722489 |
| singleton | DW722491 |
| singleton | DW722495 |
| singleton | DW722497 |
| singleton | DW722502 |
| singleton | DW722524 |
| singleton | DW722545 |
| singleton | DW722558 |
| singleton | DW722568 |
| singleton | DW722623 |
| singleton | DW722640 |
| singleton | DW722643 |
| singleton | DW722644 |
| singleton | DW722676 |
| singleton | DW722691 |
| singleton | DW722704 |
| singleton | DW722744 |
| singleton | DW722843 |
| singleton | DW722850 |
| singleton | DW722882 |
| singleton | DW722885 |
| singleton | DW722921 |
| singleton | DW722998 |
| singleton | DW723024 |
| singleton | DW723065 |
| singleton | DW723107 |
| singleton | DW723135 |
| singleton | DW723180 |
| singleton | DW723245 |
| singleton | DW723273 |
| singleton | DW723276 |
| singleton | DW723308 |
| singleton | DW723339 |
| singleton | DW723366 |
| singleton | DW723437 |
| singleton | DW723476 |
| singleton | DW723489 |
| singleton | DW723490 |
| singleton | DW723497 |
| singleton | DW723512 |
| singleton | DW723554 |
| singleton | DW723556 |
| singleton | DW723563 |
| singleton | DW723573 |
| singleton | DW723578 |
| singleton | DW723648 |
| singleton | DW723649 |
| singleton | DW723755 |
| singleton | DW723756 |
| singleton | DW723782 |
| singleton | DW723799 |
| singleton | DW723831 |
| singleton | DW723878 |
| singleton | DW723895 |
| singleton | DW723937 |
| singleton | DW723940 |
| singleton | DW723952 |
| singleton | DW723969 |
| singleton | DW723980 |
| singleton | DW723982 |
| singleton | DW724033 |
| singleton | DW724042 |
| singleton | DW724071 |
| singleton | DW724109 |
| singleton | DW724114 |
| singleton | DW724204 |
| singleton | DW724313 |
| singleton | DW724316 |
| singleton | DW724356 |
| singleton | DW724358 |
| singleton | DW724363 |
| singleton | DW724368 |
| singleton | DW724374 |
| singleton | DW724377 |
| singleton | DW724383 |
