# Supplementary material for: Comparative genomics in cyprinids: common carp ESTs help the annotation of the zebrafish genome
Source: BMC Bioinformatics. 2006 Dec 18;7(Suppl 5):S2. doi: 10.1186/1471-2105-7-S5-S2 (PMC1764476; doi:10.1186/1471-2105-7-S5-S2)
Supplement: Additional File 6 — List of 474 testis-derived clusters that show sequence identity to 474 zebrafish and 75 Fathead minnow UniGene clusters. Testis-expression information was added to the adult-stage zebrafish expression data. [file 1471-2105-7-S5-S2-S6.doc]

Table S6. List of 474 testis-derived common carp clusters that show sequence identity to 474 zebrafish and 75 fathead minnow UniGene clusters. (Information on testis-expression was added to the adult stage zebrafish expression data.)

| ClusterID | Zebrafish  UniGene-ID | Fathead Minnow  UniGene-ID | GenBank IDs for common carp transcripts |
| --- | --- | --- | --- |
| 1169 | Dr.31066 | Ppr.1845 | CF662458 CF662404 CF662283 CA964458 CA970341 CA970339 DW719485 DW719840 DW721577 DW722462 DW723929 |
| 1049 | Dr.29141 | Ppr.7341 | DW720255 DW720442 DW720454 DW720482 DW720556 DW720621 DW720702 |
| 1588 | Dr.10451 | no match | AB063389 DW724210 |
| 1410 | Dr.27037 | no match | CF662178 CF661632 DW723342 |
| 1481 | Dr.8283 | no match | CA969805 DW723725 |
| 1434 | Dr.6963 | no match | CA966703 CF661035 CF663040 CA966781 CA966586 CA965253 CA968238 CA967557 AU081423 C88396 DW722685 DW723477 |
| 1050 | Dr.29754 | no match | CF663069 CF662470 CA967658 DW720449 |
| 1488 | Dr.38310 | no match | CF662803 CA965774 CA969759 DW719732 DW723766 |
| 1619 | Dr.1430 | no match | CA966667 DW719570 DW721265 DW722694 DW724310 |
| 1218 | Dr.19646 | no match | CA967135 DW720234 DW720805 DW721106 DW721374 DW721547 DW721787 DW721973 DW722086 DW722095 DW722305 DW722334 DW722358 DW722438 DW722905 DW723389 DW723448 DW723569 DW723570 DW723708 DW723892 |
| 1193 | Dr.16675 | no match | CA964245 DW721814 |
| 1404 | Dr.24671 | no match | CF662138 CF661869 CF661739 CA964794 CA964770 CO729437 AU052041 C88382 DW720869 DW721596 DW721695 DW722306 DW722393 DW723317 |
| 1031 | Dr.43161 | no match | CF662101 CF662003 CF661942 CF661901 CF661706 CF661506 CF662570 CF662559 CA966018 CA965255 CA965254 CA970248 CA967332 CO729413 CA968685 AU312531 AU312521 AU312482 AU301700 AU301592 AU301561 AU301197 AU240366 AU183469 AU183355 AF528161 D88120 D88119 D88118 D88117 D88116 D88115 BetaGlobin DW720124 DW720125 DW720127 DW720137 DW720138 DW720164 DW720180 DW720191 DW720192 DW720195 DW720205 DW720233 DW720242 DW720244 DW720248 DW720253 DW720256 DW720258 DW720262 DW720266 DW720277 DW720285 DW720292 DW720298 DW720301 DW720309 DW720317 DW720322 DW720333 DW720337 DW720343 DW720359 DW720362 DW720368 DW720380 DW720395 DW720408 DW720418 DW720426 DW720430 DW720432 DW720447 DW720452 DW720461 DW720465 DW720466 DW720473 DW720481 DW720493 DW720501 DW720510 DW720527 DW720562 DW720574 DW720581 DW720611 DW720614 DW720616 DW719855 DW720629 DW720646 DW720648 DW720658 DW720664 DW720674 DW720689 DW720717 DW720722 DW720739 DW720741 DW720742 DW720744 DW720746 DW720747 DW720760 DW720766 DW720767 DW720772 DW720774 DW720787 DW720821 DW720921 DW720970 DW721098 DW721511 DW721665 DW721697 DW721744 DW721845 DW721890 DW721894 DW722167 DW722289 DW722336 DW722357 DW722620 DW722625 DW722837 DW722875 DW722966 DW723272 DW723478 DW724217 DW724419 |
| 1020 | Dr.15561 | Ppr.18231 | DW719408 DW719738 |
| 1543 | Dr.23662 | no match | CA966723 CA964610 CA968958 DW724025 |
| 1224 | Dr.17325 | no match | CA970196 DW722133 |
| 1273 | Dr.664 | no match | CF662092 CF662040 CA964816 CA970384 AU301640 AU301030 DW719880 DW720889 DW722277 DW722490 |
| 1490 | Dr.1329 | no match | CF661855 CA964769 CA968765 AU301080 AU301067 DW719484 DW722018 DW723506 DW723550 DW723780 |
| 1625 | Dr.941 | no match | CA966754 CA967399 DW724337 |
| 1364 | Dr.26820 | no match | CA965512 DW721268 DW722942 DW723085 |
| 1312 | Dr.16048 | no match | CF662485 DW722784 |
| 1408 | Dr.28298 | no match | CF661485 CF662574 DW723334 |
| 1250 | Dr.33885 | Ppr.12187 | CF662205 CF662196 CF661988 CA964813 CA964126 CA970223 DW721398 DW721507 DW722284 DW722456 DW723763 |
| 1146 | Dr.33647 | no match | CA967737 DW721255 |
| 994 | Dr.29090 | no match | CA966256 DW719544 |
| 1257 | Dr.28873 | no match | DW722228 DW722368 |
| 1363 | Dr.3900 | no match | DW723027 DW723071 |
| 1370 | Dr.3211 | no match | CA968428 DW721648 DW723105 |
| 1531 | Dr.29782 | Ppr.18260 | CA968807 CA968392 CA967609 DW723948 |
| 584 | Dr.30628 | no match | CF661703 CF661072 CF660589 CF660573 CF660473 CF663092 CF663091 CF660456 CF660387 CF662837 CF662448 CF662421 CA966674 CA966640 CA966363 CA966296 CA965526 CA965437 CA965431 CA965137 CA965126 CA965088 CA964559 CA964546 CA964463 CA964412 CA964321 CA964302 CA964014 CA964005 CA970366 CA970352 CA970333 CA970313 CA970297 CA970296 CA970069 CA969568 CA969169 CA968515 CA968405 CA968404 CA968386 CA968377 CA968355 CA968352 CA968344 CA968296 CA968222 CA968177 CA968162 CA968160 CA968145 CA968140 CA967972 CA967963 CA967962 CA967866 CA967851 CA967821 CA967820 CA967589 CA967559 CA967535 AJ870982 DW723781 DW724412 |
| 988 | Dr.14434 | no match | DW719504 DW720014 |
| 1263 | Dr.7352 | no match | CA967267 DW722398 |
| 1630 | Dr.2446 | no match | CA965221 DW724366 |
| 1597 | Dr.7102 | no match | CF662383 CF662365 CA965263 CA967860 DW722748 DW723118 DW723692 DW724110 DW724232 |
| 1454 | Dr.1506 | no match | DW722936 DW723591 |
| 1553 | Dr.27409 | no match | DW720880 DW721481 DW721542 DW721600 DW723079 DW723574 DW723710 DW724069 |
| 1523 | Dr.5605 | Ppr.8903 | CA966974 CA966269 CA965237 DW719669 DW722499 DW723267 DW723915 |
| 1333 | Dr.5435 | no match | CA964221 DW722900 |
| 665 | Dr.18834 | no match | CA965343 CA965232 CA967575 DW720286 |
| 1638 | Dr.19449 | Ppr.18135 | DW723443 DW724408 |
| 1476 | Dr.161 | no match | CA964246 CA968663 DW721915 DW723695 |
| 1207 | Dr.19896 | no match | CA966601 CA965693 CA969864 DW721990 |
| 1398 | Dr.17192 | no match | CA969211 DW723295 |
| 1233 | Dr.28231 | Ppr.14705 | CA969806 CA968842 AU312483 AU301581 AU301004 AU052093 AU052077 DW720598 DW720729 DW720857 DW721033 DW721515 DW722183 DW722262 DW722661 DW722886 DW723961 |
| 1584 | Dr.7200 | no match | CF660421 CF660359 CA968746 DW724187 |
| 374 | Dr.25678 | no match | CF660970 CF660727 CF660396 CF662960 CF662244 CA966692 CA966390 CA966361 CA965140 CA964298 CA970308 CA968336 CA968300 DW724121 |
| 1615 | Dr.20153 | no match | CA965179 CA965131 CA970301 CA967872 DW720988 DW724301 |
| 1306 | Dr.29698 | no match | CA964892 CA968808 CA968735 DW722743 |
| 1162 | Dr.888 | no match | CA969743 CA969640 AU062364 DW721441 |
| 649 | Dr.15045 | no match | CA967647 DW719780 |
| 1572 | Dr.22620 | no match | CA966074 CA967598 DW724155 |
| 1430 | Dr.24774 | no match | CF662799 CA967038 CA968409 AU301088 DW719457 DW723023 DW723467 DW723680 DW723762 DW723960 |
| 1145 | Dr.907 | Ppr.12903 | CF661670 CF661637 CA965332 CA967418 CA967352 AU183418 DW720844 DW720931 DW720993 DW721057 DW721245 DW721475 DW721513 DW721706 DW721809 DW721953 DW722269 DW722500 DW723636 DW723900 |
| 382 | Dr.34631 | no match | CF661796 CF662340 CF662337 CF662335 CF662332 CA967307 CA966870 CA965301 CA965294 CA965290 CA965289 CA965284 CA965276 CA965274 CA965273 CA965269 CA970254 CA970222 CA970221 DW720487 DW722064 DW722196 DW723059 DW723362 |
| 337 | Dr.6073 | no match | CA964391 DW719467 |
| 1318 | Dr.15054 | no match | CF662129 CF662123 CF661937 CF662543 DW720173 DW720986 DW721125 DW721247 DW721575 DW721589 DW722325 DW722814 |
| 1091 | Dr.922 | no match | DW720063 DW720072 |
| 340 | Dr.1368 | Ppr.3339 | CF661929 CF661544 CA967143 CA967106 CA965935 CA965321 CA964366 AU301056 DW721165 DW721795 DW721796 DW721988 DW722783 DW722988 DW723347 DW723359 DW724308 |
| 1329 | Dr.3275 | no match | CF662550 CA968577 DW722870 |
| 571 | Dr.1246 | no match | CF661464 CF661090 CF661019 CF660546 CA966710 CA964343 CA964044 CA968317 CA967421 CA967419 DW721198 DW723658 |
| 1214 | Dr.582 | no match | CA966282 DW721239 DW722030 |
| 1190 | Dr.1079 | no match | CF662281 CA965236 CA970358 CA967379 DW721800 |
| 1589 | Dr.12551 | no match | CA964261 CA964256 CA964162 DW724214 |
| 1472 | Dr.3523 | no match | CF662760 DW723678 |
| 1573 | Dr.32774 | no match | CF662961 CA966505 CA965182 CA968342 CA967839 DW724157 |
| 1098 | Dr.12423 | no match | DW720097 DW720743 DW720108 |
| 1609 | Dr.46790 | no match | CA966105 DW723825 DW724267 |
| 1059 | Dr.18459 | no match | CA965272 DW720160 DW720525 |
| 1080 | Dr.22853 | no match | DW719372 DW719967 |
| 1171 | Dr.31321 | Ppr.13202 | CA966006 CA964550 AU301112 AU301042 AU279271 DW721590 DW721714 DW721742 DW721831 DW721870 DW721906 |
| 1345 | Dr.2860 | no match | CF662012 CA967165 CA969711 CA969544 DW722645 DW722951 |
| 1281 | Dr.6419 | no match | CF660415 CA967648 DW722586 |
| 1083 | Dr.1756 | Ppr.17170 | DW719476 DW719992 |
| 1113 | Dr.7484 | Ppr.6941 | CA969591 CA969145 DW720855 |
| 1110 | Dr.5705 | no match | CF661755 CF661484 DW720835 |
| 674 | Dr.8505 | no match | CF662540 CA966854 CA966418 CA965469 CA967546 CA967520 CA967504 CA967480 CA967457 CA967435 DW720495 DW720588 |
| 1317 | Dr.12423 | no match | DW721109 DW722811 |
| 1322 | Dr.1081 | no match | AU183463 AF001098 DW720490 DW721525 DW722220 DW722841 DW723346 DW723372 |
| 1603 | Dr.31066 | no match | CA966545 CA964860 CA968853 CA968367 AF170296 DW723803 DW724253 |
| 1357 | Dr.38139 | no match | CA967119 DW722696 DW723017 |
| 1503 | Dr.1357 | no match | CA965171 DW723838 |
| 1189 | Dr.9515 | no match | AU062354 DW721792 |
| 1518 | Dr.14147 | no match | DW719811 DW719839 DW723889 |
| 1152 | Dr.889 | no match | CA965977 CA964168 DW721386 |
| 1127 | Dr.28199 | no match | CA965998 CA965012 CA968817 CO729410 AU301108 DW719833 DW721016 DW721671 DW722280 DW723511 |
| 1537 | Dr.6237 | no match | CA969322 CA968103 AU301113 AU081459 AU052027 DW721469 DW723977 |
| 1509 | Dr.9809 | no match | CA965052 DW723863 |
| 1416 | Dr.7977 | no match | CF661844 CA965886 CA969230 CA968094 AU052030 DW722777 DW723375 |
| 1608 | Dr.610 | no match | AF170295 DW724266 |
| 1298 | Dr.1372 | no match | CF660366 CF662357 DW722719 |
| 1131 | Dr.9707 | Ppr.13565 | CF661837 DW721065 |
| 1325 | Dr.1323 | no match | CF662214 CF662036 CF662000 CF661997 CF661893 CF661880 CF661775 CF661747 CF661697 CF661663 CF661656 CF661595 CF661141 CF662424 CA966353 CA965091 CA964819 CA964799 CA970315 CA970244 CA968822 CA967447 CA967330 AU312552 AU312511 AU312478 AU301019 AU300969 AU240365 AU183539 AU183521 AU183370 AU183354 AF528156 AB004739 AB004738 AB004737 AB004736 AB004735 AB004734 M25643 AlphaGlobin DW720120 DW720187 DW720264 DW720270 DW720275 DW720300 DW720371 DW720429 DW720497 DW720520 DW719866 DW720624 DW720631 DW720634 DW720665 DW720668 DW720684 DW720692 DW720696 DW720700 DW720715 DW720719 DW720732 DW720748 DW720768 DW720828 DW720843 DW720874 DW720911 DW720954 DW721051 DW721053 DW721145 DW721270 DW721381 DW721406 DW721496 DW721518 DW721550 DW721583 DW721716 DW721771 DW721804 DW721819 DW721862 DW721939 DW722040 DW722058 DW722059 DW722092 DW722113 DW722129 DW722130 DW722158 DW722230 DW722279 DW722399 DW722437 DW722447 DW722450 DW722498 DW722539 DW722544 DW722562 DW722582 DW722590 DW722599 DW722621 DW722664 DW722672 DW722673 DW722675 DW722699 DW722701 DW722730 DW722742 DW722771 DW722858 DW722869 DW722872 DW722873 DW722896 DW722902 DW722912 DW722929 DW722933 DW722979 DW722982 DW723031 DW723042 DW723049 DW723056 DW723098 DW723100 DW723120 DW723123 DW723143 DW723151 DW723167 DW723188 DW723194 DW723208 DW723220 DW723221 DW723225 DW723227 DW723277 DW723281 DW723283 DW723293 DW723305 DW723321 DW723325 DW723329 DW723345 DW723357 DW723371 DW723376 DW723384 DW723409 DW723424 DW723431 DW723434 DW723446 DW723491 DW723507 DW723518 DW723529 DW723555 DW723565 DW723566 DW723567 DW723584 DW723618 DW723638 DW723640 DW723644 DW723646 DW723651 DW723675 DW723676 DW723679 DW723689 DW723812 DW723851 DW723862 DW723978 DW724143 |
| 1643 | Dr.743 | no match | CF662006 DW724340 DW724421 |
| 412 | Dr.51559 | Ppr.13216 | CF660506 CA966805 CA966780 CA965730 CA965622 CA969799 DW723481 |
| 1565 | Dr.36768 | no match | CA968229 DW724122 |
| 1308 | Dr.20052 | Ppr.13027 | CA966741 CA967686 AU301606 AU301179 DW722000 DW722776 |
| 1479 | Dr.16126 | no match | DW722597 DW723721 |
| 1429 | Dr.6924 | no match | CF660950 CF662513 CA967082 CA965379 CA963999 DW723461 |
| 1359 | Dr.44282 | no match | CF662272 DW723051 |
| 594 | Dr.4212 | no match | CF661989 CF660442 CF662629 CA967039 CA967004 CA966733 CA966634 CA968022 AF076528 DW722968 |
| 1216 | Dr.1126 | no match | CF661682 CF660466 CA970429 AU312560 AU301571 AU300987 AU183480 AU062382 AU062356 DW719588 DW721553 DW721623 DW722072 DW722529 DW723202 DW724201 |
| 801 | Dr.44399 | no match | CF662581 CA970432 AU183447 DW721719 |
| 985 | Dr.17679 | Ppr.689 | DW719466 DW722853 |
| 1635 | Dr.46983 | no match | CF662147 CF661927 CF661888 CF661845 CF661819 CF661774 CF661753 CA964307 CA970267 AU301226 AU301053 AU301021 AU183422 AU183381 AU183348 DW720268 DW720820 DW720847 DW720888 DW720956 DW720968 DW721226 DW721282 DW721377 DW721450 DW721500 DW721501 DW721548 DW721655 DW721768 DW721803 DW721818 DW721902 DW722025 DW722063 DW722281 DW722359 DW722479 DW722692 DW722735 DW722895 DW722977 DW723076 DW723333 DW723351 DW723414 DW723572 DW723604 DW723701 DW723728 DW723795 DW724389 |
| 1135 | Dr.1345 | no match | CF662171 CF662010 CF661488 CA967596 DW721117 DW722312 DW722862 DW722961 DW723217 |
| 754 | Dr.1161 | Ppr.3504 | CF661285 CF660949 CF660866 CF662935 CA967226 CA966899 CA970319 CA968752 AU301632 AU301192 AU301011 AU279301 DW722725 |
| 1513 | Dr.2483 | no match | CA966903 CA966827 DW723874 |
| 1203 | Dr.20097 | no match | DW720846 DW721048 DW721853 DW721914 DW721950 |
| 1374 | Dr.28230 | no match | CF662074 CF662073 CF661920 CF661877 CF661872 CF661803 CF661759 AU301020 DW720632 DW721192 DW721482 DW722332 DW722428 DW723126 |
| 1502 | Dr.6314 | no match | CA967015 CA969803 AJ492825 DW723833 |
| 1425 | Dr.30171 | no match | CA967818 DW720905 DW723433 |
| 747 | Dr.6496 | Ppr.1060 | CF661084 CF660582 CA966612 CF662218 CA967138 CA967121 CA965879 CA965647 CA964545 CA970343 CA970317 CA969699 AU301200 AU081438 AU062377 AU062369 C88364 DW719620 DW720183 DW720057 DW721068 DW721489 DW722270 DW722403 DW722596 DW723052 DW723469 |
| 1101 | Dr.559 | no match | AF479820 DW719454 DW719563 DW719727 DW719784 DW719802 DW720008 DW720085 DW720107 |
| 1166 | Dr.1434 | no match | CA964027 AU300929 DW721208 DW721516 |
| 1252 | Dr.32238 | no match | CA968612 DW722307 |
| 1354 | Dr.31440 | Ppr.1123 | AU279327 DW721888 DW723007 |
| 1563 | Dr.26855 | Ppr.15234 | CF662453 CF662426 DW724098 |
| 1378 | Dr.633 | no match | CF661817 CA970235 CA968613 AU312518 AU301097 DW721142 DW721980 DW723164 |
| 1462 | Dr.16677 | no match | DW723598 DW723611 |
| 1003 | Dr.20690 | no match | DW719604 DW719835 |
| 1025 | Dr.51174 | no match | DW719595 DW719803 |
| 1054 | Dr.29757 | no match | CF662468 CF662457 CF662417 CF662395 CF662251 CA966377 CA965448 CA965443 CA965424 CA965389 CA965309 CA965308 CA965302 CA965196 CA965188 CA970414 CA968789 CA967524 CA967514 CA967350 AY225965 AY225964 DW720486 |
| 1015 | Dr.12497 | no match | DW719533 DW719676 |
| 976 | Dr.17933 | no match | DW719392 DW720915 |
| 1427 | Dr.1340 | no match | AU301051 AU279344 DW719889 DW721490 DW721556 DW722070 DW722356 DW723439 |
| 1048 | Dr.28214 | no match | CA966380 AU301043 AU052101 DW720170 DW720385 DW720415 |
| 1278 | Dr.962 | Ppr.1283 | CA966884 CA966880 DW722540 |
| 1484 | Dr.28839 | no match | CF662194 CF662108 CF661856 CA966093 CA966000 CA968588 CA970457 AU301081 AU183520 AU183384 AU052089 DW720104 DW721220 DW721471 DW721757 DW722128 DW722259 DW722260 DW722264 DW722566 DW722779 DW723147 DW723736 |
| 1000 | Dr.3315 | no match | CF662233 DW719579 |
| 1381 | Dr.20495 | no match | CA966581 DW723187 |
| 1061 | Dr.31821 | no match | CF662004 DW720547 |
| 1029 | Dr.11003 | no match | DW719842 DW719970 |
| 1057 | Dr.4190 | Ppr.14494 | CF660940 CA964903 DW720356 DW720507 |
| 1634 | Dr.13015 | no match | CA964661 DW720324 DW720592 DW722400 DW723113 DW723301 DW724386 |
| 538 | Dr.17520 | no match | CA968630 DW719606 |
| 1270 | Dr.11722 | no match | CA965923 CA968873 CA967781 DW722461 |
| 1555 | Dr.1351 | no match | CF662024 DW721182 DW721647 DW722720 DW722799 DW723742 DW724075 |
| 1389 | Dr.30839 | no match | DW722588 DW722927 DW723234 |
| 1375 | Dr.20602 | no match | CA964320 DW720919 DW723131 |
| 1460 | Dr.28592 | no match | CA965859 DW723183 DW723606 |
| 1332 | Dr.11351 | no match | CA964032 DW722898 |
| 244 | Dr.27162 | no match | CA965784 DW720226 |
| 1585 | Dr.4765 | no match | CA969528 DW724188 |
| 1623 | Dr.1199 | Ppr.17122 | CA964234 DW724325 DW724331 |
| 1441 | Dr.37248 | Ppr.14943 | DW722786 DW723516 |
| 1399 | Dr.28413 | no match | DW720870 DW721910 DW722154 DW723012 DW723297 DW723474 |
| 1206 | Dr.30820 | no match | CF661938 CA966966 AU312494 AU052066 DW719675 DW720891 DW721431 DW721493 DW721638 DW721978 DW722006 DW722039 DW722116 DW722298 DW722317 DW722668 DW723004 DW723152 |
| 986 | Dr.36542 | Ppr.12788 | DW719475 DW719479 |
| 999 | Dr.36802 | no match | CF661104 CF661065 DW719578 |
| 1289 | Dr.19474 | no match | DW722360 DW722637 |
| 1180 | Dr.15857 | no match | CF661432 DW721721 |
| 1232 | Dr.24923 | no match | DW720802 DW721755 DW722088 DW722177 DW722302 DW722534 DW723683 DW723845 DW724117 DW724288 |
| 1012 | Dr.473 | no match | AB009406 AB009405 AB009404 DW719667 |
| 1008 | Dr.14434 | no match | DW719632 DW720051 |
| 835 | Dr.3131 | Ppr.14880 | CF662182 CA964757 CA964241 AU183407 AU052112 AU052097 DW719460 DW721162 DW721209 DW721569 DW721823 DW721903 DW722577 DW723168 DW723200 DW723617 DW723853 DW723910 |
| 1498 | Dr.29172 | no match | CF663121 CF663031 CF662459 CF662358 CA965127 CA964148 CA967319 AU301215 AU279352 DW721478 DW721995 DW723823 |
| 447 | Dr.31740 | no match | CA969381 DW719822 |
| 1607 | Dr.1432 | no match | CF662213 CF662093 CF661924 CF661788 CF661787 AU301014 AU240355 AU052085 DW719378 DW720867 DW721093 DW721485 DW721645 DW721781 DW722345 DW722834 DW723341 DW723526 DW724197 DW724265 |
| 379 | Dr.37835 | no match | CA966938 CF662192 CF662176 CF662102 CA966970 CF662030 CF661922 CA967167 CF661902 CF661879 CF661864 CF661862 CF661851 CF661811 CF661805 CF661801 CA966968 CF661729 CF661717 CA966926 CF661683 CF661681 CF661665 CF661634 CF661618 CF661566 CF661494 CA967164 CA967118 CA964471 CA964201 CA964131 CA970269 CA970258 CA970239 CA970183 CA968859 CA968711 CA968633 CA968592 CO729412 AU301712 AU301609 AU301586 AU183435 AU183424 AU183378 AU183343 AU062378 DW720924 DW721045 DW721060 DW721171 DW721187 DW721373 DW721455 DW721465 DW721512 DW721554 DW721572 DW721574 DW721637 DW721756 DW721840 DW721965 DW722002 DW722056 DW722090 DW722091 DW722218 DW722246 DW722263 DW722275 DW722372 DW722383 DW722409 DW722475 DW722483 DW722633 DW722948 DW723038 DW723062 DW723110 DW723161 DW723410 DW723441 DW723609 DW723612 DW723879 DW723903 DW724024 DW724040 DW724190 DW724226 DW724272 DW724343 DW724378 |
| 1311 | Dr.17186 | no match | CA967325 DW722782 |
| 1401 | Dr.4835 | no match | CF660896 DW723311 |
| 1044 | Dr.28529 | no match | CA966421 CA965932 CA967727 DW720350 |
| 1315 | Dr.4724 | no match | CA968393 CA967907 DW722037 DW722806 |
| 1130 | Dr.30264 | Ppr.3081 | CF661126 CA966900 CF660874 CF660420 CA966134 CA967392 AU183409 DW720992 DW721058 DW721765 DW721867 DW723819 DW723979 |
| 1639 | Dr.632 | no match | CF661860 DW722980 DW723664 DW723950 DW724409 |
| 1139 | Dr.1347 | no match | CF660578 CF663052 CA967873 AU312532 AU301077 AU183535 AU183471 DW720943 DW721157 DW721389 DW722255 DW722658 DW723426 DW723539 |
| 1035 | Dr.33850 | no match | DW720228 DW720559 |
| 1394 | Dr.1370 | no match | CA965384 CA967516 C88406 DW723268 |
| 1319 | Dr.19525 | no match | CA966333 DW722824 |
| 1350 | Dr.52760 | no match | DW721034 DW721498 DW721722 DW722969 |
| 612 | Dr.30332 | no match | CF661757 CA964151 CA969456 CA967838 CA967741 AU301183 AU183484 AU052108 DW719442 DW721054 DW721096 DW722878 |
| 1292 | Dr.4272 | no match | CA966367 DW722652 |
| 1356 | Dr.7713 | Ppr.12374 | CF663037 CA966628 CA965516 DW723010 |
| 1419 | Dr.28173 | no match | CF663102 CA969427 DW723400 |
| 1222 | Dr.959 | no match | CF662371 CA969378 CA967915 DW721207 DW722120 |
| 1277 | Dr.31756 | no match | CF662166 CF661635 CA964070 CA970284 DW720853 DW721028 DW721432 DW722537 |
| 1240 | Dr.1043 | no match | CA967156 CA967155 CA966765 CA965972 AB078926 DW719615 DW719645 DW720877 DW721829 DW722156 DW722211 DW722374 DW723020 DW723466 DW724099 DW724198 |
| 1086 | Dr.32035 | no match | DW720032 DW720048 |
| 1056 | Dr.36424 | no match | CF661100 DW720502 |
| 1353 | Dr.40117 | no match | CA967195 CA968699 AU300988 DW720894 DW720973 DW721627 DW721650 DW722273 DW722670 DW722856 DW723002 |
| 1618 | Dr.1382 | no match | DW722992 DW723407 DW724309 |
| 1022 | Dr.24208 | no match | CA966450 CA965822 DW719777 |
| 1458 | Dr.51771 | Ppr.1194 | DW719477 DW719505 DW719601 DW719640 DW719828 DW723600 |
| 1571 | Dr.8724 | no match | CF660574 CF660491 CA965342 DW724150 |
| 1219 | Dr.2945 | no match | CF662065 CF662002 CF661874 CF661570 CF661431 CF661425 CA966655 CF661037 CF660959 CF660738 CF660610 CF662569 CF662359 CA967248 CA967084 CA966975 CA966954 CA966356 CA966120 CA965505 CA965438 CA965421 CA965413 CA965400 CA965394 CA965371 CA965356 CA965351 CA965311 CA965304 CA965291 CA965260 CA965242 CA965217 CA965210 CA965209 CA965207 CA965175 CA965161 CA965160 CA965156 CA965155 CA964800 CA964754 CA964475 CA964163 CA964079 CA967591 CA967554 CA967542 CA967531 CA967521 CA967467 CA970430 CA967424 CA967374 CA967556 AU312496 AU312484 AU301216 AU279242 AU052091 AU052043 DW720121 DW720123 DW720131 DW720136 DW720146 DW720156 DW720162 DW720166 DW720167 DW720174 DW720177 DW720190 DW720200 DW720204 DW720208 DW720211 DW720216 DW720218 DW720229 DW720259 DW720269 DW720273 DW720274 DW720278 DW720282 DW720296 DW720297 DW720304 DW720310 DW720313 DW720315 DW720323 DW720332 DW720352 DW720353 DW720365 DW720373 DW720383 DW720396 DW720410 DW720411 DW720412 DW720425 DW720427 DW720434 DW720435 DW720436 DW720451 DW720455 DW720467 DW720475 DW720491 DW720492 DW720500 DW720504 DW720508 DW720514 DW720515 DW720516 DW720519 DW720523 DW720531 DW720534 DW720535 DW720539 DW720541 DW720545 DW720550 DW720557 DW720561 DW720565 DW720572 DW720573 DW720587 DW720594 DW720596 DW720599 DW720606 DW719850 DW719853 DW719864 DW719868 DW720622 DW720625 DW720628 DW720633 DW720636 DW720649 DW720651 DW720667 DW720675 DW720686 DW720687 DW720688 DW720693 DW720698 DW720703 DW720705 DW720706 DW720711 DW720718 DW720723 DW720724 DW720733 DW720750 DW720752 DW720753 DW720754 DW720757 DW720758 DW720759 DW720770 DW720771 DW720776 DW720779 DW720783 DW720808 DW720822 DW720916 DW721062 DW721232 DW721619 DW721774 DW721955 DW721958 DW721977 DW721993 DW722087 DW722127 DW722145 DW722186 DW722250 DW722454 DW722530 DW722547 DW722553 DW722601 DW722679 DW722750 DW722876 DW723142 DW723219 DW723510 DW723548 DW723559 DW723767 DW723916 DW724320 |
| 1552 | Dr.1116 | no match | CA966198 DW722781 DW724066 |
| 1560 | Dr.15151 | no match | CF661108 DW724094 |
| 1473 | Dr.27166 | no match | CF662454 CF662441 CF662356 CF662259 CF662237 CA965411 CA965404 DW723682 |
| 1628 | Dr.33746 | no match | DW724280 DW724346 |
| 1451 | Dr.2536 | no match | CA967262 DW723583 |
| 1002 | Dr.13282 | no match | DW719585 DW719709 |
| 1341 | Dr.32020 | no match | DW720972 DW722924 |
| 1084 | Dr.25213 | no match | CA964543 CA964370 CA968646 AU312512 AU301068 AU052054 AU052034 DW720196 DW719993 DW720681 DW721169 DW721240 DW721383 DW721629 DW721631 DW721698 DW721700 DW721929 DW722073 DW722514 DW722913 DW722985 DW723015 DW723235 DW723533 DW723922 DW723925 DW724395 |
| 1530 | Dr.15836 | no match | DW721453 DW723941 |
| 1636 | Dr.321 | Ppr.18406 | CF660598 CF663033 CA964893 CA967393 DW720026 DW724392 |
| 1144 | Dr.48326 | no match | CF662096 CA968785 AU301057 AU279265 DW720823 DW720825 DW721238 DW721788 DW722894 DW723674 |
| 1483 | Dr.30882 | no match | CA967150 CA967124 AY949988 DW723735 |
| 1522 | Dr.1067 | Ppr.745 | CA966596 CA966575 CA964438 CA969392 CA968908 CA968358 C88394 DW723907 |
| 1534 | Dr.24921 | no match | CF660375 CA965907 CA969582 DW723956 |
| 1097 | Dr.16440 | no match | DW720096 DW720106 |
| 1197 | Dr.19225 | no match | CA967202 CA966943 CA968423 CF663001 CF662776 CF662708 CF662701 CA967288 CA967131 CA966808 CA966699 CA966665 CA966609 CA966608 CA966472 CA966358 CA965119 CA965001 CA964439 CA964404 CA964377 CA964275 CA970359 CA970356 CA969837 CA969585 CA969260 CA968369 CA968277 CA968236 AU062368 DW721848 |
| 1580 | Dr.7311 | no match | CA964852 DW724174 |
| 576 | Dr.20805 | no match | CA968232 DW719817 |
| 1347 | Dr.28202 | Ppr.18340 | CF662934 CA964279 CA968888 DW721868 DW722956 |
| 1258 | Dr.85 | no match | CA965610 CA968990 DW722371 |
| 1504 | Dr.10241 | no match | DW720043 DW722467 DW723840 |
| 1440 | Dr.42359 | no match | DW723258 DW723504 |
| 1415 | Dr.28864 | no match | CF661756 CF661495 CF660939 CF662926 CF662556 CF662541 CF662440 CA967221 CA965280 CA965246 CA964004 CA968485 CA967742 L27172 L08689 DW720122 DW720129 DW720157 DW720203 DW720206 DW720212 DW720450 DW720848 DW721180 DW721696 DW721865 DW721923 DW722192 DW722304 DW723373 |
| 1075 | Dr.18551 | Ppr.18198 | DW719875 DW719907 DW719923 DW720091 |
| 1078 | Dr.2813 | no match | DW719885 DW719935 DW722796 |
| 1243 | Dr.28318 | no match | CA970249 DW722153 DW722223 |
| 1384 | Dr.48615 | no match | CF662078 CF661953 DW723198 |
| 1036 | Dr.1109 | no match | CF661721 CF661620 CF661545 CF661433 CF661067 CF660405 CF662439 CA966863 CA966650 CA966048 CA966045 CA965313 CA964252 CA968768 CA968449 CA967831 AU300972 AU279324 AU279294 AU279262 AU052038 M24113 DW720144 DW720184 DW720236 DW720245 DW720271 DW720299 DW720302 DW720342 DW720351 DW720357 DW720360 DW720382 DW720390 DW720402 DW720409 DW720422 DW720428 DW720459 DW720489 DW720533 DW720544 DW720579 DW720597 DW720619 DW720620 DW720716 DW720762 DW720763 DW720765 DW720812 DW720833 DW721077 DW721161 DW721205 DW721543 DW721640 DW721659 DW721685 DW721728 DW721729 DW721871 DW721944 DW721961 DW721962 DW722003 DW722013 DW722044 DW722170 DW722319 DW722515 DW722575 DW722864 DW723033 DW723134 DW723154 DW723246 DW723319 DW723326 DW723336 DW724207 DW724275 |
| 1471 | Dr.31614 | no match | CF661690 CA967210 CA967306 AY249415 DW721038 DW721097 DW721152 DW721858 DW722682 DW722972 DW723670 |
| 1468 | Dr.30814 | no match | CF662156 CF661642 CF661502 CF662662 CA966378 CA964815 CA964166 CA968799 CA968705 CA968704 CA970451 CA967638 AU312514 AU312505 AU301621 AU183526 AU183511 AU183462 AU052075 DW721236 DW721405 DW721932 DW722638 DW723122 DW723335 DW723657 |
| 1265 | Dr.26956 | no match | CF662188 CF662177 CF661840 CA964855 CA970425 CA968823 CA968820 CA968594 CA968593 CA967670 AU052095 DW721430 DW721524 DW722234 DW722335 DW722413 DW723166 DW723688 DW723719 DW724102 DW724202 |
| 1321 | Dr.23079 | no match | CF660842 DW722840 |
| 1365 | Dr.1320 | no match | CA964203 AU279381 C88367 DW721223 DW721492 DW722242 DW723048 DW723094 |
| 1385 | Dr.11042 | no match | DW721799 DW723203 |
| 1407 | Dr.1566 | Ppr.17085 | CA964773 CA968782 CA968318 DW721976 DW723332 |
| 1512 | Dr.3270 | Ppr.17360 | CF661020 DW723871 |
| 1526 | Dr.28184 | no match | CA969703 DW721689 DW722435 DW723928 |
| 1517 | Dr.30259 | Ppr.14819 | CA969261 DW723886 |
| 1196 | Dr.39 | no match | CF660999 CF662999 CF662350 CA964379 AU081428 DW721830 |
| 1464 | Dr.5594 | no match | CF662971 CF662970 CF662707 CF662636 CF662312 CA967264 DW723632 |
| 1085 | Dr.44354 | Ppr.996 | DW719403 DW719499 DW720015 |
| 813 | Dr.667 | no match | AU081500 AU081491 AU081467 DW722965 DW723386 |
| 269 | Dr.18315 | no match | CF661179 CA967083 CA966738 CA965482 CA965388 AU183359 DW721024 DW723828 |
| 1403 | Dr.1310 | Ppr.1008 | DW722376 DW723316 |
| 1316 | Dr.2045 | no match | CF662461 CA967363 DW722810 |
| 1228 | Dr.1339 | no match | CF662068 CF661096 AU312480 AU279298 AU052110 AB012087 DW721031 DW722166 DW722179 DW722310 DW723702 DW723790 |
| 1576 | Dr.1142 | no match | CA969976 DW724162 |
| 1051 | Dr.31376 | no match | CA965420 CA965410 CA965286 CA965192 CA970438 CA967417 AB052623 DW720456 DW720836 DW721620 |
| 997 | Dr.1428 | no match | CF662184 CF662023 CF661791 CA964841 AU301005 AU062375 DW719573 |
| 1418 | Dr.28962 | no match | DW719746 DW723254 DW723381 |
| 1497 | Dr.27165 | no match | DW719901 DW721938 DW723817 DW723821 |
| 1102 | Dr.18933 | no match | CA965323 CA965251 CA964793 CA968897 DW720126 DW720133 DW720188 DW720339 DW720377 DW720506 DW720529 DW720558 DW720666 DW720780 DW720792 |
| 1622 | Dr.14110 | no match | CA968676 CA968660 DW721875 DW721912 DW722236 DW722608 DW723240 DW724327 |
| 1260 | Dr.30177 | no match | DW722208 DW722266 DW722320 DW722381 |
| 1450 | Dr.1310 | Ppr.1008 | CA970431 AU301588 AU300942 AU183393 DW721116 DW722214 DW723387 DW723462 DW723582 |
| 1368 | Dr.31916 | no match | CA966292 DW723097 |
| 1211 | Dr.30372 | no match | CF662600 DW722021 |
| 1449 | Dr.13517 | no match | DW719696 DW722970 DW723580 |
| 711 | Dr.26403 | no match | CF661658 CA964817 CA964765 CA964533 CA967878 CO729438 AU312492 AU301003 AU183523 AU183477 AU183395 AU081402 AU052065 DW721183 DW721658 DW721727 DW721850 DW722077 DW722198 DW722365 DW723669 DW723757 DW724133 DW724277 DW724329 |
| 975 | Dr.13168 | no match | DW719389 DW719391 |
| 1254 | Dr.39206 | Ppr.17847 | CA966769 CA965104 CA968016 DW722323 DW724415 |
| 1593 | Dr.3417 | no match | CA966963 CA966924 CA966922 AU301651 DW722693 DW724223 |
| 1542 | Dr.13426 | no match | CA967690 DW724020 |
| 1129 | Dr.28842 | Ppr.6905 | CF663067 CF662669 CA966919 CA966918 AU240360 AU240359 DW721052 DW721158 |
| 1234 | Dr.2718 | no match | CA966927 DW722188 |
| 1033 | Dr.28455 | no match | CF661745 CF661586 CF661543 CF661539 CF661510 CF661503 CF661486 CF661462 CF661449 CF661439 CF661427 CF661036 CF661002 CF660941 CF660920 CF660908 CF660894 CF660847 CF660771 CF660631 CA966067 CA965504 CA965326 CA965183 CA964830 CA964791 CA964204 CA964122 CA970280 CA968863 CA970458 CA967660 CA967585 CA967411 CA967405 CA967348 CA967331 CA967322 AJ308993 U88562 DW720135 DW720152 DW720155 DW720176 DW720217 DW720219 DW720237 DW720246 DW720260 DW720265 DW720279 DW720283 DW720311 DW720325 DW720334 DW720336 DW720372 DW720375 DW720387 DW720389 DW720391 DW720417 DW720457 DW720458 DW720463 DW720472 DW720474 DW720485 DW720494 DW720499 DW720505 DW720512 DW720542 DW720566 DW720577 DW720585 DW720691 DW720784 DW720785 DW720786 DW720794 DW720795 DW721244 DW721732 DW722251 |
| 1143 | Dr.12055 | no match | CF661430 CF661414 CF660984 DW721227 |
| 1591 | Dr.10136 | no match | CA964398 CA964367 CA967899 DW721099 DW724221 |
| 836 | Dr.26696 | no match | CA964562 AU183372 AU052111 AU052028 DW721193 DW722397 |
| 1355 | Dr.50702 | no match | AU052057 DW719730 DW720098 DW720109 DW723008 |
| 1428 | Dr.33938 | no match | CA969607 DW723450 |
| 1376 | Dr.14061 | no match | CF660437 DW723141 |
| 1320 | Dr.14705 | no match | DW722531 DW722828 |
| 1474 | Dr.33997 | Ppr.18241 | DW720414 DW721087 DW722051 DW722607 DW723223 DW723224 DW723531 DW723684 |
| 1026 | Dr.32371 | no match | CF662325 CA969992 DW719763 DW719815 DW724008 |
| 1261 | Dr.51020 | no match | CF662197 CF662098 CF661809 CF661405 CA967203 CA967160 CA966119 CA964262 CA964114 CA968918 CA968860 CA968838 CA968596 CA967790 CA967744 CA970443 DW722391 |
| 522 | Dr.7469 | no match | CF661936 CF661639 CF661585 CF661551 CF661027 CA965929 CA970229 CA968915 CA968806 CA967700 CA967639 AU312555 AU301100 AU052036 DW720149 DW720159 DW720169 DW720181 DW720193 DW720198 DW720404 DW720453 DW720517 DW720526 DW720536 DW720553 DW720605 DW719872 DW720039 DW720643 DW720671 DW720708 DW720726 DW720755 DW720756 DW720777 DW720865 DW721176 DW721385 DW721479 DW722423 DW722452 DW722458 DW723420 DW723440 DW724321 |
| 1632 | Dr.29045 | no match | CF660514 CA966673 CA966633 CA966066 CA964403 CA964347 AU279293 DW720934 DW721690 DW724250 DW724376 |
| 1541 | Dr.30277 | no match | DW721458 DW722199 DW722807 DW724013 |
| 1423 | Dr.36750 | no match | CA965910 CA968874 DW723417 |
| 1018 | Dr.15469 | no match | DW719708 DW719717 |
| 1167 | Dr.7740 | no match | CF662054 CF660496 CF663045 CA966865 CA964753 CA964474 CA969485 CA969453 AU301046 AU183498 AB098609 DW721533 DW721982 |
| 1007 | Dr.1966 | no match | CA967681 DW719630 |
| 1235 | Dr.14849 | no match | CA966116 DW722189 |
| 1621 | Dr.4682 | no match | CF662641 CA965946 DW724326 |
| 1595 | Dr.9557 | no match | AU240290 DW724228 |
| 1561 | Dr.24952 | no match | CF662067 CO729414 CA968716 DW721181 DW722820 DW723732 DW724095 |
| 1188 | Dr.30553 | no match | CA966169 CA965804 CA964680 DW721769 |
| 998 | Dr.5091 | no match | DW719575 DW719904 |
| 1186 | Dr.26979 | no match | CA965203 CA967814 DW721761 |
| 582 | Dr.17536 | Ppr.11496 | CF661645 CF661155 CF662294 CF662273 CA966722 CA964336 CA969144 CA968164 CA967581 CA967377 AU240320 DW719710 DW720852 DW721415 DW722272 DW722667 DW723655 DW724172 |
| 1564 | Dr.12853 | no match | CA964759 CA967799 DW724118 |
| 243 | Dr.33610 | no match | CF662745 CA965873 DW719740 |
| 1011 | Dr.9860 | no match | CA964750 DW719648 |
| 1545 | Dr.1162 | no match | CF662739 DW724036 |
| 1343 | Dr.1051 | no match | CA966707 CF660616 CA964574 CO729436 CA967299 DW722931 |
| 981 | Dr.9622 | no match | CA967132 CA966384 AU300986 DW719445 |
| 1581 | Dr.29117 | no match | AU279365 AU052073 DW721139 DW721780 DW722703 DW722775 DW724177 |
| 1604 | Dr.9794 | no match | DW719726 DW724089 DW724254 |
| 1507 | Dr.1375 | Ppr.15427 | CA966407 CA965538 DW723852 |
| 1161 | Dr.39083 | no match | CA969197 CA969188 DW721429 |
| 1058 | Dr.1754 | no match | CA964237 CA968721 CA967612 DW720397 DW720522 |
| 1019 | Dr.2110 | Ppr.4463 | DW719705 DW719736 |
| 987 | Dr.4339 | no match | CA964250 DW719489 |
| 1380 | Dr.39199 | no match | CA964993 DW721190 DW723179 |
| 1324 | Dr.845 | no match | CF661815 CF660875 CF662498 CF662338 CA967193 CA965288 CA965264 CA964059 CA963988 CA970387 CA969950 CA968760 CA967562 AU240356 AU183396 AY167421 DW720132 DW720139 DW720150 DW720202 DW720210 DW720213 DW720215 DW720243 DW720267 DW720308 DW720326 DW720328 DW720349 DW720358 DW720370 DW720392 DW720420 DW720445 DW720464 DW720468 DW720470 DW720479 DW720549 DW720551 DW720552 DW720570 DW720600 DW720602 DW720613 DW720615 DW719869 DW720630 DW720638 DW720644 DW720645 DW720647 DW720656 DW720661 DW720670 DW720695 DW720710 DW720727 DW720730 DW720745 DW720764 DW720769 DW720910 DW721528 DW721558 DW721762 DW722286 DW722322 DW722445 DW722855 DW723101 DW723327 DW723743 |
| 990 | Dr.35189 | Ppr.2350 | AU301648 DW719514 |
| 1010 | Dr.5929 | no match | DW719637 DW719932 DW720105 |
| 1556 | Dr.28850 | no match | CA966276 AU301104 AU301182 DW724080 DW724364 |
| 983 | Dr.15468 | no match | DW719374 DW719451 DW719834 |
| 1549 | Dr.11058 | no match | DW722507 DW724059 |
| 1535 | Dr.42041 | no match | DW723628 DW723964 |
| 1107 | Dr.7464 | no match | CA967166 CA967191 CA967159 CA966965 CA965958 AU301646 AU301559 AU062381 AF255354 DW720813 DW721873 DW722606 DW723383 DW724030 |
| 1213 | Dr.5521 | no match | DW720860 DW722028 |
| 1383 | Dr.5521 | Ppr.17920 | DW721711 DW721863 DW722001 DW722069 DW722459 DW723196 |
| 1093 | Dr.16284 | no match | CF661256 CF661220 CF660840 DW720650 |
| 1023 | Dr.2813 | no match | DW719788 DW719858 DW719895 |
| 1500 | Dr.2941 | no match | AF006044 CA964832 DW723829 |
| 1414 | Dr.10285 | no match | CA964372 CA964364 DW723367 |
| 1067 | Dr.47389 | no match | CF660921 DW720130 DW720201 DW720386 DW720569 DW720617 |
| 1400 | Dr.5529 | no match | CA965324 DW723306 |
| 1300 | Dr.6814 | no match | CA968672 DW719980 DW722722 |
| 1340 | Dr.28410 | no match | CA964305 DW721055 DW722663 DW722918 |
| 1248 | Dr.7876 | no match | CA966992 CA966515 CA965673 DW722267 |
| 1079 | Dr.37681 | no match | AY327474 DW719959 |
| 1388 | Dr.2897 | no match | CA966540 CA965135 CA967840 CA967573 DW723231 |
| 1195 | Dr.29643 | no match | CA970228 DW721522 DW721815 DW721821 |
| 1567 | Dr.1324 | Ppr.11863 | CF660793 CA966138 CA966102 CA964573 AU301180 DW720864 DW721642 DW721820 DW721967 DW722449 DW722611 DW723016 DW723040 DW723137 DW723229 DW723256 DW723356 DW723642 DW723764 DW724135 |
| 1063 | Dr.2426 | no match | CA965970 CA965965 CA970420 CA968831 CA968816 DW720361 DW720582 |
| 1590 | Dr.44214 | no match | CA970162 DW724216 DW724219 |
| 1295 | Dr.24991 | no match | CA966708 DW720608 DW722681 |
| 1111 | Dr.26791 | no match | CF662588 CA964211 CA968902 CA968883 CA968661 AU301107 AU301102 DW720849 DW722015 DW722016 |
| 1327 | Dr.9770 | no match | CA966972 CA966661 DW722866 |
| 1482 | Dr.52335 | no match | CF661435 DW720235 DW723727 |
| 1231 | Dr.1201 | no match | AU301616 DW720977 DW722175 |
| 1154 | Dr.28291 | no match | CA967244 CA966789 AU312520 AU052103 DW721394 |
| 1210 | Dr.876 | Ppr.222 | CA964544 CA964338 CA967310 AU279366 AU052063 DW720958 DW721488 DW722011 DW722874 |
| 1291 | Dr.9196 | no match | DW722634 DW722649 |
| 829 | Dr.3447 | no match | CF661669 CF661666 CA964910 CA964127 AU062380 DW720909 DW721090 DW721177 DW721440 DW721802 DW722825 DW723054 DW723155 DW723791 |
| 545 | Dr.2784 | no match | CA966910 CA968590 DW719586 |
| 1297 | Dr.23788 | no match | CF661873 DW720935 DW721408 DW721613 DW722714 |
| 1456 | Dr.1119 | Ppr.14737 | CA970119 DW723594 |
| 1521 | Dr.52104 | Ppr.1279 | DW719659 DW723906 |
| 1043 | Dr.44284 | no match | DW720281 DW720345 |
| 1255 | Dr.8195 | no match | L27171 DW722355 |
| 1493 | Dr.18179 | no match | DW722442 DW723794 |
| 669 | Dr.21064 | Ppr.18513 | CA965446 CA965328 CA967539 DW720293 |
| 1283 | Dr.33670 | no match | CF660900 DW722598 |
| 974 | Dr.17220 | no match | DW719381 DW719742 |
| 1082 | Dr.11310 | no match | CA970066 DW719984 |
| 1271 | Dr.383 | no match | DW721184 DW722465 |
| 1220 | Dr.1334 | no match | CF662143 CF661768 CF661712 CA964161 AU301663 AU301017 AU183406 DW720319 DW721214 DW722098 DW722342 DW722401 DW722659 |
| 1582 | Dr.29055 | no match | CF661671 AU301049 AU052062 DW721069 DW721092 DW721801 DW722388 DW723108 DW723265 DW723338 DW724178 |
| 1505 | Dr.36464 | no match | CF662870 CA966004 CA964904 CA964876 AJ544193 DW723842 |
| 1071 | Dr.33737 | Ppr.12788 | CA964749 CA968384 DW719790 DW719881 |
| 1006 | Dr.14528 | Ppr.17999 | DW719488 DW719625 |
| 1331 | Dr.31444 | no match | CA966047 CA964792 CA969634 C88371 DW722888 |
| 1149 | Dr.29735 | no match | CA970276 AU301614 DW721272 |
| 1594 | Dr.14046 | no match | DW722427 DW724225 |
| 1137 | Dr.1054 | no match | AU301063 AU300930 DW721136 |
| 996 | Dr.1438 | no match | DW719358 DW719572 DW724252 |
| 1104 | Dr.47235 | no match | CA964454 DW720116 |
| 1406 | Dr.11308 | Ppr.707 | CF660622 CA965374 CA964068 CA968257 CA967433 CA967349 CA967318 AU279370 AU183350 DW721526 DW721544 DW722288 DW723331 DW723623 DW723693 DW724424 |
| 1533 | Dr.30840 | no match | CF661799 CA967787 CA970439 AU052067 DW719973 DW721384 DW722102 DW723955 |
| 1548 | Dr.21964 | no match | DW720068 DW724050 |
| 1435 | Dr.6908 | no match | CF662398 CA964055 CA970368 DW721443 DW722994 DW723483 |
| 561 | Dr.29136 | no match | CF662198 CA966654 CF660515 CA968426 CF662989 CF662715 CA964285 CA969065 CA968400 CA968366 DW723449 |
| 1133 | Dr.3180 | no match | CA967140 CF662530 DW721079 |
| 1227 | Dr.38237 | no match | CA966828 DW722163 |
| 1118 | Dr.13635 | no match | CA966167 DW720933 |
| 1575 | Dr.2979 | no match | CF661956 AU052035 DW721212 DW723936 DW724144 DW724160 |
| 1288 | Dr.31431 | no match | CF660879 CA965466 CA964118 CA968651 CA967730 CA967636 CA967375 DW719576 DW721678 DW722635 |
| 1307 | Dr.1360 | no match | CA967380 AU301002 DW721170 DW721552 DW722754 |
| 1487 | Dr.1425 | no match | AU312479 AU301177 AU301176 AU240353 AU183351 AU052058 DW719546 DW719581 DW720967 DW720987 DW721072 DW721230 DW721889 DW721966 DW723715 DW723744 |
| 1238 | Dr.28241 | no match | CF661668 CF660726 CA967113 CA966101 CA964483 CA967436 AU312533 AU279330 AU240357 AU183345 AU183344 AU052044 DW720979 DW721666 DW721927 DW722024 DW722107 DW722182 DW722193 DW722386 DW722772 DW722773 DW722801 DW723215 DW723280 DW723608 DW723771 DW724227 DW724285 |
| 1540 | Dr.14228 | no match | CF661849 CF661556 DW723993 |
| 1559 | Dr.32371 | no match | CA966170 CA965528 CA970337 DW719383 DW723124 DW723777 DW724093 |
| 1637 | Dr.27133 | no match | CA965042 DW724379 DW724403 |
| 1606 | Dr.4096 | no match | DW719410 DW724239 DW724257 |
| 977 | Dr.35893 | no match | DW719413 DW719435 DW720035 |
| 1160 | Dr.42675 | no match | CA968881 DW721426 |
| 1120 | Dr.20805 | Ppr.15525 | AU300937 DW720975 |
| 1301 | Dr.5525 | no match | CA969227 DW722726 |
| 1475 | Dr.31777 | no match | CF661700 CA967163 CA967158 AU062370 DW723687 |
| 1532 | Dr.51949 | no match | DW723758 DW723949 |
| 1496 | Dr.25850 | no match | CA969258 CA967953 DW723811 |
| 1592 | Dr.30956 | no match | CA966562 DW724222 |
| 1229 | Dr.38330 | no match | CF662111 CF661909 CF661887 CF661804 CF661793 CF661779 CF661762 CF661736 CF661680 CF661522 CA970242 CO729445 AU279259 DW721899 DW722169 DW722651 DW722847 DW723216 DW723918 DW724371 |
| 1334 | Dr.24802 | no match | CA967042 CA965507 CA965165 AU301094 AY219845 AF003111 DW721971 DW722901 |
| 1413 | Dr.33926 | no match | CA966649 CA966124 CA964840 DW720348 DW720610 DW720642 DW723355 |
| 1122 | Dr.5545 | no match | CF661861 CF661731 CA964481 CA970295 CA968886 CA968865 CA968827 DW720996 DW721363 DW721916 DW721986 DW721989 DW722411 DW722926 DW723013 DW723252 DW723397 DW723444 DW723703 DW723848 DW723854 |
| 1431 | Dr.7856 | no match | CF661772 CA968899 DW723470 |
| 1001 | Dr.31406 | Ppr.17336 | DW719582 DW720058 |
| 1108 | Dr.24029 | Ppr.14174 | CF660918 DW720816 |
| 1489 | Dr.30250 | Ppr.5968 | CA970172 DW723779 |
| 1192 | Dr.7559 | no match | CF661596 DW721812 |
| 1212 | Dr.7105 | no match | CF660481 CA970083 CA970032 CA969738 CA969238 DW720498 DW722026 |
| 1053 | Dr.18933 | no match | DW720320 DW720483 |
| 1598 | Dr.10290 | no match | CA966430 DW719939 DW724233 |
| 980 | Dr.47489 | no match | CA968265 BK004975 DW719436 |
| 1596 | Dr.4174 | Ppr.18512 | CA966646 CA966690 CA965223 CA965199 AB042438 DW723864 DW724231 |
| 1491 | Dr.908 | no match | AU052061 DW723786 |
| 1362 | Dr.16524 | no match | AF414052 DW723032 DW723061 |
| 1209 | Dr.9060 | no match | CA964145 DW721669 DW722008 |
| 1095 | Dr.4111 | no match | CF663083 CF660457 CF662516 CF662466 CF662445 CF662437 CF662416 CF662279 CF662275 CA965224 CA967412 AF427864 DW720712 DW721563 |
| 1182 | Dr.1068 | no match | CA966444 CA969075 DW721726 |
| 1262 | Dr.13925 | no match | CA966445 CA970125 DW722396 |
| 1447 | Dr.1260 | no match | AU301072 C88439 C88389 DW720151 DW722392 DW722914 DW723560 |
| 1527 | Dr.29040 | no match | CF662018 CF661939 AU279311 AU052031 DW719758 DW719884 DW722576 DW722636 DW723930 |
| 1510 | Dr.51907 | no match | CF661323 DW723865 |
| 1371 | Dr.20715 | no match | CA969760 DW723106 |
| 1132 | Dr.30278 | no match | CA965835 CA965046 CA968267 DW721076 |
| 1094 | Dr.42611 | no match | DW720571 DW720679 |
| 1569 | Dr.14551 | no match | CA968306 CA967785 DW724147 |
| 995 | Dr.47202 | no match | CF661916 CF661906 CF661905 CF661741 CF661602 CA964197 CA964190 CA968848 AU301605 AU301604 AU279243 AU183528 AU183357 DW719571 DW720953 DW720982 DW721006 DW721155 DW721635 DW722115 DW722178 DW722425 DW722669 DW722838 DW722854 DW723270 DW723492 DW723568 DW724302 |
| 1225 | Dr.1353 | no match | CA964777 CO729440 AU301062 DW721224 DW721229 DW721491 DW722140 DW722362 DW722471 DW722945 DW723078 DW723102 DW723212 DW723378 DW723496 DW723650 DW723685 DW723958 DW724186 DW724387 |
| 547 | Dr.3568 | no match | CA965337 CA968565 DW720165 |
| 1538 | Dr.20716 | no match | CF660485 CA965113 CA969196 DW721992 DW723984 |
| 1198 | Dr.5452 | Ppr.2938 | CA967752 DW721851 |
| 1544 | Dr.10273 | no match | CF661908 CF661709 CF661450 AF255349 DW722616 DW724018 DW724026 |
| 1519 | Dr.33946 | no match | CF661784 CF661640 CA968794 AU052069 DW720845 DW721044 DW721369 DW721921 DW722078 DW722109 DW722380 DW722446 DW722559 DW723286 DW723370 DW723896 DW724292 |
| 1583 | Dr.11083 | no match | CF660460 CA966889 DW724182 |
| 1626 | Dr.10279 | no match | DW719406 DW719555 DW722117 DW724276 DW724342 |
| 1174 | Dr.23559 | Ppr.18249 | CF661591 CF660828 DW721594 |
| 1369 | Dr.29148 | no match | DW722757 DW723099 |
| 1175 | Dr.3347 | Ppr.17175 | DW719652 DW721603 |
| 1501 | Dr.30331 | no match | CA966763 CA965475 CA965247 CA964389 DW720939 DW723830 DW724130 |
| 1272 | Dr.14072 | Ppr.18124 | CA965854 DW722472 |
| 1030 | Dr.3073 | no match | CA965402 CA968710 CA967789 CA967389 CA967341 DW720134 |
| 1574 | Dr.31013 | no match | CA967852 DW724158 |
| 1579 | Dr.38337 | no match | CA966287 DW724167 |
| 549 | Dr.4905 | no match | CF662523 CF662239 CF662224 CA965382 CA968541 AY461434 DW720346 DW720443 |
| 1524 | Dr.1338 | no match | CF661904 AU300993 DW720922 DW720936 DW721022 DW721772 DW721942 DW722341 DW722343 DW723920 |
| 1047 | Dr.15332 | no match | CA964826 CA964138 DW720147 DW720405 DW720601 |
| 1236 | Dr.14283 | no match | CF662021 CF662008 CF662007 CF661882 CF661828 CF661732 CF661659 CF661654 CF661643 CA965111 CA970195 CA969940 CO729446 DW722190 |
| 1055 | Dr.36424 | no match | DW720448 DW720496 DW720543 |
| 1508 | Dr.4091 | Ppr.3278 | CF661836 CA964278 CA964123 AU312519 AU052088 AU052055 DW721770 DW722274 DW722426 DW722971 DW723815 DW723857 |
| 1128 | Dr.1368 | Ppr.3339 | CA967209 CA967206 DW721041 |
| 1205 | Dr.261 | no match | CF663016 CF662694 CF662483 CA967116 CA967098 CA967095 CA967086 CA966757 CA965136 CA970371 CA967564 CA967511 CA967345 AU312551 AU301078 AU301217 AU183527 AU183440 AU183431 AU052106 AF485331 DW721277 DW721608 DW721857 DW721952 DW722084 DW722584 DW722662 DW722740 DW723073 DW723363 DW723421 DW723947 DW724151 |
| 1287 | Dr.15867 | no match | CF661863 CF661463 CF661456 CF661125 CF663116 CA966054 DW720899 DW721002 DW721746 DW722301 DW722418 DW722618 DW722751 DW722761 DW723957 |
| 1009 | Dr.33733 | no match | CA964209 CA970378 DW719634 |
| 984 | Dr.44354 | no match | DW719455 DW720028 |
| 1155 | Dr.24810 | no match | CA968734 DW720798 DW721083 DW721104 DW721250 DW721397 DW722174 DW723034 DW723962 |
| 1176 | Dr.31854 | no match | CF662164 CF662090 CF661737 CF661718 CA969681 AU062379 DW721438 DW721607 |
| 1566 | Dr.11333 | no match | CA968421 CA966641 CA970355 DW724134 |
| 654 | Dr.1065 | no match | CF660497 CA964508 CA969316 CA970441 CA967366 AU301568 AU301026 AU301025 DW720178 DW721817 DW722501 |
| 979 | Dr.9205 | no match | CA966521 DW719416 |
| 1005 | Dr.12634 | no match | DW719623 DW720046 |
| 1159 | Dr.664 | Ppr.18332 | CF661698 CA966929 CA966809 CA970089 CA969352 CA968032 AU279289 AU052105 AU052052 DW719424 DW719433 DW719443 DW719779 DW719786 DW719837 DW719891 DW719893 DW719946 DW721081 DW721101 DW721215 DW721420 DW721693 DW724023 |
| 1520 | Dr.17883 | Ppr.8209 | CA965871 DW719512 DW723746 DW723897 |
| 1230 | Dr.4607 | no match | DW721926 DW722171 |
| 303 | Dr.1198 | no match | CA964913 DW719716 |
| 1361 | Dr.815 | no match | CF662149 CF662005 CF661733 CF661315 CF661251 CF661234 CF663114 CF660784 CF660607 CF660562 CF663103 CF660513 CF660499 CF663090 CA966689 CF660413 CF663089 CF663056 CF663049 CF663020 CF663019 CF662954 CA966866 CA966239 CA965703 CA965593 CA965565 CA965143 CA965134 CA965006 CA964917 CA964629 CA964473 CA964390 CA964352 CA964280 CA970307 CA970144 CA970126 CA970076 CA970052 CA969989 CA969948 CA969842 CA969700 CA969660 CA969646 CA969622 CA969620 CA969604 CA969554 CA969545 CA969534 CA969523 CA969438 CA969403 CA969345 CA969237 CA969235 CA969232 CA969161 CA968410 CA968391 CA968390 CA968346 CA968333 CA968099 CA968056 CA967922 CA967808 AY395871 AY395870 AY309091 D50028 DW721105 DW721561 DW722865 DW723058 |
| 614 | Dr.28451 | no match | CF662646 CF662393 CF662327 CA965316 CA965085 CA967832 DW720338 |
| 699 | Dr.16130 | no match | CF662492 CF662442 CF662423 CF662406 CF662306 CF662289 CF662249 CF662242 CF662227 CA965401 CA965230 CA968520 CA967453 CA967391 CA967311 DW720460 |
| 1115 | Dr.6650 | no match | DW720796 DW720863 |
| 1313 | Dr.43503 | no match | CA968135 DW722787 |
| 1617 | Dr.51964 | Ppr.17327 | CF662495 DW721091 DW724304 |
| 1367 | Dr.7103 | no match | CA964460 CA970265 DW723096 |
| 1323 | Dr.5438 | Ppr.17313 | AU052099 DW722844 |
| 1226 | Dr.582 | no match | DW721242 DW722141 |
| 1405 | Dr.29738 | no match | CA966645 CA966498 CA965429 CA964477 CA967586 DW723330 |
| 1455 | Dr.729 | Ppr.12930 | CF661066 CF660439 CA968812 AU301089 DW723593 |
| 1256 | Dr.30070 | no match | CF662399 CA965515 CA968500 DW722366 |
| 1465 | Dr.23788 | no match | CA964392 DW721581 DW722299 DW722737 DW723284 DW723635 |
| 1492 | Dr.8435 | Ppr.17035 | CA965884 DW723789 DW723793 |
| 1302 | Dr.428 | no match | CA964257 CA964223 DW722732 |
| 1112 | Dr.106 | no match | CA966952 DW720850 |
| 1294 | Dr.10033 | no match | CA966746 CA966455 CA966051 DW722680 |
| 826 | Dr.5555 | no match | AU081393 DW719809 |
| 1016 | Dr.26555 | Ppr.1165 | CA968036 AU312537 C88405 AB128161 DW719495 DW719682 |
| 1134 | Dr.26588 | no match | DW721082 DW721148 DW721213 DW721362 DW721421 DW721464 DW721509 DW721568 DW721617 DW721684 DW721743 DW721793 DW721909 DW721960 DW722019 DW722081 DW722104 DW722126 |
| 1344 | Dr.12425 | no match | AU301086 AU301562 DW720928 DW722937 |
| 1064 | Dr.2437 | no match | CA968755 CA967383 DW720249 DW720254 DW720329 DW720584 |
| 1170 | Dr.7610 | no match | CF662782 CA965151 CA965073 CA969068 DW721579 |
| 1426 | Dr.36479 | no match | CF661707 U83907 DW723438 |
| 972 | Dr.37232 | no match | CA969613 DW719356 |
| 1514 | Dr.1999 | no match | DW720154 DW720378 DW723880 |
| 1377 | Dr.14526 | no match | DW719987 DW723149 |
| 757 | Dr.26919 | Ppr.8271 | CF662709 CF662618 CF662597 CF662308 CA968738 CA968671 CA967630 AU312550 AU301697 AU301673 AU300936 DW721070 DW721869 DW721983 |
| 1461 | Dr.33841 | no match | DW721000 DW722614 DW723607 |
| 1156 | Dr.30368 | no match | CA965398 DW721400 |
| 9 | Dr.18507 | no match | CF662200 CF661886 CF661852 CF661850 CF661773 CF661678 CF661648 CF661587 AU300944 DW721179 DW721461 DW721585 DW721959 DW722151 DW722769 DW722821 DW722954 DW723561 |
| 1515 | Dr.1124 | no match | CA968068 DW723883 |
| 1486 | Dr.14891 | no match | CF660372 DW723740 |
| 1042 | Dr.47250 | no match | CA966229 CA966205 AU081417 DW720330 DW720925 DW722243 |
| 1351 | Dr.32352 | no match | CF661782 CF661743 CF661647 CF661507 CA966652 AU279285 DW722975 |
| 1411 | Dr.33705 | no match | CA965061 CA964636 DQ173494 DW719678 DW723350 |
| 1600 | Dr.19935 | no match | CF661667 CF662623 CA970257 CA968687 AU301028 DW721017 DW721752 DW722543 DW724074 DW724240 |
