# Supplementary material for: Comparative genomics in cyprinids: common carp ESTs help the annotation of the zebrafish genome
Source: BMC Bioinformatics. 2006 Dec 18;7(Suppl 5):S2. doi: 10.1186/1471-2105-7-S5-S2 (PMC1764476; doi:10.1186/1471-2105-7-S5-S2)
Supplement: Additional File 7 — Homologous gene pairs identified through manual curation of common carp and zebrafish genes. The table includes DNA and protein accession numbers and corresponding gene descriptions. Genes in rows highlighted with yellow contain 5' UTR sequence (>= 50 bp) and were used in UTR analysis. [file 1471-2105-7-S5-S2-S7.doc]

Table S7: Hundred and twenty homologous gene pairs identified through manual curation of common carp and zebrafish genes. The table includes DNA and protein accession numbers and corresponding gene descriptions. Genes in rows highlighted with yellow contain 5’ UTR sequence (>=50bp) and were used in the UTR analysis.

| **ID** | **Common carp DNA accession number** | **Common carp protein accession number** | **Common carp gene description** | **Zebrafish DNA accession number** | **Zebrafish protein accession number** | **Zebrafish gene description** |
| --- | --- | --- | --- | --- | --- | --- |
| 1 | Y10163 | CAA71245.2 | CTH1 | NM_130939.1 | NP_571014.1 | cth1: putative zinc finger protein expressed in oocytes |
| 2 | Z71999 | CAA96518 | rhodopsin | BC045288 | AAH45288.1 | Rho protein |
| 3 | X99910 | CAA68183 | OVX1 | NM_174861.2 | NP_777286 | transcription factor Gbx1 gastrulation brain homeobox 1 |
| 4 | X91079 | CAA62554 | HoxB1splice1 | AL645782 | CAD59118.1 | homeobox protein B1a |
|  | X91079 | CAA62553 | HoxB1splice2 |  |  |  |
| 5 | AY661555 | AAV71059 | Prolactinreceptor-b | AY375318 | AAQ84555.1 | prolactin receptor |
| 6 | AY789468 | AAV52384 | Metallothionein2 | BC051612 | NP_919249 | metallothionein2 |
| 7 | X95431 | CAA64706 | MHCclassIIbeta-chain | AL672158 | CAD87794 | MHC class II beta chain protein |
| 8 | X80668 | CAA56695 | CDX1:Caudalhomolog | NM_131109 | NP_571184.1 | caudal type homeo box transcription factor 4 |
| 9 | X12543 | CAA31060 | Prolactin | NM_181437 | NP_852102.1 | prolactin |
| 10 | X59889 | CAA42543 | gonadotrophin-beta2 | AY714132 | AAV31153.1 | luteinizing hormone beta-subunit; Lhb |
|  | X59888 | CAA42542 | gonadotrophin-beta1 |  |  |  |
| 11 | X55946 | CAA39414 | Gamma-m3-crystallin | NM_001007786 | NP_001007787.1 | Gamma-M3-crystallin |
| 12 | AB194134 | BAD69715 | immunoglobulin-mu-heavy | AY646247 | AAU06708.1 | immunoglobulin mu heavy chain |
| 13 | AY461434 | AAS10175 | uncouplingProtein1 | NM_199523 | NP_955817.1 | uncoupling protein 4 |
| 14 | AJ627274 | CAF28458 | cxcl12a | NM_178307 | NP_840092.1 | chemokine (C-X-C motif) ligand 12a (stromal cell-derived factor 1) |
| 15 | AJ536028 | CAD59917 | cxcl14:smallinduccytokineB14 | BC056594 | AAH56594.1 | Scyba:small inducible cytokine subfamily b |
| 16 | AJ576243 | CAE11291 | corticotropin_releasing_hormone-2 | BC085458 | AAH85458.1 | Corticotropin releasing hormone |
| 17 | AB106873 | BAD51933 | Vitellogenin | NM_170767 | NP_739573.1 | vitellogenin 1 |
| 18 | AJ783918 | CAH04351 | Melanocortin5-receptor-I | NM_173279 | NP_775386.1 | melanocortin 5a receptor |
| 19 | AJ783917 | CAH04350 | Melanocortin5-receptor-II | NM_173280 | NP_775387.1 | melanocortin 5b receptor |
| 20 | AB186387 | BAD35013 | NCCRP1 | NM_130921 | NP_570996.1 | nonspecific cytotoxic cell receptor protein 1 |
| 21 | AY144592 | AAN52152 | ILGF-receptor-1b | NM_152969 | NP_694501.1 | insulin-like growth factor 1b receptor |
| 22 | AY144591 | AAN52151 | ILGF-receptor-1a | NM_152968 | NP_694500.1 | insulin-like growth factor 1a receptor |
| 23 | AB112424 | BAC77690 | TNF3alpha | NM_212859 | NP_998024.1 | tumor necrosis factor alpha |
| 24 | AJ490881 | CAD35749 | corticotrophin-releasing_hormone_bindprot2 | BC076472 | NP_001003459.1 | Corticotrophin-releasing hormone binding protein |
|  | AJ490880 | CAD35748 | corticotrophin-releasing_hormone_bindprot1 |  |  |  |
| 25 | AJ317955 | CAC84859 | Corticotrophin-releasing-hormone | NM_001007379 | NP_001007380 | Corticotrophin-releasing-hormone |
| 26 | AJ605725 | CAE53845 | melanocortin2receptor | NM_180971 | NP_851302.1 | melanocortin 2 receptor |
| 27 | AB006039 | BAD23843 | cERK2:extracell. regul. kinase2 | BC065868 | AAH65868.1 | Mitogen-activated protein kinase 1 |
| 28 | AB006038 | BAD23842 | CERK1 | NM_201507 | NP_958915.1 | mitogen-activated protein kinase 3 |
| 29 | L77976 | AAT00460 | Endozepine | NM_199608 | NP_955902.1 | diazepam binding inhibitor |
| 30 | AB110602 | BAC76806 | opsin1:green-sensitive | AL732567 | CAD87809.1 | opsin 1 (cone pigments), medium-wave-sensitive, 4 (opn1mw4) |
| 31 | BK005098 | DAA04583 | RTN-4 | NM_001004555 | NP_001004555 | zgc:92163 |
| 32 | BK005097 | DAA04582 | RTN-6 | AY555060 | AAT64129 | Reticulon-6 |
| 33 | BK005091 | DAA04576 | RTN-1 | NM_199652 | NP_955946 | Reticulon-1a (rtn-1a) |
|  |  |  |  | NM_001029948 | NP_001025119 | Reticulon-1b (rtn-1b) |
| 34 | AY189961 | AAO39753 | cII_GnRH | AY094357 | AAM15717.1 | cII gonadotropin releasing hormone; cIIGnRH |
| 35 | AY189960 | AAO39752 | salmonType-GnRH | AF490354 | AAL99294.1 | gonadotropin-releasing hormone 3 |
| 36 | AY147400 | AAN64351.3 | cII_GnRH | AY094357 | AAM15717.1 | cII gonadotropin releasing hormone; cIIGnRH |
| 37 | AF001983 | AAB70467 | Mt1 | AY305851 | AAP73739.1 | Mt1 protein metallothionein1 |
| 38 | AB110826 | BAD02477 | mannoseBindingLectin2 | NM_131570 | NP_571645.1 | mannose binding-like lectin |
|  | AB110825 | BAD02476 | mannoseBindingLectin1 |  |  |  |
| 39 | AY460200 | AAR19208 | SHH | NM_131063 | NP_571138.1 | sonic hedgehog vhh-1 - |
| 40 | AF465830 | AAP78926 | ILGF1 | NM_131825 | NP_571900.1 | insulin-like growth factor1 |
| 41 | AF255354 | AAQ14278 | Cytochrome C oxidase Va precursor | BC059554 | AAH59554.1 | Cytochrome C oxidase Va |
| 42 | AF255349 | AAQ14273 | Cytochrome C oxidase VIc | AL929108 | CAE51053.1 | cytochrome c oxidase, subunit VIc (COX6C) |
| 43 | AY421707 | AAQ90410 | D-aminoacid-oxidase | NM_214732 | NP_999897.1 | D-amino acid oxidase |
| 44 | AY395870 | AAR04425 | skeletal_alpha_actin | NM_214784 | NP_999949.1 | Alpha cardiac muscle actin |
| 45 | AY148223 | AAN65463 | CIIGnRH | AY094357 | AAM15717.1 | cII gonadotropin releasing hormone; cIIGnRH |
| 46 | AB075779 | BAC00811 | DnaseI | NM_001002674 | NP_001002674.1 | DNaseI |
| 47 | AB052623 | BAB60809 | warm temp acclimation-related-65-protein | BC056563 | AAH56563.1 | Unknown (IMAGE:6797476) |
| 48 | X55945 | CAA39413 | gammaM1-crystallin | NM_001007785 | NP_001007786.1 | gammaM1-crystallin |
| 49 | AJ536027 | CAD59916.3 | Sdf1b | NM_198068 | NP_932334 | chemokine Sdf1b (stromal cell-derived factor 1) |
| 50 | AJ420957 | CAD12793 | MHC-classIantigen | BC063995 | AAH63995.1 | MHCla-ze protein |
| 51 | AY241101 | AAP04310 | GPR34a | NM_001007215 | NP_001007216.1 | G-protein-coupled receptor GPR34 type 1 |
| 52 | AY241100 | AAP04309 | GPR34_2b | NM_001007216.2 | NP_001007217.1 | G-protein-coupled receptor GPR34 type 2 |
|  | AY241099 | AAP04309 | GPR34_2a |  |  |  |
| 53 | AY330169 | AAQ55855 | nucleolin1 | NM_001003839 | NP_001003839.1 | nucleolin |
|  | AY330168 | AAQ17065 | nucleolin3 | NM_001003839 | NP_001003839.1 | nucleolin |
|  | AY330167 | AAQ17064 | nucleolin2 | NM_001003839 | NP_001003839.1 | nucleolin |
| 54 | AB113669 | BAC78823 | uv-sensitive-opsin1 | BC060894 | AAH60894.1 | Opsin 1(cone pigments), short-wave-sensitive 1 |
| 55 | AB113668 | BAC78822 | blue-sensitive-opsin | NM_131192 | NP_571267.1 | opsin 1 (cone pigments), short-wave-sensitive 2 |
| 56 | AY249415 | AAP35252 | ribosomal_L15 | BC075894 | AAH75894 | zgc:92114 ribosomal protein L15 |
| 57 | AU279339 | BAC76427 | heat-shock-prot4 | BC065970 | AAH65970.1 | Heat shock protein 4 |
| 58 | AF485331 | AAO49408 | EF1_alpha | AY422992 | AAQ97968.1 | eukaryotic translation elongation factor 1 alpha 1 factor 1alpha |
| 59 | AF322651 | AAK18813 | isotocin_precursor | NM_178291 | NP_840076.1 | isotocin neurophysin |
| 60 | AF479820 | AAL87139 | RNA-helicase-DEADbox | Y12007 | CAA72735.1 | RNA helicase (DEAD box) |
| 61 | D37888 | BAA07130 | c-myc2 | BC053281 | AAH53281.1 | Cmyc protein |
|  | D37887 | BAA07129 | c-myc1 |  |  |  |
| 62 | AB098610 | BAC53768 | iclp-2 | NM_131372 | NP_571447.1 | invariant chain-like protein 2 |
| 63 | AB098609 | BAC53767 | iclp-1 | AF114830 | AAD24542.1 | MHC class II-associated invariant chain |
| 64 | AF521130 | AAM77660 | salmon-type-GnRH | AF490354 | AAL99294.1 | gonadotropin-releasing hormone |
| 65 | AF402958 | AAL25799 | Prepro-insulin-like-GF2 | AF250289 | AAM75746.1 | insulin-like growth factor 2 precursor |
| 66 | AJ292212 | CAC83659 | parvalbumin1.02 | NM_212783 | NP_997948.2 | Parvalbumin isoform1c |
| 67 | AJ292211 | CAC83658 | parvalbumin1.01 | NM_200212 | NP_956506.1 | Parvalbumin isoform1b |
| 68 | AJ401031 | CAC19888 | interleukin1-beta2.2 | NM_212844 | NP_998009.1 | interleukin 1, beta |
|  | AJ401030 | CAC19887 | interleukin1-beta2.1 |  |  |  |
|  | AJ245635 | CAB52366 | interleukin1-beta |  |  |  |
| 69 | AJ277123 | CAC12738 | Retinol-bindingprot | AJ236884 | CAB64947.1 | retinol binding protein |
| 70 | AB182405 | BAD82813 | MYH | NM_152982 | NP_694514.2 | myosin, heavy polypeptide 2, fast muscle |
| 71 | AB078926 | BAC06448 | mitochondrial_ATPsynthase_C | NM_131761 | NP_571836.1 | ATP synthase, mitochondrial subunit c |
| 72 | AB047361 | BAB32650 | CcfB-A3 | NM_131338 | NP_571413.1 | complement component factor B |
|  | AB021177 | BAA78416 | CcfB-A2 |  |  |  |
| 73 | AB084624 | BAB91437 | lysozyme_G | NM_001002706 | NP_001002706.1 | lysozyme |
| 74 | AB083064 | BAB91218 | estrogen_receptorBeta | AJ414567 | CAC93849.1 | estrogen receptor beta2 protein |
| 75 | AB027305 | BAA95698 | lysozyme_C | NM_139180 | NP_631919.1 | Lysozyme C |
| 76 | AB026999 | BAA95482 | glia_maturationFactorB | NM_213110** | NP_998275.1 | glia maturation factor Beta |
| 77 | AF132287 | AAD41274 | PTF Pit-1 | NM_212851** | NP_998016.1 | transcription factor 1 pituitary-specific transcription factor |
| 78 | AY044448 | AAK95833 | prolactin_receptor_precursor | AY375318 | AAQ84555.1 | prolactin receptor |
| 79 | AB063102 | BAB79239 | beta_globin_type3 | AF082662 | AAC62069.1 | embryonic 1 beta-globin |
| 80 | AB063101 | BAB79237 | alpha-globin-type2 | BX004811 | CAE30444.1 | novel alpha-globin |
| 81 | AB063102 | BAB79240 | alpha_globin_type3 | AY325264 | AAP78902 | Embryonic alpha globin e1 |
| 82 | AF309557 | AAG25711 | Fadsd6 | AF309556 | AAG25710.1 | fatty acyl desaturase delta-6 |
| 83 | AF414432 | AAL07472 | vitellogenin | NM_170767 | NP_739573.1 | vitellogenin 1 |
| 84 | AF414052 | AAL02240 | transglutaminase | NM_212656 | NP_997821.1 | protein-glutamine gamma-glutamyltransferase |
| 85 | AF253045 | AAK49117 | Spleen_PTK | NM_212843 | NP_998008.1 | spleen tyrosine kinase |
| 86 | AY008286 | AAG17938 | connexin_43 | AY313942 | AAQ62128.1 | connexin 43 Gap junction alpha-1 (Cx43) |
| 87 | AF227737 | AAF63468 | mannose-bind-like-lectin | NM_131570 | NP_571645.1 | mannose binding-like lectin |
| 88 | AB042438 | BAB47390 | mATPase_gamma | NM_200041 | NP_956335.1 | mitochondrial ATP synthase gamma-subunit |
| 89 | AB057407 | BAB39390 | Matrix_metalloproteinase9 | BC053292 | AAH53292.1 | matrix metalloproteinase 9 |
| 90 | AB055656 | BAB32496 | red-opsin1 | AL844847 | CAE30416.1 | opsin 1 (cone pigments), long-wave-sensitive, 1 |
| 91 | AF287347 | AAG00549 | neuropeptideY | NM_131074 | NP_571149.1 | neuropeptide Y |
| 92 | AB036771 | BAA99395 | myc_assocX | AL928866 | CAI20587.1 | myc-associated factor X |
| 93 | AF233520 | AAF74260 | vertebrate_ancientOpsin | NM_131586 | NP_571661.1 | vertebrate ancient long opsin |
| 94 | AB023481 | BAA96415 | MAPKp38 | BC063029 | AAH63029.1 | Mapk14b protein |
| 95 | AB023480 | BAA96414 | MAPKK6 | NM_131724 | NP_571799.1 | mitogen-activated protein kinase kinase 3 |
| 96 | AF133088 | AAF66446 | CK2-beta | NM_131187 | NP_571262.1 | casein kinase 2 beta |
| 97 | AB037014 | BAA89705 | MYL-a | NM_131188 | NP_571263.1 | myosin, light polypeptide 2, skeletal muscle |
|  | AB037013 | BAA89704 | MYL-b |  |  |  |
| 98 | AF136947 | AAF22573 | Transforming growth_factor_b1 | NM_182873 | NP_878293.1 | transforming growth factor, beta 1 |
| 99 | AJ133735 | CAB60196 | natural_resistance_assoc_macro-phage protein (NRAMP) | AF529267 | AAO16988.1 | divalent metal transporter1;Nramp2; slc11a2 |
| 100 | AJ249259 | CAB57858 | Desaturase-2 | AY217090 | AAO25582.1 | stearoyl-CoA desaturase |
|  | U31864 | AAB03857 | stearoyl-CoA_desaturase |  |  |  |
| 101 | AJ243486 | CAB46248 | Uncoupling_protein2 | BC065607 | AAH65607.1 | Uncoupling protein 2 |
| 102 | AF076528 | AAD40736 | Lactate_dehydrogenaseA | NM_131246 | NP_571321.1 | lactate dehydrogenase A4 |
| 103 | AY956415 | AAX56088 | Sox9b | BC067133.1 | AAH67133 | SRY-box containing gene 9b |
| 104 | AY574056 | AAS75816 | metal_response-element-bindingTF-1 (MTF) | NM_152981.1 | NP_694513.1 | metal-regulatory transcription factor 1 |
| 105 | AY949988 | AAX51298.1 | cathepsinZ | BC083369 | NP_001006043.1 | Zgc:103420 |
| 106 | U53783.1 | AAD10840.1 | Rap1b | AY423018.1 | AAQ97994.1 | RAP1B, member of RAS oncogene family |
| 107 | AF055290 | AAC96094.1 | creatinekinase-M3-CK | BC085628.1 | NP_998286.1 | CKM3 |
| 108 | AF055289 | AAC96093.1 | creatinekinase-M2-CK | AY036972 | NP_571007.1 | creatine kinase, muscle |
|  | AF055288 | AAC96092.1 | creatinekinase-M1-CK |  |  |  |
| 109 | AB007004 | BAA34706.1 | complementfactorB/C2A | U34662.1 | NP_571413.1 | complement component factor B |
|  | AB007005 | BAA34707.1 | complementfactorB/C2B |  |  |  |
| 110 | Y14618 | CAA74968.1 | Pro-opiomelanocortin-I | AY135148 | NP_852103.1 | Pro-opiomelanocortin |
| 111 | AB012885 | BAA33568.1 | MEF2C | BC059188 | NP_571387.2 | Myocyte enhancer factor 2c |
| 112 | D85141.1 | BAA12733.1 | fast-skeletal-myosin-lightchain-3 | AF180891 | NP_571694.1 | myosin, light polypeptide 3, skeletal muscle |
| 113 | D85140.1 | BAA12732.1 | fast-skeletal-myosin-lightchain-1b | BC065629.1 | AAH65629 | ZGC:77231 |
| 114 | D85139.1 | BAA12731.1 | fast-skeletal-myosin-lightchain-1a | BC059795 | NP_956294.1 | fast skeletal myosin light chain 1a |
| 115 | Z72491 | CAA96572 | ZP2a | NM_131827.1 | NP_571902.1 | zona pellucida glycoprotein 2.2 |
| 116 | D83274 | BAA11881 | mitogen-activated protein kinase (p38)  (unique in ovary) | NM_131722 | NP_571797.1 | mitogen-activated protein kinase 14a (mapk14a) |
| 117 | L41639 | AAB41822 | ZP3 | BC067701 | AAH67701.1 | Zp3 protein |
| 118 | U00432 | AAA19565 | ependyminprecursor | NM_131005 | NP_571080.1 | ependymin |
| 119 | S76850 | AAB20706 | vimentin | NM_131872** | NP_571947.1 | vimentin |
| 120 | M14088 | AAA49216 | urotensinII-gamma | AY305004 | AAP69834.1 | urotensin II alpha precursor |
